# Developments in data science solutions for carnivore tooth pit classification

**DOI:** 10.1038/s41598-021-89518-4

**Published:** 2021-05-13

**Authors:** Lloyd A. Courtenay, Darío Herranz-Rodrigo, Diego González-Aguilera, José Yravedra

**Affiliations:** 1 Department of Cartographic and Terrain Engineering, Higher Polytechnic School of Ávila, University of Salamanca, Hornos Caleros 50, 05003 Ávila, Spain; 2 Department of Prehistory, Complutense University, Prof. Aranguren s/n, 28040 Madrid, Spain; 3 C. A. I. Archaeometry and Archaeological Analysis, Complutense University, Professor Aranguren 2/n, 28040 Madrid, Spain

**Keywords:** Palaeoecology, Archaeology, Computational models, Statistics

## Abstract

Competition for resources is a key question in the study of our early human evolution. From the first hominin groups, carnivores have played a fundamental role in the ecosystem. From this perspective, understanding the trophic pressure between hominins and carnivores can provide valuable insights into the context in which humans survived, interacted with their surroundings, and consequently evolved. While numerous techniques already exist for the detection of carnivore activity in archaeological and palaeontological sites, many of these techniques present important limitations. The present study builds on a number of advanced data science techniques to confront these issues, defining methods for the identification of the precise agents involved in carcass consumption and manipulation. For the purpose of this study, a large sample of 620 carnivore tooth pits is presented, including samples from bears, hyenas, jaguars, leopards, lions, wolves, foxes and African wild dogs. Using 3D modelling, geometric morphometrics, robust data modelling, and artificial intelligence algorithms, the present study obtains between 88 and 98% accuracy, with balanced overall evaluation metrics across all datasets. From this perspective, and when combined with other sources of taphonomic evidence, these results show that advanced data science techniques can be considered a valuable addition to the taphonomist’s toolkit for the identification of precise carnivore agents via tooth pit morphology.

## Introduction

Throughout history, humans and carnivores have been documented to have complex relationships^1–4^. From a more traditional perspective, competition for resources is the most documented^4^. Nevertheless, conflict between these taxonomic orders is also well known, especially in the context of dynamic shifts in who plays the role of predator and who plays the role of prey^1,5–9^. Among the many sites of global importance, interactions of these types have been documented across most continents, including notable cases from the Olduvai Gorge (Tanzania)^4,8^, Thomas Quarry (Morocco)^9^, Schöningen (Germany)^7,10^, Zhoukoudian (China)^11^, and the classic sites of Makapansgat (South Africa)^1^. Moreover, in more recent periods collaboration between these two orders have also been recorded^2^.

From multiple perspectives, carnivore–hominin interactions have thus been a topic of great interest, in both the study of how humans survived and adapted, as well as the contexts in which this occurred. These types of analyses, however, have not been free of debate. In certain case studies, issues of equifinality have led analysts to propose problematic interpretations. The famous long bone fragment from Divje Babe (Slovenia) was originally interpreted as a 43 Ka Middle Palaeolithic flute. Nevertheless, subsequent analyses have discredited these finds and found the perforations to be product of carnivore bite damage^12,13^. Likewise, the sites of Sima de los Huesos (Atapuerca, Spain) and the Dinaledi Rock Chamber (South Africa), have been interpreted as the deliberate anthropic accumulations of human remains^14,15^. Needless to say, not all researchers agree with these conclusions^5,16^.

The discipline of taphonomy has frequently been at the forefront of these debates^4^. Taphonomy employs numerous tools for the detection, documentation, and consequent interpretation of carnivore and human activities involved in the formation of a site^2,3^. Nevertheless, diagnostic tools are frequently subjective, thus requiring a search for more empirical and accurate techniques in the identification and interpretation of Bone Surface Modifications (BSM)^17^. This is especially relevant when considering techniques available for discerning of the precise carnivore agencies involved in site formation processes.

Geometric Morphometrics (GM) are a popular multivariate statistical tool for the analysis of morphological variance typically in biological systems^18,19^. Recent years, however, have seen an increase in GM applications outside of anatomy. Applications in taphonomy have yielded impressive results when using GM as a tool for morphological analyses and visualisation. From this perspective, multiple attempts have been made to use GM as a diagnostic tool in carnivore taphonomy^8,20–25^. With the inclusion of Machine Learning (ML) algorithms, data presented by Courtenay et al.^20^ present a promising advance for the integration of Artificial Intelligence (AI) and advanced Data Science techniques with GM. Nevertheless, considering the relatively small sample size, these results can also be considered optimistic. Likewise, in a recent study the original landmark model proposed^24^ was found to present important margins of error product of landmark quality. These observations infer that analyst experience condition the quality of results^21^.

Under this premise, the present study uses an updated version of the landmark model using semi-landmarks^21^, and a much larger sample size to expand on the current referential samples available for taphonomic analyses. These efforts aim to provide high quality data that can aid in the understanding of modern carnivore taxa that are frequently found across Eurasia, Africa and the Americas. Samples include three types of felids (*Panthera leo, Panthera onca* & *Panthera pardus*), three types of canids (*Canis lupus, Vulpes vulpes* & *Lycaon pictus*), the spotted hyena (*Crocuta crocuta*), and the brown bear (*Ursus arctos*), that have been frequently subject of study in Pleistocene research^1,26–36^. This larger sample allows us to conclude that > 90% separation of carnivore taxa is still possible, with possibilities for even higher classification rates in the future.

## Results

### Geometric morphometrics

**Figure 1:**
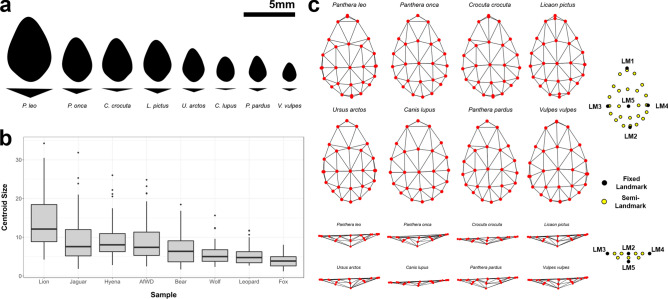
Variations in Form and Shape across tooth pits made by different taxa. (**a**) Variations in mean shape-size relationships (top-view used for general morphology and front-view for depth). (**b**) Boxplot diagrams representing centroid size distributions for each species (See Supplementary Table 1). (**c**) Mean landmark configurations for carnivore tooth pits using Delaunay 2.5D Triangulation algorithms for mesh visualisations. AfWD = *Lycaon pictus*. Figures created using the ggplot2 and scikit-learn Python and R libraries.

All samples are described by notable allometric patterns (Squared Residuals = 0.006, F = 4.1, Effect Size = 2.7, *p* = 0.005, Bayes Factor Bound (BFB) = 13.88 against $H_{0}$), indicating tooth pit size to be an important conditioning factor in morphological variation. This is equally reflected when simply considering Centroid Size values for each of the carnivores (Fig. 1, Table S1), with suggestive to strongly indicative differences detected across most species ($\chi ^{2}$ = [5.08, 85.03], $p < 0.007$, BFB > 10.59). Exceptions to this include *C. crocuta*, *L. pictus* and *P. onca* when these taxa are compared together ($\chi ^{2}$ = [0.14, 1.21], *p* > 0.27, BFB < 1.04), as well as *P. pardus* when compared with *C. lupus* ($\chi ^{2}$ = 0.42, *p* = 0.51, BFB = 1.07 against $H_{a}$).

When considering multivariate morphological tendencies in form, general patterns reveal significant differences throughout comparisons, with each of the taxonomic families being clearly separable (*p* $\approx$ 0.001, BFB $\approx$ 53.25). While the statistical separation was weakest when comparing Canidae and Ursidae (*p* = 0.003, BFB = 21.11), as well as Ursidae and Hyaenidae (*p* = 0.006, BFB = 11.98), in both these cases differences remain of notable interest ($p < 0.05$). From a similar perspective, species within the families Canidae and Felidae appear easily separable (*p* = 0.001, BFB = 53.25). When describing patterns of variation on a species-specific level, most carnivores present statistical differences (*p* $\approx$ 0.001, BFB $\approx$ 53.26, Table S2 & S3), nevertheless, exceptions to this can still be found. From this perspective, some degrees of equifinality are therefore still likely to exist when comparing *L. pictus*, *C. crocuta* and *P. onca* (*p* > 0.8, BFB > 2.06 against $H_{a}$), as well as when comparing *C. crocuta* and *P. onca* (*p* = 0.17, BFB = 1.22).

Exploring morphological variation through visualisations of mean landmark configurations reveal that the greatest differences appear when considering landmark displacements across the *z*-axis (Fig. 1). From this it can be seen that *C. lupus* tend to leave the most superficial traces, while *P. leo* leave some of the deepest and largest tooth pits of the entire sample. Interestingly, *V. vulpes* and *L. pictus* appear to leave very deep pits in relation to their size. Likewise, when considering variations across a horizontal plane (*x* and *y* axes), slight variations can be seen with some of the canids such as *C. lupus* and *V. vulpes* leaving more circular marks, while felids appear to leave more elongated pits (Fig. 1).

When analysing these central morphological tendencies in accordance with taxonomic groupings, very weak phylogenetic signals are detected, indicating other confounding variables, such as biomechanics, exert a much stronger influence on tooth pit formation than cuspid morphology (Effect Size = − 0.99, *p* = 0.81, BFB = 2.16 against $H_{a}$. Fig. S1).

### Unsupervised computational learning

Dimensionality reduction of datasets through Principal Components Analysis (PCA) produced high dimensional, non-homogeneously distributed and noisy datasets on all accounts. General analyses showed PCA in form space to produce a total of 90 Principal Component (PC) Scores, of which the first 6 PC Scores represent over 95% of the total sample variance. Analyses of optimal number of components observed 5 PC scores to be the most representative. Nevertheless high residuals were still noted across a number of these dimensions.

**Figure 2:**
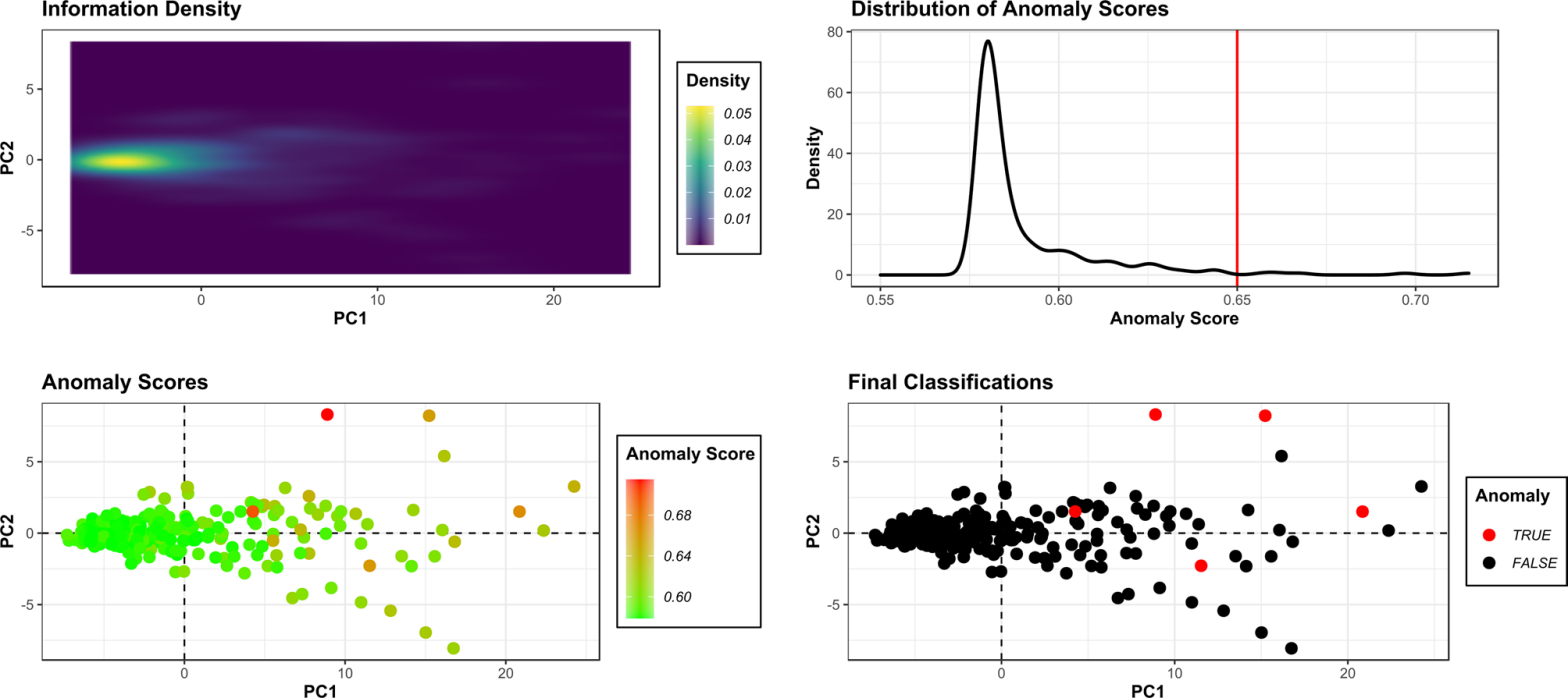
Anomaly detection results using Isolation Forests. Top Left Panel: Density of information within Principal Components Analysis. Top Right Panel: Distribution of Anomaly Scores; Vertical red line marks the acceptable threshold. Bottom Left Panel: Scatter plot heat map indicating the anomaly scores for each point. Bottom Right Panel: Final classifications of points as anomalies (True) or not (False). Figure created using the ggplot2 R library.

When preparing datasets for further processing, Isolation Forests (IF) proved effective for the elimination of anomalies across all 5 dimensions (Fig. 2). Nevertheless, a relatively high anomalous score threshold was needed for most anomaly detection tasks, considering how species like *P. leo* and *C. lupus* presented very high variability in comparison with other samples. This natural variability consequently produced a global increase of variance across all dimensions, frequently resulting in the adversarial effect of IFs over-classifying entire species as anomalies due to their abnormally large morphological variations. Under this premise, anomaly score distributions were allowed a slight positive tail, with thresholds in the present study defined between 0.625 and 0.700. Using these thresholds, IFs were seen to remove between 3 and 10 pits for each dataset, with the most extreme removal of 10 pits occurring in the European Taxa dataset. Nevertheless, upon inspection of anomaly score distributions (Top right panel; Fig. 2), it can be argued that IFs were still able to preserve the majority of natural variability, only eliminating the most extreme of cases. In light of this, IFs were only seen to remove at most 2.3% of the original sample.

**Figure 3:**
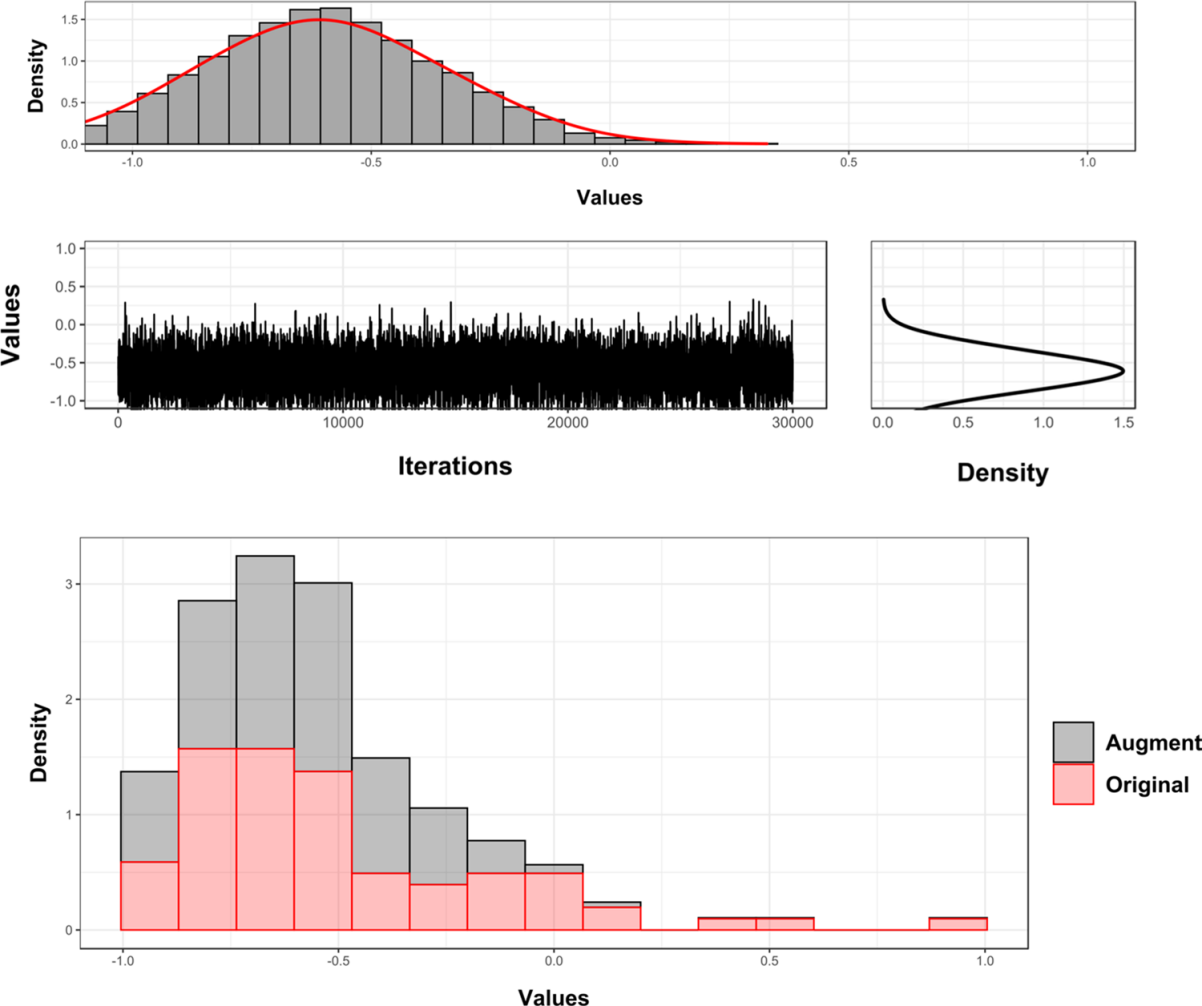
Example of trace figures, target density and histograms of the augmented and original datasets as generated using Markov Chain Monte Carlo algorithms. Figure created using the ggplot2 R library.

Once datasets had been cleaned, data augmentation proved successful on all accounts with the generation of highly realistic synthetic data by both algorithms. Of the two algorithms tried and tested, Markov Chain Monte Carlo (MCMC, Fig. 3) algorithms appeared the fastest at generating new data with very high equivalency scores (Table 1). Experimentation found MCMCs to produce the most realistic data when sampling from robustly defined gaussian target distributions ($\left| d \right|$ = 0.004, *p* = 1.2e−57, BFB = 2.3e+54), as opposed to the skewed-normal ($\left| d \right|$ = 0.06, *p* = 1.3e−05, BFB = 2515). This was especially evident when considering the skewed-normal had the tendency to exaggerate non-Gaussian elements, which may not be a true reflection of the population distribution (original skew = 0.18, augmented skew = 0.97).

From the perspective of generative neural networks, of the three Generative Adversarial Networks (GANs), Wasserstein Gradient-Penalty loss GANs (WGAN-GP) produced the best results ($\left| d \right|$ = 0.012, *p* = 2.4e−13, BFB = 5.3e+10). Nevertheless, while WGAN-GP proved successful on all datasets, the training of GAN models proved to be computationally expensive, with iterations taking $\approx$25,000 times longer than MCMC ($\chi ^{2}$ = 5.6, *p* = 0.018, BFB = 5.10).

**Table 1:** Examples of absolute difference ($\left| d \right|$), *p*-Values and Bayes Factor Bounds (BFB) obtained when assessing the robust equivalency of synthetic data and real data using Gradient Penalty Wasserstein Loss Generative Adversarial Networks (WGAN-GP) and Markov Chain Monte Carlo (MCMC) Algorithms for data augmentation of the African Taxa dataset. Time values reported represent the number of miliseconds per epoch or iteration of the algorithm.

Algorithm	Animal	Measure	PC1	PC2	PC3	PC4	PC5	Time (Ms)
WGAN-GP	*C. crocuta*	$\left| d \right|$	0.007	0.011	0.016	0.115	0.037	
		*p*	4.9e−13	2.7e−09	6.8e−16	1.3e−08	8.9e−22	1311
		BFB	2.6e+10	6.9e+06	1.5e+13	1.6e+06	8.5e+18	
	*P. pardus*	$\left| d \right|$	0.092	0.010	0.022	0.007	0.016	
		*p*	3.4e−18	3.6e−37	2.9e−32	1.5e−36	1.4e−36	1194
		BFB	2.7e+15	1.2e+34	1.7e+29	3.0e+33	3.2e+33	
	*L. pictus*	$\left| d \right|$	0.034	0.009	0.046	0.001	0.004	
		*p*	1.2e−08	1.0e−12	1.4e−19	4.8e−33	4.0e−16	1296
		BFB	1.7e+06	1.3e+10	6.1e+16	1.0e+30	2.6e+13	
	*P. leo*	$\left| d \right|$	0.097	0.005	0.008	0.043	0.003	
		*p*	6.3e−03	5.5e−10	5.8e−10	1.5e−08	1.6e−15	915
		BFB	11.52	3.1e+07	3.0e+07	1.4e+06	6.7e+12	
MCMC	*C. crocuta*	$\left| d \right|$	0.055	0.010	0.010	0.004	0.003	
		*p*	5.8e−13	4.3e−40	8.5e−73	6.0e−63	3.6e−62	0.048
		BFB	2.3e+10	9.4e+36	2.6e+69	4.3e+59	7.2e+58	
	*P. pardus*	$\left| d \right|$	0.004	0.007	0.004	0.003	0.007	
		*p*	6.9e−29	4.8e−70	1.5e−104	2.8e−75	1.0e−85	0.048
		BFB	8.2e+25	4.8e+66	1.0e+101	7.7e+71	1.9e+82	
	*L . pictus*	$\left| d \right|$	0.007	0.010	0.003	0.004	0.003	
		*p*	9.6e−12	3.3e−42	4.5e−83	1.7e−67	2.5e−57	0.047
		BFB	1.5e+09	1.2e+39	4.3e+79	1.4e+64	1.1e+54	
	*P. leo*	$\left| d \right|$	0.023	0.010	0.002	0.004	0.001	
		*p*	1.1e−05	1.0e−32	3.4e−54	8.6e−39	1.5e−50	0.048
		BFB	2.9e+03	5.0e+29	8.8e+50	4.9+e35	2.1e+47	

For final data augmentation tasks both MCMC and WGAN-GP were used, with the best performing algorithm being chosen to augment each dataset prior to supervised training (Tables 1, S4-7).

### Supervised computational learning

**Figure 4:**
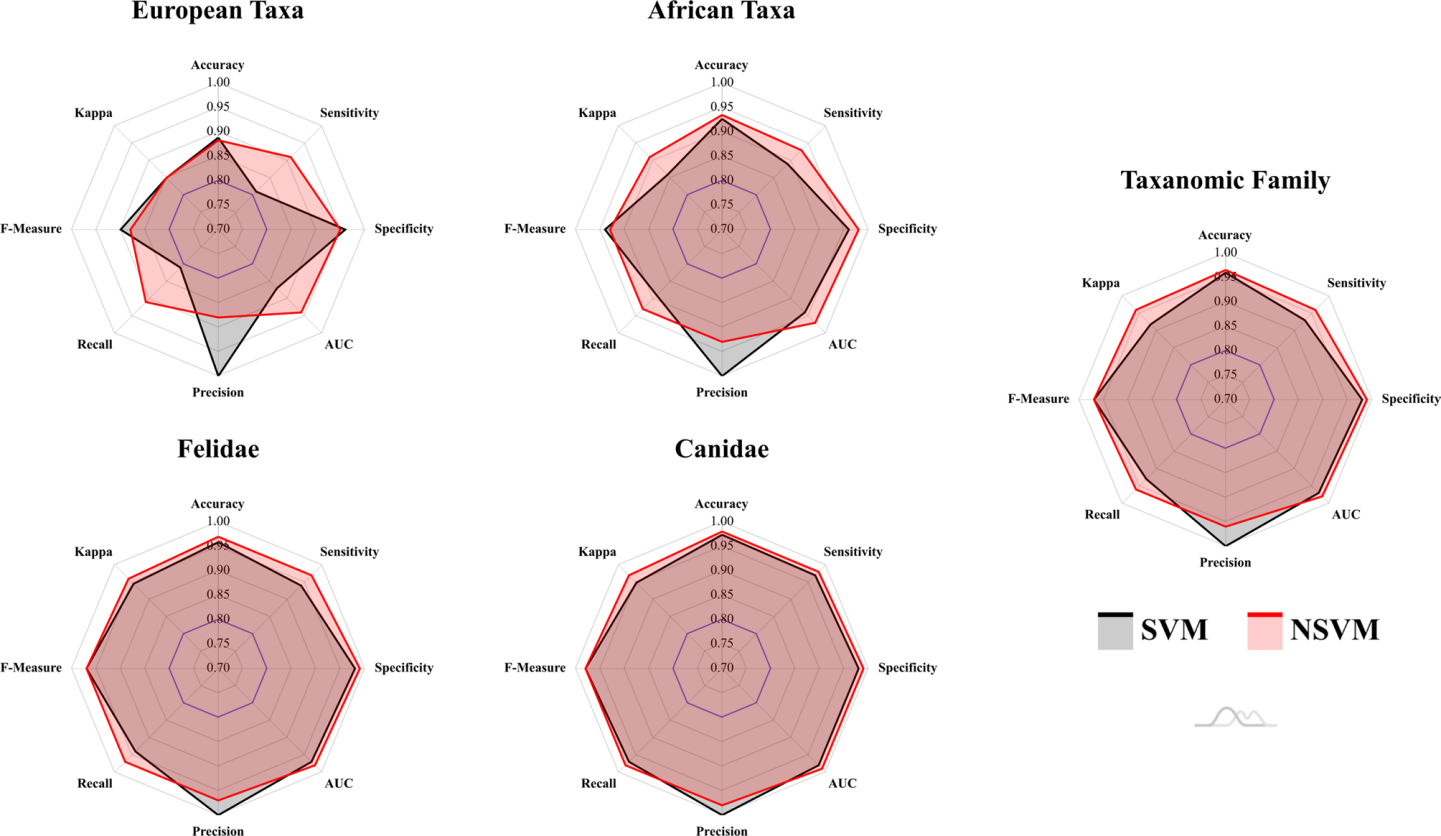
Radar plots representing supervised classification results for each of the datasets. Evaluation metrics were calculated on test sets when using both Support Vector Machines (SVM) and Neural Support Vector Machines (NSVM). The blue line marking 0.8 across all graphs represents a standard threshold for the evaluation of good performance for each of the metrics used. Figure created using the amCharts4 JavaScript library.

**Figure 5:**
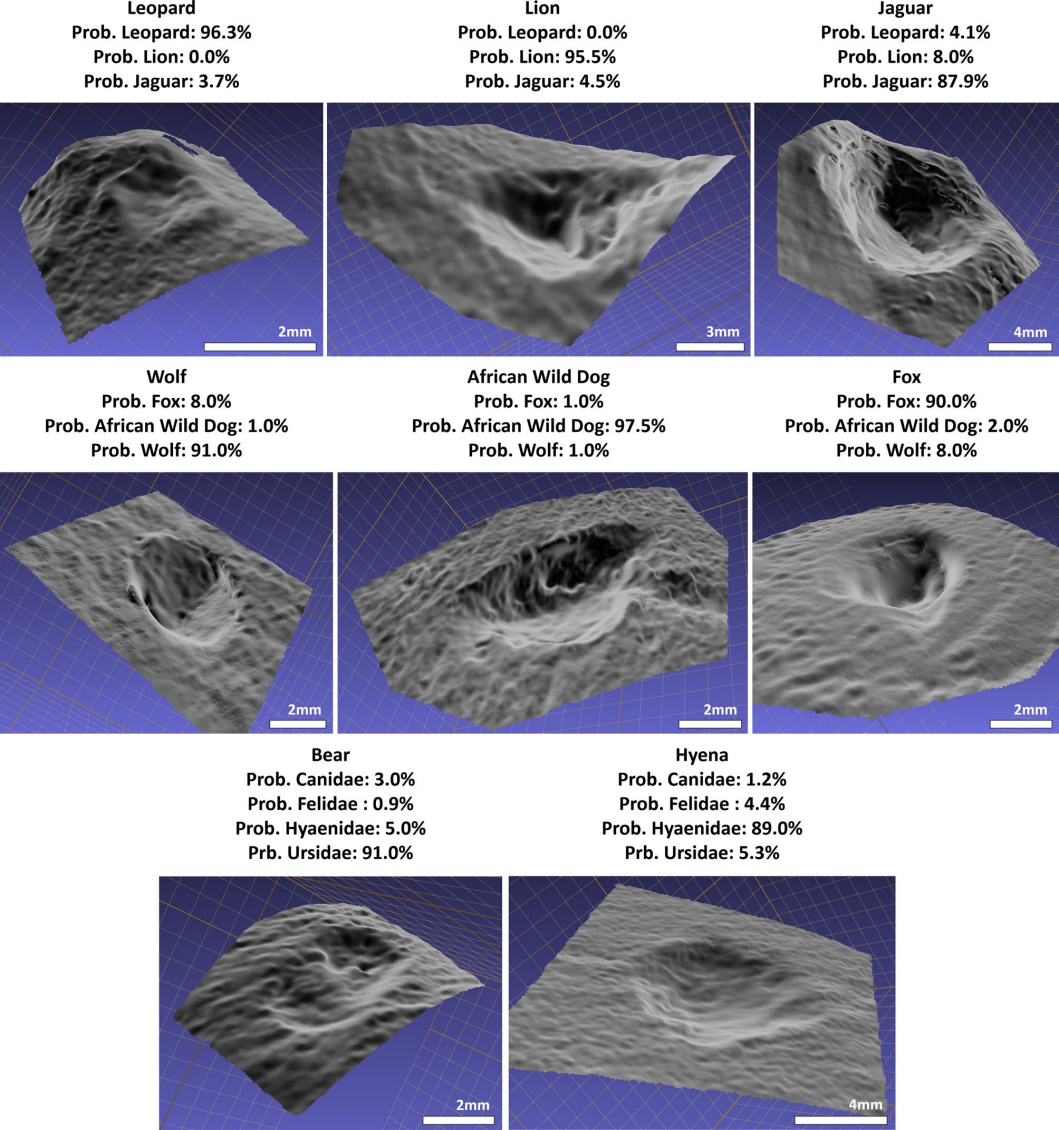
Example of tooth pit classifications using Neural Support Vector Machines (NSVM). The select tooth pits were chosen randomly and excluded from the training data so as to avoid bias. NSVMs were then trained on the remaining data and used to classify the present tooth marks, taking note of the algorithms confidence when making predictions. 3D visualisations were created using MeshLab.

**Figure 6:**
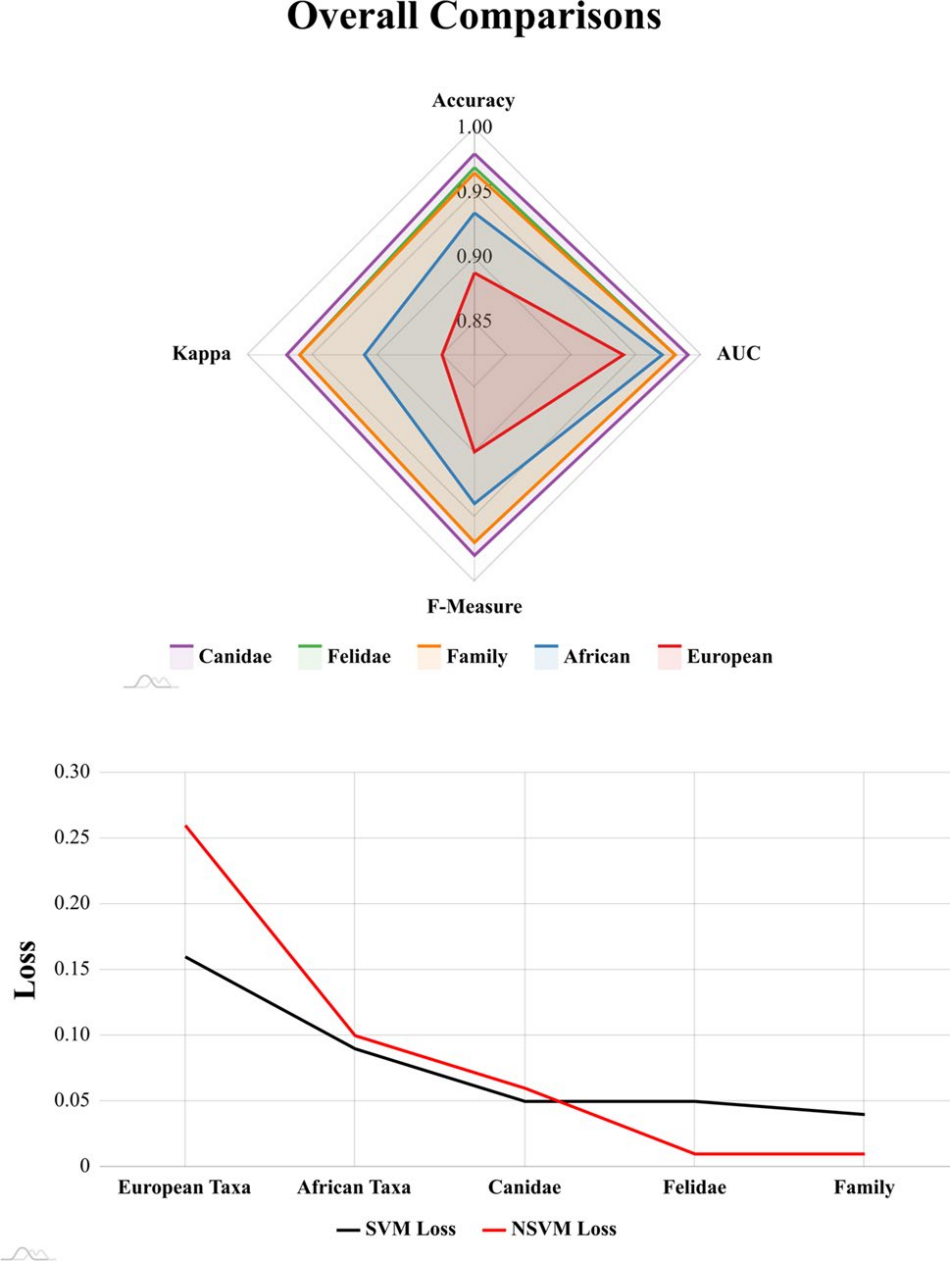
Top Panel: Radar plot summarising and comparing performance of the best computational learning models for each of the datasets. Bottom panel: Line graph representing the mean reported loss for both Support Vector Machines (SVM) and Neural Support Vector Machines (NSVM) on each of the datasets. Figure created using the amCharts4 JavaScript library.

Both supervised models provided high accuracy in the classification of carnivore taxa (Tables 2 & S8-12, Figs. 4, 5 and 6), in most cases producing >90% accuracy (Area Under Curve (AUC) > 0.94, F-Measure > 0.93, $\kappa$ > 0.86). The only exception to this can be found in the case of the Pleistocene European Taxa dataset, which only produced >85% accuracy (AUC $\approx$ 0.90, F $\approx$ 0.89, $\kappa$ $\approx$ 0.85). Upon analysing the overall performance of each dataset, the greatest results are obtained when differentiating between taxonomic families (Accuracy > 96%, AUC > 0.97, F > 0.97, $\kappa$ > 0.92), as well as the specific species within these families (Table S11 & S12). This can be seen in the cases of the Canidae (Acc. > 97%, AUC > 0.98, F > 0.98, $\kappa$ > 0.95), and the Felidae datasets (Acc. > 96%, AUC > 0.97, F > 0.97, $\kappa$ > 0.95).

When pooling many labels, especially with taxa from different families, overall classification rates tend to drop. Nevertheless, while classification rates may fall below 90% accuracy, miss-classification rates and the frequency of Type I and Type II errors do not rise above 0.2 when considering overall performance (Fig. 4), resulting in very high AUC, Kappa and F scores as well. Under this premise, both Support Vector Machines (SVM) and Neural SVMs (NSVM) can be considered highly efficient classifiers of carnivore tooth marks, yet with greater performance when working with a smaller number of labels. Needless to say, when considering loss values, with the exception of the Pleistocene European dataset, both SVM and NSVM appear to be confident when making new predicitons (Fig. 6).

By considering model performance on individual samples (Tables S8-S12), differentiating between taxa appears to depend on the species being used for comparison. Under this premise, *V. vulpes* (Tables S8) and *P. leo* (Tables S9) appear to be the easiest of the Pleistocene European and African carnivores to identify (SVM Acc. = $\{95\%, 95\%\}$, NSVM Acc. = $\{94\%, 96\%\}$, respectively). On the scale of taxonomic families, *L. pictus* can be considered the easiest canid to identify (SVM Acc. = 98%, NSVM Acc. = 100%), while *P. leo* remains the felid with the highest classification rates (SVM Acc. = 96%, NSVM Acc. = 99%). Each of these observations are especially interesting considering these species have been associated with either the largest or the smallest centroid sizes respectively (Fig. 1, Table S1).

While *P. pardus* presents the lowest recorded individual classification rates across all datasets (NSVM Acc. = 0.79, Table S8), this does not have a significant impact on overall model performance (Fig. 4). Even when considering the poorer classification rates presented by *P. pardus*, all algorithms achieve evaluation metrics above the acceptable 0.8 threshold. Likewise, a 97% to 99% accuracy has still been obtained when comparing *P. pardus* with other felids (Fig. 5), and a 92% to 93% accuracy when compared with other African species.

Although an element of equifinality is still present, as detected through inconclusive statistical differences in some tooth pit morphologies, both SVM and NSVM are still able to accurately differentiate between *L. pictus* and *C. crocuta* with over 90% success. Nevertheless, algorithm confidence when performing classifications on these species drops, as seen through a large increase in loss values (Table S9). This results in the overall rise in loss and decrease in other performance metrics when these two species are included in a dataset (Table 2, Fig. 6).

Observations comparing SVM with NSVM prove both algorithms to be equally powerful when discerning between carnivore taxa. While NSVM may be seen to have a slight advantage over SVM in some evaluation metrics (Fig. 4), SVM loss rates are generally lower (Fig. 6). Similiarly, NSVM can be seen in some datasets to have very low loss rates for some groups (e.g. Table S10, Canidae loss = 0.001), while especially high loss rates for others (e.g. Table S10, Hyaenidae loss = 0.19). In sum, both SVM and NSVM are valid options for carnivore differentiation, while choice of one or the other must be dependent on the specific case study at hand as well as the analyst’s needs.

When observing general performance in model loss (Fig. 6), algorithms produce powerful predictions, with very confident decision boundaries in many cases (Fig. 5).

Finally, when training algorithms without the use of data augmentation (Sup. Appendix 7), it can be seen how the average accuracy slightly drops, with SVM performing 4% worse on non-augmented datasets and NSVM performing 6% worse. While this change is minute, the greatest differences between augmented and non-augmented datasets can be found across loss values, with both SVM and NSVM loosing an average of 10% confidence with each prediction made. As would be expected, algorithms also appear to perform worse on unbalanced datasets, with the Taxonomic Family dataset presenting F-Measure values 0.25 lower, especially in the case of NSVM (Sup. Appendix 7).

**Table 2:** Overall classification results obtained for all samples using Support Vector Machines (SVM) and Neural Support Vector Machines (NSVM). Reported values include; Accuracy (Acc.), Sensitivity (Sens.), Specificity (Spec.), Precision (Prec.), Recall (Rec.), Area Under Curve (AUC), F-Measure (F), Kappa ($\kappa$) and Loss. All evaluation metrics (with the exception of loss) are recorded as values between 0 and 1, with 1 being the highest obtainable value. Values reported over 0.8 are considered an acceptable threshold for powerful classification models. Loss considers values closer to 0 as the most confident models.

Sample	Algorithm	Acc.	Sens.	Spec.	Prec.	Rec.	AUC	F	$\kappa$	Loss
Pleistocene European Taxa	SVM	0.89	0.81	0.96	1.00	0.81	0.87	0.90	0.85	0.16
	NSVM	0.88	0.91	0.95	0.88	0.91	0.94	0.88	0.85	0.26
African Taxa	SVM	0.93	0.89	0.96	1.00	0.89	0.94	0.94	0.86	0.09
	NSVM	0.93	0.93	0.98	0.93	0.93	0.97	0.93	0.91	0.10
Taxonomic family	SVM	0.96	0.93	0.98	1.00	0.93	0.97	0.97	0.92	0.05
	NSVM	0.97	0.96	0.99	0.96	0.96	0.98	0.97	0.96	0.06
Canidae	SVM	0.97	0.97	0.98	1.00	0.97	0.98	0.98	0.95	0.05
	NSVM	0.98	0.98	0.99	0.98	0.98	0.99	0.98	0.97	0.01
Felidae	SVM	0.96	0.94	0.98	1.00	0.94	0.97	0.97	0.95	0.04
	NSVM	0.97	0.97	0.99	0.97	0.97	0.98	0.97	0.96	0.01

## Discussion

In recent years, GM have been applied to a wide array of different applications outside of biology. Among these applications, these tools have shown promising results when applied to the study of BSMs^8,20–25,37^. While subsequent analyses have identified some issues with these techniques for carnivore BSM applications^21^, the present study has shown that high quality results are still realistically obtainable (Accuracy > 90 %, AUC > 0.8, $\kappa$ > 0.8, Fig. 6). Likewise, the results reported here are supported by considerably larger sample sizes^20,22,24^.

Here we have shown how a number of different data science tools can be employed for GM analyses. From one perspective, unsupervised computational learning approaches were able to produce highly realistic augmented datasets, using both neural network based approaches^38–41^, as well as Bayesian Inference Engines^42–45^. While the use of Graphics Processing Units (GPUs) are likely to speed up GAN performance, MCMC can still be considered the fastest approach to modelling these datasets with exceptional synthetic-data quality. From a Bayesian perspective, considering how the use of Gaussian distributions is usually seen as a “crude approximation” to the problem solving questions at hand^45^, most of the times this also allows models greater generalization capabilities. In addition, this theoretically reduces chance of overfitting supervised models on one particular skewed distribution that may not be a true reflection of the population distribution (Sup. Appendix 4). Moreover, to ensure the present study does not fall into the trap of over-generalising the Gaussian nature of the population, the precise definitions of our target probability distributions were robustly defined^41,46,47^.

From the perspective of supervised learning, the present study reveals the capabilities of computational learning algorithms for the differentiation of carnivore taxa based on the morphology of carnivore tooth pits. Firstly, prior augmentation of each dataset provided both algorithms with enough information to learn from, obtaining above average accuracy when used to classify the original samples. While the present datasets are unable to reach the 100% accuracy reported originally using SVMs^20^, this is likely due to the use of bootstrapping in the original study^41^. Here, more robust data augmentation techniques produced completely new synthetic data from which to learn from, providing a more general overview of the target domain. Under this premise, while 100% accuracy was not obtained, our reported >90% can be considered much more reliable. From a similar perspective, while the changes to the original landmark model have shown a reduction in inter-analyst error by 164$\upmu$m^21^, the inclusion of semi-landmark patches has been observed to substantially increase the dimensionality of these GM datasets. In light of this, the new datasets are likely to be harder to model from. Needless to say, considering the increased precision of the landmark model, alongside more robust augmentation techniques, it can be argued that the present results are not only more reliable, but also worth the slight drop in accuracy.

Despite the increase in landmark model complexity, both Radial kernel functions and Laplacian fourier mappings were able to provide SVMs with an appropriate transformed feature space to learn from. Nevertheless, both SVM and NSVM have their advantages and disadvantages. NSVM, for example, can be considered a complex model, with the additional requirement of fine tuning a neural network architecture for feature mappings. NSVM thus presents a large number of parameters and hyperparametrs that have to be adjusted by both the analyst and the model itself. SVM, on the other hand, has the distinct advantage of being easier to tune and train, yet, when using Bayesian algorithms for SVM hyperparameter optimization, training time can increase significantly (Table S13), while NSVMs still perform better on some datasets.

From the perspective of combining supervised and unsupervised learning approaches, the present study can be considered another example of how powerful data augmentation can be for improving classification model performance. Data augmentation is a very popular technique in computer vision, nevertheless, not all of these algorithms are readily applicable to numeric data of this type^48^. Here augmentation has been shown to not only improve the accuracy of most models (Tables 2, S8-S12 & S23-S28, Supplementary Appendix 7), but also improve the generalization capabilities of both SVMs and NSVMs^41,49^. This is mostly seen through the decrease in loss values across taxa (Tables S8-S12 & S23-S28), thus supporting observations made by Courtenay and González-Aguilera^41^ when applied to other GM datasets of palaeoanthropological and primatological origin. Similarly, the impact dataset imbalance has on algorithm performance is clearly evident, as seen through great drops in precision, recall and F values (Supplementary Appendix 6, Tables S8-S12 & S23-S28). In light of each of these observations, it can be seen how data augmentation can be a valuable tool for archaeological and palaeontological applications^41^, especially in cases where obtaining large sample sizes is difficult.

In the general context of new technologies applied to the field of taphonomy, it can be noted how the inclusion of some carnivore species within the samples have created notable statistical noise. This can be seen through drops in performance when increasing the number of target labels used in classification (e.g. the Pleistocene European Taxa dataset). From this perspective, it is important to point out that highly sophisticated techniques are not the all-encompassing solution that many analysts are looking for. When considering how carnivores can usually be described by the type^2,3,30^, ratio^27,34,36,50^ and size of bite damage^51–53^, alongside the location^54–56^ and extent of damage^57^, it can be seen how modelling carnivore behaviour should also take into account a wide range of different factors beyond BSMs. While neither one of these techniques can exclusively answer these questions, when combined, taphonomists currently have a very powerful toolkit at their disposal for discerning precise carnivore intervention. From a similar perspective, techniques in remote sensing, photogrammetry and microscopy also provide distinct advantages for the collection of different types of data, supported in many cases by the use of high resolution metric data^37,58–60^. Likewise, the use of computational learning has also proven a useful diagnostic tool for the analysis of fracture plane patterns^61^, obtaining high classification rates when applied to archaeological samples as well^62^. From another perspective, computer vision applications can also be considered an interesting development in the field of taphonomy^63^. In sum, and wherever possible, rather than commingling multiple species together into one large group, prior processes of elimination based on general taphonomic evidence should be performed in order to remove the least likely animals to have intervened. Algorithms will then be much easier to train, obtaining state of the art classification rates.

“Occam’s Razor” suggests that a more complex model is not always a better one. As seen here, without the use of large kernel machines, SVMs are equally likely to produce high level results. Likewise, while GANs are powerful non-parametric generative models, Bayesian inference is still a valuable tool for distribution modelling, as seen through better and faster performance in some of the reported cases. From this perspective, data science applications and AI can be considered both a very promising field of research, as well as a complex and challenging “pandoras box” of algorithms which analysts must take into consideration before planning a study. Nevertheless, and in combination with multiple other sources of data, advanced data science techniques can be considered a significant contribution to a taphonomist’s arsenal.

## Material and methods

### Sample

A total of 620 carnivore tooth pits were included in the present study. These samples included tooth marks produced by;

- Brown Bears (*Ursus arctos*, Ursidae, 69 pits)
- Spotted Hyenas (*Crocuta crocuta*, Hyaenidae, 86 pits)
- Wolves (*Canis lupus*, Canidae, 80 pits)
- African Wild Dogs (*Lycaon pictus*, Canidae, 89 pits)
- Foxes (*Vulpes vulpes*, Canidae, 53 pits)
- Jaguars (*Panthera onca*, Felidae, 77 pits)
- Leopards (*Panthera pardus*, Felidae, 84 pits)
- Lions (*Panthera leo*, Felidae, 82 pits)

Samples originated from a number of different sources, including animals kept in parks as well as wild animals. Samples obtained from wild animals included those produced by foxes as well as wolves. The only sample containing both wild and captive animals was the wolf sample. Preliminary data from these tooth pits revealed animals in captivity to have highly equivalent tooth pit morphologies to wild animals ($\vert d \vert$ = 0.125, *p* = 9.0e−14, BFB = 1.4e+11), while tooth scores revealed otherwise ($\vert d \vert$ = 0.152, *p* = 0.99, BFB = 3.7e+01 against $H_{a}$). Under this premise, and so as to avoid the influence of confounding variables that go beyond the scope of the present study, tooth scores were excluded from the present samples and are under current investigation (*data in preperation*). Nevertheless, other research have shown tooth pits to be more informative than tooth scores when considering morphology^20,23^.

When working with tooth mark morphologies, preference is usually given to marks found on long bone diaphyses. This is preferred considering how diaphyses are denser than epiphyses, and are thus more likely to survive during carnivore feeding. Nevertheless, when working with captive or semi-captive animals, controlling the bones that carnivores are fed is not always possible. This is due to the rules and regulations established by the institution where these animals are kept^64^. While this was not an issue for the majority of the animals used within the present study, in the case of *P. pardus*, animals were only fed ribs in articulation with other axial elements. In light of this, a careful evaluation on the effects this may have on the analogy of our samples was performed (Supplementary Appendix 2). These reflections concluded that in order to maintain a plausible analogy with tooth marks produced by other animals on diaphyses, tooth marks could only be used if found on the shaft of bovine ribs closest to the tuburcle, coinciding with the posterior and posterior-lateral portions of the rib, and farthest away from the costochondral junction^65^. This area of the rib corresponds to label RI3 described by Lam et al.^65^. Moreover, with a reported average cortical thickness of 2.3mm (± 0.13 mm) and Bone Mineral Density of $4490 kg/m^{3} [213.5, 334.6]$^66^, bovine ribs are frequently employed in most bone simulation experiments used in agricultural as well as general surgical sciences. Finally, considering the grease, muscle and fat content of typical domestic bovine individuals^67^, alongside the general size of *P. pardus* teeth, it was concluded that the use of rib elements for this sample was the closest possible analogy to the tooth marks collected from other animals.

Carnivores were fed a number of different sized animals, also dependent in most cases on the regulations established by the institution where these animals are kept^64^. Nevertheless, recent research has found statistical similarities between tooth marks found on different animals^25^, with the greatest differences occurring between large and small sized animals. Needless to say, considering the typical size of prey some of these carnivores typically consume, this factor was not considered of notable importance for the present study^25^ (Supplementary Appendix 1).

For the purpose of comparisons, animals were split into 5 groups according to ecosystem as well as taxonomic family. From an ecological perspective, two datasets were defined; (1) the Pleistocene European Taxa dataset containing *U. arctos*, *V. vulpes*, *C. crocuta*, *P. pardus*, *P. leo* and *C. lupus*; and (2) the African Taxa dataset containing *C. crocuta*, *P. pardus*, *L. pictus* and *P. leo*. When considering taxonomic groupings, animals were separated into 3 groups, including; (1) the Canidae dataset, including *V. vulpes*, *L. pictus* and *C. lupus*; (2) the Felidae dataset, including *P. pardus*, *P. onca* and *P. leo*; and (3) a general Taxonomic Family dataset, including all Canidae in the same group, all Felidae in the same group, followed by Hyaenidae and Ursidae. Some complementary details on each of these carnivores have been included in Supplementary Appendix 1.

All experiments involving carnivores were performed in accordance with the relevant ethical guidelines as set forth by park keepers and general park regulations. No animals were sacrificed specifically for the purpose of these experiments. Likewise, carnivores were not manipulated or handled at any point during the collection of samples. Collection of chewed bones were performed directly by park staff and assisted by one of the authors (JY). The present study followed the guidelines set forth by ARRIVE (https://arriveguidelines.org/) wherever necessary. No licenses or permits were required in order to perform these experiments. Finally, in the case of animals in parks, bone samples were provided by the park according to normal feeding protocols. More details can be consulted in the Extended Samples section of the supplementary files.

### 3D modelling and landmark digitisation

Digital reconstructions of tooth marks were performed using Structured Light Surface Scanning (SLSS)^68^. The equipment used in the present study was the DAVID SLS-2 Structured Light Surface Scanner located in the C.A.I. Archaeometry and Archaeological Analysis lab of the Complutense University of Madrid (Spain). This equipment consists of a DAVID USB CMOS Monochrome 2-Megapixel camera and ACER K11 LED projector. Both the camera and the projector were connected to a portable ASUS X550VX personal laptop (8 GB RAM, Intel^®^ Core^TM^ i5 6300HQ CPU (2.3 GHz), NVIDIA GTX 950 GPU) via USB and HDMI respectively. The DAVID’s Laser Scanner Professional Edition software is stored in a USB Flash Drive. Equipment were calibrated using a 15 mm markerboard, using additional macro lenses attached to both the projector and the camera in order to obtain optimal resolution at this scale. Once calibrated the DAVID SLS-2 produces a point cloud density of up to 1.2 million points which can be exported for further processing via external software.

The landmark configuration used for this study consists of a total of 30 landmarks (LMs)^21^; 5 fixed Type II landmarks^18^ and a $5 \times 5$ patch of semilandmarks^69^ (Fig. S2). Of the 5 fixed landmarks, LM1 and LM2 mark the maximal length (*l*) of each pit. For the correct orientation of the pit, LM1 can be considered to be the point along the maximum length furthest away from the perpendicular axis marking the maximum width (*w*). LM2 would therefore be the point closest to said perpendicular axis (see variables $d_{1}$ and $d_{2}$ in Fig. S2 for clarification). LM3 and LM4 mark the extremities of the perpendicular axis (*w*) with LM3 being the left-most extremity and LM4 being the right-most extremity. LM5 is the deepest point of the pit. The semilandmark patch is then positioned over the entirety of the pit, so as to capture the internal morphology of the mark.

Landmark collection was performed using the free Landmark Editor software (v.3.0.0.6.) by a single experienced analyst. Inter-analyst experiments prior to landmark collection revealed the landmark model to have a robustly defined human-induced margin of error of 0.14 ± 0.09 mm (Median ± Square Root of the Biweight Midvariance). Detailed explanations as well as an instructional video on how to place both landmarks and semilandmarks can be consulted in the Supplementary Appendix and main text of Courtenay et al.^21^.

### Geometric morphometrics

Once collected, landmarks were formatted as morphologika files and imported into the R free software environment (v.3.5.3, https://www.r-project.org/). Initial processing of these files consisted in the orthogonal tangent projection into a new normalized feature space. This process, frequently referred to as Generalized Procrustes Analysis (GPA), is a valuable tool that allows for the direct comparison of landmark configurations^18,19,70^. GPA utilises different superimposition procedures (translation, rotation and scaling) to quantify minute displacements of individual landmarks in space^71^. This in turn facilitates the comparison of landmark configurations, as well as hypothesis testing, using multivariate statistical analyses. Nevertheless, considering observations made by Courtenay et al.^20,21,25^ revealed tooth mark size to be an important conditioning factor in their morphology, prior analyses in allometry were also performed^72^. From this perspective, allometric analyses first considered the calculation of centroid sizes across all individuals; the square root of the sum of squared distances of all landmarks of an object from their centroid^18^. These calculations were then followed by multiple regressions to assess the significance of shape-size relationships. For regression, the logarithm of centroid sizes were used. In cases where shape-size relationships proved significant, final superimposition procedures were performed excluding the scaling step of GPA (*form*).

In addition to these analyses, preliminary tests were performed to check for the strength of phylogenetic signals^73^. This was used as a means of testing whether groups of carnivores produced similar tooth pits to other members of the same taxonomic family. For details on the phylogenies used during these tests, consult Fig. S1 and Supplementary Appendix 1.

For the visualisation of morphological trends and variations, Thin Plate Splines (TPS) and central morphological tendencies were calculated^19,71^. From each of these mean landmark configurations, for ease of pattern visualisation across so many landmarks, final calculations were performed using Delaunay 2.5D Triangulation algorithms^74^ creating visual meshes of these configurations in Python (v.3.7.4, https://www.python.org/).

Once normalised, landmark coordinates were processed using dimensionality reduction via Principal Components Analyses (PCA). In order to identify the optimal number of Principal Component Scores (PC Scores) that best represented morphological variance, permutation tests were performed calculating the observed variance explained by each PC with the permuted variance over 50 randomized iterations^75^. Multivariate Analysis of Variance (MANOVA) tests were then performed on these select PCs to assess the significance of multivariate morphological variance among samples.

Geometric Morphometric applications were programmed in the R programming language (Sup. Appendix 8).

### Robust statistics

While GPA is known to normalize data^76^, this does not always hold true. Under this premise, caution must be taken when performing statistical analyses on these datasets. Taking this into consideration, prior to all hypothesis testing, normality tests were also performed. These included Shapiro tests and the inspection of Quantile–Quantile graphs. In cases where normality was detected, univariate hypothesis tests were performed using traditional parametric Analysis of Variance (ANOVA). For multivariate tests, such as MANOVA, calculations were derived using the Hotelling-Lawley test-statistic. When normality was rejected, robust alternatives to each of these tests were chosen. In the case of univariate testing, the Kruskal–Wallis non-parametric rank test was prefered, while for MANOVA calculations, Wilk’s Lambda was used.

Finally, in light of some of the recommendations presented by The American Statistical Association (ASA), as debated in Volume 73, Issue Sup1 of *The American Statistician*^77,78^, the present study considers *p*-values of $>2\sigma$ from the mean to indicate only suggestive support for the alternative hypothesis ($H_{a}$). $p \; > \; 0.005$, or where possible, $3\sigma$ was therefore used as a threshold to conclude that $H_{a}$ is “significant”. In addition, Bayes Factor Bound (BFB) values (Eq. 1) have also been included alongside all corresponding *p*-Values^79^. Unless stated otherwise, BFBs are reported as the odds in favor of the alternative hypothesis (BFB:1). More details on BFB, Bayes Factors and the $p \; > \; 3\sigma$ threshold have been included in Supplementary Appendix 3. General BFB calibrations in accordance with Benjamin and Berger’s Recommendation 0.3^79^, as well as False Positive Risk values according to Colquhoun’s proposals^80^, have also been included in Table S20 of Supplementary Appendix 3.

1 $$\begin{aligned} BFB = \frac{1}{-e \; p \; \log (p)} \end{aligned}$$

All statistical applications were programmed in the R programming language (Sup. Appendix 8).

### Computational learning

Computational Learning employed in this study consisted of two main types of algorithm; Unsupervised and Supervised algorithms. The concept of “learning” in AI refers primarily to the creation of algorithms that are able to extract patterns from raw data (i.e. “learn”), based on their “experience” through the construction of mathematical functions^38,81^. The basis of all AI learning activities include the combination of multiple components, including; linear algebra, calculus, probability theory and statistics. From this, algorithms can create complex mathematical functions using many simpler concepts as building blocks^38^. Here we use the term “Computational Learning” to refer to a very large group of sub-disciplines and sub-sub-disciplines within AI. Deep Learning and Machine Learning are terms frequently used (and often debated), however, many more branches and types of learning exist. Under this premise, and so as to avoid complication, the present study has chosen to summarise these algorithms using the term “Computational”.

Similar to the concepts of Deep and Machine Learning, many different types of supervision exist. The terms supervised and unsupervised refer to the way raw data is fed into the algorithm. In most literature, data will be referred to via the algebraic symbol *x*, whether this be a vector, scalar or matrix. The objective of algorithms are to find patterns among a group of *x*. In an unsupervised context, *x* is directly fed into the algorithm without further explanation. Algorithms are then forced to search for patterns that best explain the data. In the case of supervised contexts, *x* is associated with a label or target usually denominated as *y*. Here the algorithm will try and find the best means of mapping *x* to *y*. From a statistical perspective, this can be explained as $p\left( y \vert x \right)$. In sum, unsupervised algorithms are typically used for clustering tasks, dimensionality reduction or anomaly detection, while supervised learning is typically associated with classification tasks or regression.

The workflow used in the present study begins with dimensionality reduction, as explained earlier with the use of PCA. While preliminary experiments were performed using non-linear dimensionality reduction algorithms, such as t-distributed Stochastic Neighbor Embedding (t-SNE)^82^ and Uniform Manifold Approximation and Projection (UMAP)^83^, PCA was found to be the most consistent across all datasets, a point which should be developed in detailed further research. Once dimensionality reduction had been performed, and prior to any advanced computational modelling, datasets were cleaned using unsupervised Isolation Forests (IFs)^84^. Once anomalies had been removed, data augmentation was performed using two different unsupervised approaches; Generative Adversarial Networks (GANs)^38–41^ and Markov Chain Monte Carlo (MCMC) sampling^44^. Data augmentation was performed for two primary reasons; (1) the simulation of larger datasets to ensure supervised algorithms have enough information to train from, and (2) to balance datasets so each sample has the same size. Both MCMCs and GANs were trialed and tested using robust statistics to evaluate quality of augmented data^41^. Once the best model had been determined, each of the datasets were augmented so they had a total sample size of $n = 100$. In the case of the Taxonomic Family dataset, augmentation was performed until all samples had the same size as the largest sample.

Once augmented, samples were used for the training of supervised classification models. Two classification models were tried and tested; Support Vector Machines (SVM)^85^ and Neural Support Vector Machines (NSVM)^86,87^. NSVMs are an extension of SVM using Neural Networks (NNs)^38^ as feature extractors, in substituting the kernel functions typically used in SVMs. Hyperparameter optimization for both SVMs and NSVMs were performed using Bayesian Optimization Algorithms (BOAs)^88^.

Supervised computational applications were performed in both the R and Python programming languages (Sup. Appendix 8). For full details on both unsupervised and supervised computational algorithms, consult the Extended Methods section of the Supplementary Materials.

Evaluation of supervised learning algorithms took into account a wide array of different popular evaluation metrics in machine and deep learning. These included; Accuracy, Sensitivity, Specificity, Precision, Recall, Area Under the receiver operator characteristic Curve (AUC), the F-Measure (also known as the F1 Score), Cohen’s Kappa ($\kappa$) statistic, and model Loss. Each of these metrics, with the exception of loss, are calculated using confusion matrices, measuring the ratio of correctly classified individuals (True Positive & True Negative) as well as miss-classified individuals (False Positive & False Negative). For more details see Supplementary Appendix 6.

Accuracy is simply reported as either a decimal $\left[ 0, 1\right]$ or a percentage. Accuracy is a metric often misinterpreted, as explained in Supplementary Appendix 6, and should always be considered in combination with other values, such as Sensitivity or Specificity. Both Sensitivity and Specificity are values reported as decimals $\left[ 0, 1\right]$, and are used to evaluate the proportion of correct classifications and miss-classifications. AUC values are derived from receiver operator characteristic curves, a method used to balance and graphically represent the rate of correctly and incorrectly classified individuals. The closest the curve gets to reaching the top left corner of the graph, the better the classifier, while diagonal lines in the graph represent a random classifier (poor model). In order to quantify the curvature of the graph, the area under the curve can be calculated (AUC), with $AUC=1$ being a perfect classifier and $AUC=0.5$ being a random classifier. The $\kappa$ statistic is a measure of observer reliability, usually employed to test the agreement between two systems. When applied to confusion matrix evaluations, $\kappa$ can be used to assess the probability that a model will produce an output $\hat{y}$ that coincides with the real output *y*. $\kappa$ values typically range between $\left[ 0, 1\right]$, with $\kappa =1$ meaning perfect agreement, $\kappa =0$ being random agreement, and $\kappa =0.8$ typically used as a threshold to define a near-perfect or perfect algorithm.

While in the authors’ opinion, AUC, Sensitivity and Specificity values are the most reliable evaluation metrics for studies of this type (Supp. Appendix 6), for ease of comparison with other papers or authors who choose to use other metrics, we have also included Precision, Recall and F-Measure values. Precision and Recall values play a similar role to sensitivity and specificity, with recall being equivalent to sensitivity, and precision being the calculation of the number of correct positive predictions made. Precision and Recall, however, differ from their counterparts in being more robust to imbalance in datasets. F-Measures are a combined evaluation of these two measures. For more details consult Supplementary Appendix 6.

Loss metrics were reported using the Mean Squared Error (Eq. 2);

2 $$\begin{aligned} MSE = \frac{1}{n} \sum _{i = 1}^{n} \left( y_{i} - \hat{y}_{i} \right) ^{2} \end{aligned}$$

Loss values are interpreted considering values closest to 0 as an indicator of greater confidence when using the model to make new predictions.

Final evaluation metrics were reported when using algorithms to classify only the original samples, without augmented data. Augmented data was, therefore, solely used for training and validation. Finally, so as to assess the impact data augmentation has on supervised learning algorithms, algorithms were also trained on the raw data. This was performed using 70% of the raw data for training, while the remaining 30% was used as a test set.

## Supplementary Information

Supplementary Information.

## Data Availability

All the relevant data and code used for the present study have been made readily available online via the corresponding author’s GitHub page: https://github.com/LACourtenay/Carnivore_Tooth_Pit_Classification. Any queries or issues regarding data or code should be directed to L.A. Courtenay (ladc1995@gmail.com).

## References

[1] Brain, C. K. *Hunters or the Hunted? An introduction to African cave taphonomy* (University of Chicago Press, 1981).

[2] Binford, L. R. *Bones: Ancient Men and Modern Myths* (Academic Press Inc., 1981).

[3] Blumenschine R (1995). Percussion marks, tooth marks and experimental determinations of the timing of hominid and carnivore access to long bones at FLK Zinjanthropus, Olduvai Gorge, Tanzania. J. Hum. Evol..

[4] Domínguez-Rodrigo, M., Barba, R. & Egeland, C. P. *Deconstructing Olduvai* (Springer, 2007).

[5] Andrews P, Fernandez-Jalvo Y (1997). Surface modifications of the Sima de los Huesos fossil humans. J. Hum. Evol..

[6] Cueto M, Camarós E, Castaños P, Ontañón R, Arias P (2016). Under the skin of a lion: unique evidence of Upper Paleolithic exploitation and use of cave lion (*Panthera spelaea*) from the Lower Gallery of La Garma (Spain). PLoS ONE.

[7] Serangeli J, Kolfschoten TV, Starkovich BM, Conard NJ (2015). The European saber-tooth cat (*Homotehrium latidens*) found in the “Spear Horizon” at Schöningen (Germany). J. Hum. Evol..

[8] Aramendi J (2019). Who ate OH80 (Olduvai Gorge, Tanzania)? A geometric morphometric analysis of surface bone modifications of a Paranthropus boisei skeleton. Quatern. Int..

[9] Daujeard C (2016). Plesitocene hominins as a resource for carnivores: a c. 500,000-year-old human femur bearing tooth-marks in North Africa (Thomas Quarry I, Morocco). PLoS ONE.

[10] Starkovich BM, Conard NJ (2015). Bone taphonomy of the Schöningen “Spear Horizon South” and its implications for site formation and hominin meat provisioning. J. Hum. Evol..

[11] Boaz NT, Ciochon RL, Xu Q, Liu J (2004). Mapping and taphonomic analysis of the Homo erectus loci at Locality 1 Zhoukoudian, China. J. Hum. Evol..

[12] D’Errico F, Villa P, Pinto Llona A. C, Idarraga R. R (1998). A middle palaeolithic origin of music? Using cave-bear bone accumulations to assess the Divje Babe I bone “flute”. Antiquity.

[13] Diedrich CG (2015). “Neanderthal bone flutes”: simply products of Ice Age spotted hyena scavenging activities on cave bear cubs in European cave bear dens. R. Soc. Open Sci..

[14] Arsuaga JL (1997). Sima de los Huesos (Sierra de Atapuerca, Spain). The site. J. Hum. Evol..

[15] Dirks PN (2015). Geological and taphonomic context from the new hominin species Homo naledi from the Dinaledi Chamber, South Africa. eLife.

[16] Egeland CP, Domínguez-Rodrigo M, Pickering TR, Menter CG, Heaton JL (2018). Hominin skeletal part abundances and claims of deliberate disposal of corpses in the Middle Pleistocene. Proc. Natl. Acad. Sci..

[17] Domínguez-Rodrigo M (2017). Use and abuse of cut mark analyses: the Rorsach effect. J. Archaeol. Sci..

[18] Dryden, I. & Mardia, K. *Statistical Shape Analysis* (Wiley, 1998).

[19] Bookstein, F. L. *Morphometric Tools for Landmark Data* (Cambridge University Press, 1991).

[20] Courtenay LA (2019). Combining machine learning algorithms and geometric morphometrics: a study of carnivore tooth marks. Palaeogeogr. Palaeoclimatol. Palaeoecol..

[21] Courtenay LA (2020). Obtaining new resolutions in carnivore tooth pit morphological analyses: a methodological update for digital taphonomy. PLoS ONE.

[22] Yravedra J (2017). The use of micro-photogrammetry and geometric morphometrics for identifying carnivore agency in bone assemblages. J. Archaeol. Sci. Rep..

[23] Yravedra J, Maté-González MÁ, Courtenay LA, González-Aguilera D, Fernández-Fernández M (2019). The use of canid tooth marks on bone for the identification of livestock predation. Sci. Rep..

[24] Aramendi J (2017). Discerning carnivore agency through the three-dimensional study of tooth pits: Revisiting crocodile feeding behaviour at FLK-Zinj and FLK NN3 (Olduvai Gorge, Tanzania). Palaeogeogr. Palaeoclimatol. Palaeoecol..

[25] Courtenay LA (2020). The effects of prey size on carnivore tooth mark morphologies on bone; the case study of Canis lupus signatus. Hist. Biol..

[26] Marean CW, Kim SY (1998). Mousterian large-mammal remains from Kobeh Cave. Curr. Anthropol..

[27] Arriaza MC, Domínguez-Rodrigo M, Yravedra J, Baquedano E (2016). Lions as bone accumulators? Palaeontological and ecological implications of a modern bone assemblage from Olduvai Gorge. PLoS ONE.

[28] Gidna AO, Kusui B, Mabulla A, Musiba C, Domínguez-Rodrigo M (2014). An ecological neo-taphonomic study of carcass consumption by lions in Tarangire National Park (Tanzania) and its relevance for human evolutionary biology. Quatern. Int..

[29] Pickering TR, Heaton JL, Zwodeski SE, Kuman K (2011). Taphonomy of bones from baboons killed and eaten by wild leaopards in Mapungubwe National Park, South Africa. J. Taphon..

[30] Haynes G (1983). A guide for differentiating mammalian carnivore taxa responsible for gnaw damage to herbivore limb bones. Paleobiology.

[31] Yravedra J, Lagos L, Bárcena F (2011). A taphonomic study of wild wolf Canis lupus modifications of horse bones in Northwestern Spain. J. Taphon..

[32] Yravedra J, Andrés M, Domínguez-Rodrigo M (2014). A taphonomic study of the African wild dog (*Lycaon pictus*). Archaeol. Anthropol. Sci..

[33] Yravedra J, Andrés M, Fosse P, Besson JP (2014). Taphonomic analysis of small ungulates modified by fox (*Vulpes vulpes*) in Southwestern Europe. J. Taphom..

[34] Rodríguez-Alba JJ, Linares-Matás G, Yravedra J (2019). First assessments of the taphonomic behaviour of jaguar (*Panthera onca*). Quatern. Int..

[35] Saladié P, Huguet R, Díez C, Rodríguez-Hidalgo A, Carbonell E (2013). Taphonomic modifications produced by modern brown bears (*Ursus arctos*). Int. J. Osteoarchaeol..

[36] Gidna A, Yravedra J, Domínguez-Rodrigo M (2013). A cautionary note on the use of captive carnivores to model wild predator behavior: a comparison of bone modification patterns on long bones by captive and wild lions. J. Archaeol. Sci..

[37] Courtenay LA, Huguet R, González-Aguilera D, Yravedra J (2020). A hybrid geometric morphometric deep learning approach for cut and trampling mark classification. Appl. Sci..

[38] Goodfellow, I., Bengio, Y. & Courville, A. *Deep Learning* (MIT Press, 2016).

[39] Goodfellow, I. *et al.* Generative adversarial nets. In *Proc. Int. Conf. Neur. Inf. Process. Syst.* 2672–2680. arXiv:1406.2661v1 (2014).

[40] Lucic, M., Kurasch, K., Michalski, M., Bousquet, O. & Gelly, S. Are GANs created equal? A large scale study. In *Proc. Int. Conf. Neur. Inf. Process. Syst.* 698–707. arXiv:1406.2661v1 (2018).

[41] Courtenay LA, González-Aguilera D (2020). Geometric morphometric data augmentation using generative computational learning algorithms. Appl. Sci..

[42] Metropolis N, Rosenbluth A, Teller A, Teller E (1953). Equations of state calculations by fast computing machines. J. Chem. Phys..

[43] Hastings W (1970). Monte Carlo sampling methods using Markov chains and their application. Biometrika.

[44] Gamerman, D. & Lopes, H. F. *Markov Chain Monte Carlo* (Chapman & Hall, 2006).

[45] Martin, O. *Bayesian Analysis with Python* (Packt, 2018).

[46] Höhle J, Höhle M (2009). Accuracy assessment of digital elevation models by means of robust statistical methods. ISPRS J. Photogram. Remote Sens..

[47] Rodríguez-Martín M, Rodríguez-Gonzálvez P, Ruiz de Oña Crespo E, González-Aguilera D (2019). Validation of portable mobile mapping system for inspection tasks in thermal and fluid-mechanical facilities. Remote Sens..

[48] Shorten C, Khoshgoftaar TM (2019). A survey on image data augmentation for deep learning. J. Big Data.

[49] Such, F. P., Rawal, A., Lehman, J., Stanley, K. O. & Clune, J. Generative teaching networks: accelerating neural architecture search by learning to generate synthetic training data. *Uber AI Labs*. arXiv:1912.07768v1 (2019).

[50] Domínguez-Rodrigo M, Gidna AO, Yravedra J, Musiba C (2012). A comparative neo-taphonomic study of felids, hyaenids and canids: an analogical framework based on long bone modification patterns. J. Taphon..

[51] Andrés M, Gidna AO, Yravedra J, Domínguez-Rodrigo M (2012). A study of dimensional differences of tooth marks (pits and scores) on bones modified by small and large carnivores. Archaeol. Anthropol. Sci..

[52] Domínguez-Rodrigo M, Piqueras A (2003). The use of tooth pits to identify carnivore taxa in tooth-marked archaeofaunas and their relevance to reconstruct hominid carcass processing behaviours. J. Archaeol. Sci..

[53] Selvaggio MM, Wilder J (2001). Identifying the involvement of multiple carnivore taxa with archaeological bone assemblages. J. Archaeol. Sci..

[54] Parkinson J, Plummer T, Hartstone-Rose A (2015). Characterizing felid tooth marking and gross bone damage patterns using GIS image analysis: an experimental feeding study with large felids. J. Hum. Evol..

[55] Pobiner B, Dumouchel L, Parkinson J (2020). A new semi-quantitative method for coding carnivore chewing damage with an application to modern African lion-damaged bones. Palaios.

[56] Domínguez-Rodrigo M (2021). A 3D taphonomic model of long bone modification by lions in medium-sized ungulate carcasses. Sci. Rep..

[57] Domínguez-Rodrigo M (2015). A new methodological approach to the taphonomic study of paleontological and archaeological faunal assemblages: a preliminary case study from Olduvai Gorge (Tanzania). J. Archaeol. Sci..

[58] Pante M (2017). A new high-resolution 3-D quantitative method for identifying bone surface modifications with implications for the Early Stone Age archaeological record. J. Hum. Evol..

[59] Bello SM, Soligo C (2008). A new method for the quantitative analysis of cutmark micromorphology. J. Archaeol. Sci..

[60] Duches R (2020). Experimental and archaeological data for the identification of projectile impact marks on small-sized mammals. Sci. Rep..

[61] Moclán A, Domínguez-Rodrigo M, Yravedra J (2019). Classifying agency in bone breakage: an experimental analysis of fracture planes to differentiate between hominin and carnivore dynamic and static loading using machine learning (ML) algorithms. Archaeol. Anthropol. Sci..

[62] Moclán A (2020). Identifying the bone-breaker at the Navalmaíllo Rock Shelter (Pinilla del Valle, Madrid) using machine learning algorithms. Archaeol. Anthropol. Sci..

[63] Jiménez-García B, Abellán N, Baquedano E, Cifuentes-Alcobendas G, Domínguez-Rodrigo M (2020). Corrigendum to “deep learning improves taphonomic resolution: high accuracy in differentiating tooth marks made by lions and jaguars”. J. R. Soc. Interface.

[64] Fidgett, A. L. & Plowman, A. Nutrition and diet evaluation. In Bishop, J., Hosey, G. & Plowman, A. (eds.) *Handbook of Zoo & Aquarium Research*, 154–175 (BIAZA, 2013).

[65] Lam YM, Chen X, Pearson OM (1999). Intertaxonomic variability in patterns of bone density and the differential representation of Bovid, Cervid and Equid elements in the archaeological record. Am. Antiq..

[66] Szalma J (2019). The influence of the chosen in vitro bone simulation model on intraosseous temperatures and drilling times. Sci. Rep..

[67] Johnson ER, Chant DC (1998). Use of carcass density for determining carcass composition in beef cattle. N. Zeal. J. Agric. Res..

[68] Maté-González MÁ, Aramendi J, Yravedra J, González-Aguilera D (2017). Statistical comparison between low-cost methods for 3D characterization of cut-marks on bones. Remote Sens..

[69] Gunz, P., Mitteroecker, P. & Bookstein, F. L. Semilandmarks in three dimensions. In *Modern Morphometrics in Physical Anthropology* (ed. Slice, D. E.) 73–98 (Plenum Publishers, 2005).

[70] Klingenberg C, Monteiro L (2005). Distances and directions in multidimensional shape spaces: implications for morphometric applications. Soc. Syst. Biol..

[71] Bookstein F (1989). Principal warps: thin plate spline and the decomposition of deformations. IEEE Trans. Pattern Anal. Mach. Intell..

[72] Adams DC, Rohlf FJ, Slice DE (2013). A field comes of age: geometric morphometrics in the 21st century. Hystrix.

[73] Klingenberg CP, Gidaszewski NA (2010). Testing and quantifying phylogenetic signals and homoplasy in morphometric data. Syst. Biol..

[74] Delaunay B (1934). Sur la sphère vide. Bull. l’Acad. Sci. l’URSS Classe des Sci. Math. Nat..

[75] Viñuela A (2020). Genetic variant effects on gene expression in human pancreatic islets and their implications for T2D. Nat. Commun..

[76] Diaconsis P, Freedman D (1984). Asymptotics of graphical projection of pursuit. Ann. Stat..

[77] Wasserstein RL, Schirm AL, Lazar NA (2019). Moving to a world beyond “*p*$$<$$ 0.05”.. Am. Stat..

[78] Wasserstein RL, Lazar NA (2016). The ASA statement on p-values: context, process, and purpose. Am. Stat..

[79] Benjamin DJ, Berger JO (2019). Three recommendations for improving the use of p-values. Am. Stat..

[80] Colquhoun D (2019). The false positive risk: a proposal concerning what to do about p-values. Am. Stat..

[81] Bishop, C. *Pattern Recognition and Machine Learning* (Springer, 2006).

[82] Hinton, G. E. & Roweis, S. T. Stochastic neighbor embedding. In *Advances in Neural Information Processing Systems.* **857–864** (2003).

[83] McInnes L, Healy J, Melville J (2018). UMAP: uniform manifold approximation and projection. J. Open Source Softw..

[84] Liu, F. T., Ting, K. M. & Zhou, Z. H. Isolation forest. In *2008 Eighth IEEE International Conference on Data Mining*, 413–422. 10.1109/ICDM.2008.17 (2008).

[85] Cortes C, Vapnik V (1995). Support-vector networks. Mach. Learn.

[86] Wiering, M. A. *et al.* The neural support vector machine. In *The 25th Benelux Artificial Intelligence Conference*, 257–254 (2013).

[87] Rahimi A, Recht B (2007). Random features for large-scale kernel machines. Proc. Int. Conf. Neural Inf. Process. Syst..

[88] Bergstra J, Bardenet R, Bengio Y, Kégl B (2011). Algorithms for hyper-parameter optimization. Proc. Int. Conf. Neural Inf. Process. Syst..

