# Supplementary material for: Developments in data science solutions for carnivore tooth pit classification
Source: Sci Rep. 2021 May 13;11:10209. doi: 10.1038/s41598-021-89518-4 (PMC8119709; doi:10.1038/s41598-021-89518-4)
Supplement: Supplementary file 1 — Supplementary Information. [file 41598_2021_89518_MOESM1_ESM.pdf]

Developments in Data Science Solutions for Carnivore Tooth Pit Classification: Supplementary Materials

Lloyd A. Courtenay[1,*], Darío Herranz-Rodrigo[2,3], Diego González-Aguilera[1], and José Yravedra[2,3]

[1]Department of Cartographic and Terrain Engineering, Higher Polytechnic School of Ávila, University of Salamanca, Hornos Caleros 50, 05003, Ávila, Spain
[2]Department of Prehistory, Complutense University, Prof. Aranguren s/n, 28040, Madrid, Spain
[3]C. A. I. Archaeometry and Archaeological Analysis, Complutense University, Professor Aranguren 2/n, 28040, Madrid, Spain
[*]Corresponding Author Email: ladc1995@gmail.com

Contents

Extended Samples 2
Extended Methods 3
Computational Learning . . . 3
Unsupervised Learning . . . 3
Supervised Learning . . . 4
Supplementary Tables and Figures 6
Supplementary Tables . . . 6
Supplementary Figures . . . 10
Supplementary Appendix 1 : Carnivores Studied 11
Supplementary Appendix 2 : Rib Cortical Density 14
Supplementary Appendix 3 : *p*-Values, Bayes Factor Bounds & False Positive Risks 16
A Brief Overview of p-Value Historiography . . . 17
p-Value Calibrations and the use of Bayes Theorem . . . 18
Supplementary Appendix 4 : Central Limit Theorem 22
CLT Theory . . . 22
Present Application . . . 23
Supplementary Appendix 5 : Neural Network Activation Functions 24
Rectified Linear Units (ReLU) . . . 25
Self-Gated Rectified Activation Functions: Swish . . . 26
Supplementary Appendix 6 : Computational Learning Evaluation Metrics 27
Supplementary Appendix 7 : Computational Learning on Non-Augmented Datasets 29
Supplementary Appendix 8 : Software 32
Geometric Morphometrics . . . 32
Statistical Applications . . . 32
Unsupervised Computational Learning . . . 32
Supervised Computational Learning . . . 32
Miscellaneous: Data handling and visualisation . . . 32
References 33

Extended Samples

The samples used for the present study originates from multiple sources, all of which were collected and handled by one of the authors (JY). With the objective of providing the maximum level of transparency possible, the present supplementary section has provided a number of details regarding the nature of these samples, alongside a list of publications that have used them.

All bite damage samples obtained were found on the diaphyses of appendicular long bones. The only exception to this is the *Panthera pardus* samples (see main text and Supplementary Appendix 2). One all bones had been collected, they were cleaned for 12 hours in boiling water. So as to avoid any possible damage to the tooth marks, this boiling process was performed solely using water, without the use of any added chemicals.

Samples included;

- Brown Bears (*Ursus arctos*, Ursidae, 69 pits). This sample was obtained from bears living in the Cabárceno natural park, collected during the months of June and July, 2020. All marks were found on the diaphyses of horse long bones. All tooth marks were produced by multiple adult brown bears of varying sexes. Bones were collected a couple of days after the initial feeding. The present study is the first to use this sample.

- Spotted Hyenas (*Crocuta crocuta*, Hyaenidae, 86 pits). This sample was obtained from hyenas living in the Cabárceno natural park, collected during the year 2012. All marks were found on the diaphyses of horse tibiae and radii. All tooth marks were produced by a single female adult hyena. Bones were exposed to hyenas for only a few hours, considering how bones left for longer were typically completely consumed. This sample was originally published in the work of Domínguez-Rodrigo and colleagues (2015)[1]. This sample, however, has also been included in a large number of other studies, including analyses presented by Aramendi et al. (2017, 2019)[2,3], Arriaza et al. (2017, 2019)[4,5], Yravedra et al. (2017)[6], Courtenay et al. (2019)[7], and Abellán et al. (2021)[8].

- Wolves (*Canis lupus*, Canidae, 80 pits). This sample was obtained from a mixture of wild wolves and others living in parks. Four main samples were used [*]; the first two samples consisting of wild wolves from the areas of Flechas and Villardeciervos (Zamora, Castilla y León), while a third sample was obtained from the Cabárceno natural park, and a final sample from the Hosquillo natural park. The original tooth marks from these 4 samples amounted to a total of 283 digitised tooth pits and 288 tooth scores. So as to avoid subjective bias induced by manually selecting 3d models from these 571 tooth marks, an algorithm was used to randomly select 80 marks. The number 80 was chosen primarily to match the relative sample sizes obtained from other animals. Across all four samples, marks were found on the diaphyses of horse, sheep, deer, ibex, boar and cow appendicular elements. Difference in animal size has previously been revealed to not be an important conditional factor on these types of statistical analyses[10]. All tooth marks were produced by a group of adult wolves, with varying pack sizes (>5) and different sexes. In the case of the Hosquillo Cabárceno samples, bones were collected between a number of days to a number of weeks after initial feedings. A large number of studies have published data regarding these samples. The Cabárceno sample was originally publisehd by Yravedra et al. (2014, 2017, 2019)[6,11,12], and has subsequently been used by Aramendi et al. (2017)[3], Courtenay et al. (2019, 2020)[7,10,13], and Abellán et al. (2021)[8]. The Hosquillo sample has been published by Moclán et al. (2019)[14] and Courtenay et al. (2020)[10]. Both the Villardeciervos and Flechas samples are yet to be published in detail (*in prep*).

- African Wild Dogs (*Lycaon pictus*, Canidae, 89 pits). This sample was obtained from African wild dogs living in the Cabárceno natural park, collected during the year 2010. All marks were found on the diaphyses of horse radii and tibiae. All tooth marks were produced by a pair of African wild dogs of both sexes. Bones were collected a couple of days after the initial feeding. This sample was originally published in the work of Yravedra et al. (2014)[12].

- Foxes (*Vulpes vulpes*, Canidae, 53 pits). This sample was obtained from wild adult foxes in the area of Ayllón (Segovia), collected during the year 2002. All tooth marks were produced on the diaphyses of sheep long bones. This sample was originally published in the work of Yravedra et al. (2014)[15], however has also been used in Yravedra et al. (2019)[11].

- Jaguars (*Panthera onca*, Felidae, 77 pits). This sample was obtained from jaguars living in the Cabárceno natural park, collected during the years 2009 and 2010. All marks were found on the diaphyses of horse tibiae and radii. All tooth marks were produced by multiple jaguars of varying sexes. Bones were collected a couple of days after the initial feeding, however include feeding sessions that span over a number of months. This sample was originally published in the work of Domínguez-Rodrigo and colleagues (2015)[1]. This sample, however, has also been included in a large number of other studies, including analyses presented by Aramendi et al. (2017, 2019)[2,3], Yravedra et al. (2017)[6], Courtenay et al.

[*] One of the authors (J.Y.) would like to point out that while an additional wolf sample is at our disposition, originating from wild wolves in Monte Campelo (Lugo, Spain), said sample has only ever been used for one study, originally published by J.Y. in 2011[9]. For numerous reasons these samples have not been used since.

(2019)[7], Rodríguez-Alba et al. (2019)[16], Jiménez-García et al. (2020)[17], and Abellán et al. (2021)[8]. The most detailed description of this sample can be consulted in Rodríguez-Alba et al. (2019)[16].

- Leopards (*Panthera pardus*, Felidae, 84 pits). This sample was obtained from Sri Lankan leopards (subspecies *P. p. kotiya*) living in the Biopark of Fuengirola (Málaga), collected during the autumn of 2020. This is the only sample of the present study to include cow axial skeletal elements. All tooth marks were produced by a pair of leopards of varying sexes. Bones were collected a couple of days after the initial feeding. The present study is the first to use this sample.

- Lions (*Panthera leo*, Felidae, 82 pits). This sample was obtained from lions living in the Cabárceno natural park, collected during the years 2011. All marks were found on the diaphyses of horse radii and tibiae. All tooth marks were produced by a number of adult lion of varying sexes. Bones were collected a couple of days after the initial feeding. This sample was originally published by Domínguez-Rodrigo et al. (2012)[18] and Gidna et al. (2013)[19]. This sample, however, has also been included in a number of other studies, including analyses presented by Domínguez-Rodrigo et al. (2015, 2020)[1,20], Aramendi et al. (2017, 2019)[2,3], Arriaza et al. (2017, 2019)[4,5], Yravedra et al. (2017)[6], Courtenay et al. (2019)[7], Jiménez-García et al. (2020)[17], and Abellán et al. (2021)[8].

Extended Methods

Computational Learning

Unsupervised Learning

Beyond PCA, a total of 8 algorithms with 11 variants were used for the purpose of advanced data science applications. These included both supervised and unsupervised algorithms. Prior to any advanced computational modelling, datasets were cleaned using unsupervised Isolation Forests (IFs)[21]. IFs are a development of recursive partitioning trees, using the term *isolation* to refer to the 'separation of instances that are "few" and "different" from the rest of the instances'[21]. IFs thus consider "isolated" instances as data points that are located farthest from the root of the tree. From here anomaly scores can then be computed with scores $\gg 0.5$ considered as outliers.

After outlier removal, datasets were subjected to data augmentation. Two different approaches were considered; Generative Adversarial Networks (GANs)[22–25] and Markov Chain Monte Carlo (MCMC) sampling[26–29].

GANs are unsupervised algorithms that consist of two different neural networks; a Discriminator (D) and a generator (G)[22,23]. G is used to produce synthetic information while D evaluates G's output for authenticity. The two are trained simultaneously in a "zero-sum game"[23], with the intention of producing a generator capable of producing synthetic data that D is unable to tell apart from the original dataset[22,23]. G creates this data by sampling from a hypothetical distribution (e.g. $\mathcal{N}(\mu = 0, \sigma = 1)$) in the form of a fixed-length *latent* vector, known as a *latent vector*. The neural network is then trained to find the best means of mapping the hypothetical distribution onto the real sample domain. The output of G is then fed as input into D in an attempt to predict class labels for the generated points (real (1) or fake (0)).

For GANs, three different loss functions were considered[24]; the Least Square loss function (LSGAN)[30], Wasserstein loss (WGAN)[31] and a variant of WGAN using Gradient Penalties (WGAN-GP)[32,33]. Models were trained for 1000 epochs using an $\mathbb{R}^{50}$ Latent Vector as input. For more details on the models used and their architectures consult Courtenay and González-Aguilera[25].

MCMCs are a family of algorithms that are typically used as inference engines in probabilistic programming[29]. MCMCs, as their name suggest, combine two fundamental data science concepts including Markovian Chains and Monte Carlo simulations. Markov Chains are stochastic models that can be used to explore the entirety of a distribution via "random walks", whereby the chain makes predictions on the position of future states based solely on the position of its present state, as well as the probability of transition to the next[28,29]. The Monte Carlo element of MCMC involves the simulation of drawn samples from the probability distribution, generating ensembles of Markov Chains that search for areas of high information density[28]. While many types of MCMCs exist, the present study used the Metropolis-Hastings 1st Order variant of MCMC[26,27]. The Metropolis-Hastings algorithm draws samples from a probability distribution $p(x)$ based on the distribution's density. Samples are calculated iteratively, with the next state of the Markov Chain being determined by calculating its suitability using the Metropolis-Hastings criterion[29]. If the suggested next state does not agree with the proposed probability of being accepted, then the current state is maintained and the process begins again.

MCMCs were used to generate samples for each of the sample distributions, thus generating new synthetic data points that are statistically likely to belong to the same distribution. While a number of techniques exist to define the distribution to be sampled from (θ)[29], the present study strictly defined $p(\theta|x)$ using robust statistical techniques. These considered robustly defined Gaussian priors, as supported by the *Central Limit Theorem* (See Supplementary Appendix 4), as well as skewed distributions[34,35]. In order to ensure the adaptability of the theoretical Gaussian to the empirical non-Gaussian distributions, robust metrics were used to define the central tendency and variance of these theoretical models (eq. S2). This included the

calculation of sample medians ($\tilde{x}$) and the Normalized Median Absolute Deviation (NMAD) (eq. S3)[36]. Skewed distributions, on the other hand, were defined in a similiar manner, while including an additional conditioning variable α to try and simulate the asymmetry observed in the original samples.

$$\mathcal{N}(\mu,\sigma) = p(x|\mu,\sigma) = \frac{1}{\sigma\sqrt{2\pi}} e^{-\frac{(x-\mu)^2}{2\sigma^2}} \tag{1}$$

$$\theta \sim \mathcal{N}(\mu = \tilde{x}, \sigma = NMAD) \tag{2}$$

$$NMAD = 1.4826 \cdot \frac{1}{n} \sum_{i=1}^{n} |x_i - \tilde{x}| \tag{3}$$

Once the target θ had been defined, MCMCs were trained for 10,000 to 30,000 iterations, discarding the first 2,000 iterations as the "burn-in period". Evaluation of MCMCs were performed considering chain autocorrelation performance as well as the Effective Sample Size (ESS) metric[29,37,38]. Autocorrelation is defined when sampled values are seen to be dependent on the sampled values at other iterations, whereas ESS is calculated as the difference of mean values divided by pooled standard deviation. An ideal autocorrelation value for MCMC ≈ 0, while ESS values $[1000, 10000]$ are recommended. For Metropolis-Hastings proposal mechanisms, a number of different hyperparameters were also tested. These mainly focused on defining the optimal σ values for the symmetrical proposal distribution. Each of these tests considered $\sigma = [0.1, 3)$, with $\sigma = 0.3$ producing the best results.

Final evaluation of both GANs and MCMCs was performed using robust statistical approaches, as described by Courenay and González-Aguilera[25]. First both GANs and MCMCs were used to generate synthetic samples of the exact same sample size as the original samples. This was performed to ensure that the samples being compared (synthetic and original) were balanced so as to not condition hypothesis testing results according to Cohen's d. Both samples were then subjected to a Two One-Sided equivalency Test (TOST), for the evaluation of the magnitude of similarities via Superior (εS or ΔS) and Inferior (εI or ΔI) equivalence bound parameters. Depending on sample homogeneity, the traditional Welch's t-statistic or trimmed non-parametric Yuen's robust t-statistic was used[39,40]. Once the most realistic synthetic-data generator had been established, the final model was used to augment all samples to $n = 100$ while ensuring that no repeated values were present.

Unsupervised computational applications were performed in both the R and Python programming languages (Sup. Appendix 8).

Supervised Learning

Two different supervised learning algorithms were considered for the present study; Support Vector Machines (SVM) and an extension of SVMs using Neural Networks (NSVM).

SVMs are a highly powerful modeling algorithm, useful for both regression and discriminant analyses. Unlike typical discriminant functions, SVMs use soft maximized-margins with an associated cost (c) hyperparameter to define decision boundaries that are less conditioned by the presence of outliers. The larger the c, the greater the softness of the margin. From this perspective, SVMs are less likely to overfit and can be considered more flexible[41]. SVMs additionally have the distinct advantage of using a *kernel trick* to overcome traditional limitations imposed by linearity[42]. From this perspective, SVMs use a defined function to project data-points onto a non-linear high dimensional feature space where the consequent decision boundary, or hyperplane, can be constructed[41,42]. A number of kernel functions exist for SVMs. The present study primarily considered the Radial Basis Function (RBF; eq. S4), as well as the Polynomial function, of which the former obtained the best results on all accounts. The radial kernel function has 1 main hyperparameter (γ), which scales the influence two points (x and x') will have during the kernel's re-projection of data.

$$K(x, x') = e^{-\gamma \|x - x'\|^2} \tag{4}$$

The Neural SVM (NSVM) is a concept originally proposed as a means of adding "depth" to SVMs[43], while using non-linear Neural Network architectures as a means of training a specified kernel function directly on the dataset. The added use of Neural Networks (NNs) thus add a highly versatile and flexible "kernel" based on the combined use of multiple layers of varying neuron densities for feature extraction. For NSVM, the present study additionally uses a single Random Fourier Feature (RFF) layer to define a complicated function on our original domain using Fourier Feature mappings[44–46]. In this context, RFF layers

project datapoints randomly onto a line, and then transform this information into a new $\mathbb{R}^n$ feature space using a specified shift-invariant kernel function[46]. Two different initialization functions were considered for RFF; a Gaussian transformation kernel and a Laplacian transformation kernel (eq. S5). The latter produced the best results. Beyond the kernel initialization, RFF layers require two additional functions; (1) the number of dimensions data will be mapped out onto and (2) a scale value α with similiar properties to γ from eq. S4.

$$K\left(x,x'\right) = e^{\frac{-|x-y|}{\alpha}} \tag{5}$$

The present system thus used a Laplacian RFF map as the first layer of the NSVM, followed by a series of hidden NN layers. For NN hidden layer design, numerous experiments were performed using different architectures, experimenting with a wide range of different parameters including different numbers of layers and layer densities, dropout layers, kernel initializers, batch normalization, weight regularizers, weight constraints[22]. The final architecture obtained consisted of;

- Laplacian RFF Layer
- Batch Normalization
- Dropout layer
- Hidden Layer with 250 Neurons, Random-Normal kernel initialization and L_2 regularization.
- Batch Normalization
- Dropout layer
- Hidden Layer with 250 Neurons, Random-Normal kernel initialization and L_2 regularization.
- Batch Normalization
- Dropout layer

For neuron activation, two primary activation functions were considered, tried and tested. These included the traditional Rectified Linear Unit (ReLU) and a self-gated variant known as Swish[47] (eq. S6). After careful consideration, swish was chosen as the optimal activation function for the present study. Firstly, swish has been noted to preserve and improve gradient flow, facilitating the training of deep neural networks. More details on this, however, can be consulted in Supplementary Appendix 5. Secondly, it was considered best to avoid the creation of very sparse vectors in the final layers of the NN, considering how the final neuron layers will be used as input into the SVM portion of the NSVM. For the final layer, initial training of the NN component of the NSVM was performed using the softmax activation function, where K is the number of target labels (eq. S7).

$$f(x) = x \cdot \frac{1}{1+e^{-x}} \tag{6}$$

$$f(x) = \frac{e^{x_i}}{\sum_{j=1}^{K} e^{x_j}} \tag{7}$$

Once defined, NNs were trained using x32 batches for 1000 epochs. The loss function used for initial training was categorical crossentropy, using categorical accuracy as an initial evaluation metric. NNs were trained using the adam optimization algorithm[48] with *Triangular2* cyclic learning rate (Base α = 0.0001, Max α = 0.01, Step Size = 16)[49]. A training-validation split of 80:20% was additionally used.

After training, the final softmax layer of the NN was removed and replaced with a multi-class Linear SVM for activation. The SVM proportion of NSVM was trained using k-fold cross-validation (k = 10).

Hyperparameter optimization for both SVM and all components of NSVM were performed using Bayesian Optimization Algorithms (BOA)[50–52]. For NSVM, BOA was used to define a large series of different parameters in the NN, including; optimal dropout rate (0.5), number of neurons (250), L_2 parameters (0.0001), as well as the output dimensions (500) and α (0.26) for RFS. These parameters proved efficient for all datasets, regardless of the complexity or number of target labels. BOA was then used to fine tune the optimal c for the SVM part of NSVM as well as the c and γ values in Radial SVM. For both c and γ, these values were dependent on the sample at hand. For NSVM, BOA was run for 100 iterations, using an expected

improvement selection function via the Tree of Parzen Estimator algorithm[52]. For SVM, random optimization was used for initialization and hyperparameter prior distribution definition[53], similarly followed by Expected Improvement BOA for 30 iterations. While Gaussian Process Upper Confidence Bound and Probability of Improvement selection functions were also trialed and tested, they did not provide significant differences from their Expected Improvement counterparts.

Evaluation of supervised learning algorithms took into account a wide array of different popular evaluation metrics in machine and deep learning. These included; Accuracy, Sensitivity, Specificity, Precision, Recall, Area Under the receiver operator characteristic Curve (AUC), the F-Measure (also known as the F1 Score), Cohen's Kappa (κ) statistic, and model Loss. Each of these metrics are calculated using confusion matrices, measuring the ratio of correctly classified individuals (True Positive & True Negative) as well as miss-classified individuals (False Positive & False Negative). For more details see Supplementary Appendix 6.

Supplementary Tables

Supplementary Table 1. *Robust descriptive statistics of centroid sizes (mm) for each of the species. BFB = Bayes Factor Bound*

Animal	Shapiro W	Shapiro p	BFB	Minimum	IR (0.05)	Median	$\sqrt{BWMV}$	IR (0.95)	Maximum
Vulpes vulpes	0.94	1.1e-02	7.4	1.20	1.89	3.86	1.81	7.77	8.06
Panthera pardus	0.89	3.8e-06	7.8e+03	2.69	2.92	4.76	2.00	9.45	11.76
Canis lupus	0.88	1.6e-06	1.7e+04	2.35	2.81	5.02	2.05	9.53	15.63
Ursus arctos	0.93	1.0e-03	53.3	1.70	1.81	6.36	3.66	13.95	18.45
Lycaon pictus	0.88	7.3e-07	3.6e+04	2.48	3.48	7.40	4.07	19.29	24.84
Crocuta crocuta	0.88	1.4e-06	1.9e+04	2.83	3.84	8.05	3.47	17.50	26.01
Panthera onca	0.88	3.5e-06	8.4e+03	1.85	2.80	7.58	5.10	21.03	31.86
Panthera leo	0.94	5.1e-04	95.1	4.24	4.99	12.12	6.87	26.77	34.25

Supplementary Table 2. *Multivariate Analysis of Variance p-Values.*

	U. arctos	*V. vulpes*	*C. crocuta*	*P. onca*	*P. pardus*	*L. pictus*	*P. leo*
V. vulpes	0.001						
C. crocuta	0.001	0.001					
P. onca	0.002	0.001	0.17				
P. pardus	0.001	0.009	0.001	0.001			
L. pictus	0.048	0.001	0.95	0.8	0.001		
P. leo	0.001	0.001	0.001	0.002	0.001	0.001	
C. lupus	0.015	0.001	0.001	0.001	0.001	0.001	0.001

Supplementary Table 3. *Multivariate Analysis of Variance Bayes Factor Bound (BFB) values. BFB values are represented as odds BFB:1 against H_0.*

	U. arctos	*V. vulpes*	*C. crocuta*	*P. onca*	*P. pardus*	*L. pictus*	*P. leo*
V. vulpes	53.26						
C. crocuta	53.26	53.26					
P. onca	29.60	53.26	1.22				
P. pardus	53.26	8.68	53.26	53.26			
L. pictus	2.52	53.26	7.55*	2.06*	53.26		
P. leo	53.26	53.26	53.26	29.60	53.26	53.26	
C. lupus	5.84	53.26	53.26	53.26	53.26	53.26	53.26

* BFB value represented as odds against H_a

Supplementary Table 4. *Examples of absolute difference ($|d|$), p-Values and Bayes Factor Bounds (BFB) obtained when assessing the robust equivalency of synthetic data and real data using data augmentation on the Pleistocene European Taxa dataset.*

Animal	Measure	PC1	PC2	PC3	PC4	PC5
U. arctos	$\|d\|$	0.004	0.004	0.002	0.007	3.7e-04
	p	1.6e-08	4.0e-37	3.7e-59	9.6e-56	2.9e-70
	BFB	1.3e+06	1.1e+34	7.4e+55	3.0e+52	7.9e+66
V. vulpes	$\|d\|$	0.012	0.005	0.003	0.002	0.003
	p	4.0e-15	2.2e-49	2.3e-56	1.4e-60	1.1e-65
	BFB	2.8e+12	1.5e+46	1.2e+53	1.9e+57	2.2e+62
C. crocuta	$\|d\|$	0.089	0.012	0.006	0.015	0.002
	p	1.2e-07	1.6e-36	6.4e-55	1.3e-53	2.7e-76
	BFB	1.9e+05	2.8e+33	4.6e+51	2.3e+50	7.8e+72
P. pardus	$\|d\|$	0.020	0.004	0.002	0.003	0.005
	p	8.4e-18	7.5e-54	4.1e-65	3.5e-76	1.0e-91
	BFB	1.1e+15	4.0e+50	6.1e+61	6.0e+72	1.8e+88
P. leo	$\|d\|$	0.023	0.013	0.019	0.009	0.009
	p	1.9e-04	1.5e-24	4.2e-38	1.1e-36	1.7e-50
	BFB	2.3e+02	4.5e+21	1.0e+35	4.0e+33	1.9e+47
C. lupus	$\|d\|$	0.007	0.004	0.001	0.007	0.001
	p	8.5e-20	1.9e-80	4.6e-90	2.6e-83	8.8e-86
	BFB	9.9e+16	1.1e+77	3.9e+86	7.4e+79	2.1e+82

Supplementary Table 5. *Examples of absolute difference ($|d|$), p-Values and Bayes Factor Bounds (BFB) obtained when assessing the robust equivalency of synthetic data and real data using data augmentation on the Taxonomic Family dataset.*

Animal	Measure	PC1	PC2	PC3	PC4	PC5
Canidae	$\|d\|$	0.037	0.005	0.003	0.007	0.004
	p	2.1e-16	3.4e-74	6.4e-84	1.0e-76	4.8e-79
	BFB	4.9e+13	6.4e+70	3.0e+80	2.1e+73	4.2e+75
Felidae	$\|d\|$	0.030	0.016	0.005	0.004	0.001
	p	9.0e-04	3.1e-19	3.5e-36	2.9e-40	1.4e-44
	BFB	5.8e+01	2.8e+16	1.3e+33	1.4e+37	2.6e+41
Hyaenidae	$\|d\|$	0.024	0.024	0.007	0.009	0.001
	p	9.0e-10	6.7e-32	1.9e-51	9.0e-43	4.8e-70
	BFB	2.0e+07	7.6e+28	1.7e+48	4.2e+39	4.8e+66
Ursidae	$\|d\|$	0.084	0.001	0.005	0.004	0.004
	p	2.0e-05	5.3e-37	1.2e-58	5.0e-53	3.0e-62
	BFB	1.7e+03	8.3e+33	2.3e+55	6.1e+49	8.7e+58

Supplementary Table 6. *Examples of absolute difference ($|d|$), p-Values and Bayes Factor Bounds (BFB) obtained when assessing the robust equivalency of synthetic data and real data using data augmentation on the Canidae dataset.*

Animal	Measure	PC1	PC2	PC3	PC4	PC5
V. vulpes	$\|d\|$	0.033	0.005	0.002	0.004	0.006
	p	2.8e-12	4.2e-64	6.4e-41	2.3e-46	2.6e-50
	BFB	4.9e+09	6.0e+60	6.2e+37	1.5e+43	1.2e+47
L. pictus	$\|d\|$	0.069	0.012	0.049	0.010	0.012
	p	5.5e-05	2.7e-57	2.9e-23	9.5e-61	7.0e-49
	BFB	6.8e+02	1.0e+54	2.4e+20	2.8e+57	4.7e+45
C. lupus	$\|d\|$	0.030	0.001	0.003	3.0e-04	0.001
	p	3.2e-12	1.1e-95	1.5e-78	9.4e-72	2.2e-84
	BFB	4.3e+09	1.5e+92	1.4e+75	2.4e+68	8.7e+80

Supplementary Table 7. *Examples of absolute difference ($|d|$), p-Values and Bayes Factor Bounds (BFB) obtained when assessing the robust equivalency of synthetic data and real data using data augmentation on the Felidae dataset.*

Animal	Measure	PC1	PC2	PC3	PC4	PC5
P. onca	$|d|$	0.066	0.008	0.005	0.006	0.001
	p	3.3e-06	6.6e-44	2.5e-60	3.1e-58	1.2e-74
	BFB	8.8e+03	5.6e+40	1.1e+57	9.0e+54	1.8e+71
P. pardus	$|d|$	0.019	0.009	0.005	0.001	0.002
	p	1.5e-24	2.1e-73	1.1e-83	1.2e-84	1.4e-93
	BFB	4.5e+21	1.0e+70	1.8e+80	1.6e+81	1.2e+90
P. leo	$|d|$	0.020	0.004	0.005	0.008	0.011
	p	5.8e-06	1.7e-28	1.5e-46	2.1e-47	2.5e-56
	BFB	5.3e+03	3.4e+25	2.3e+43	1.6e+44	1.1e+53

Supplementary Table 8. *Classification results for individual species in the Pleistocene European Taxa dataset. Reported values include; Accuracy (Acc.), Sensitivity (Sens.), Specificity (Spec.), Precision (Prec.), Recall (Rec.), Area Under Curve (AUC), F-Measure (F) and Loss. All evaluation metrics (with the exception of loss) are recorded as values between 0 and 1, with 1 being the highest obtainable value. Values reported over 0.8 are considered an acceptable threshold for powerful classification models. Loss considers values closer to 0 as the most confident models.*

Algorithm	Animal	Acc.	Sens.	Spec.	Prec.	Rec.	AUC	F	Loss
SVM	*U. arctos*	0.89	0.80	0.98	1.00	0.80	0.87	0.89	0.14
	V. vulpes	0.91	0.85	0.97	1.00	0.85	0.84	0.92	0.13
	C. crocuta	0.91	0.96	0.86	1.00	0.96	0.84	0.98	0.11
	P. pardus	0.97	0.76	0.98	1.00	0.76	0.85	0.87	0.20
	P. leo	0.87	0.74	1.00	1.00	0.74	1.00	0.85	0.20
	C. lupus	0.88	0.78	0.98	1.00	0.78	0.84	0.87	0.19
NSVM	*U. arctos*	0.91	0.85	0.81	0.85	0.85	0.96	0.88	0.29
	V. vulpes	0.94	0.98	0.99	0.81	0.98	0.97	0.87	0.35
	C. crocuta	0.92	0.82	0.98	0.82	0.82	0.96	0.87	0.30
	P. pardus	0.79	0.94	0.95	0.94	0.94	0.89	0.86	0.22
	P. leo	0.89	0.91	0.98	0.91	0.91	0.94	0.90	0.10
	C. lupus	0.85	0.94	0.97	0.94	0.94	0.93	0.89	0.30

Supplementary Table 9. *Classification results for individual species in the African Taxa dataset. Reported values include; Accuracy (Acc.), Sensitivity (Sens.), Specificity (Spec.), Precision (Prec.), Recall (Rec.), Area Under Curve (AUC), F-Measure (F) and Loss. All evaluation metrics (with the exception of loss) are recorded as values between 0 and 1, with 1 being the highest obtainable value. Values reported over 0.8 are considered an acceptable threshold for powerful classification models. Loss considers values closer to 0 as the most confident models.*

Algorithm	Animal	Acc.	Sens.	Spec.	Prec.	Rec.	AUC	F	Loss
SVM	*C. crocuta*	0.93	0.87	0.98	1.00	0.87	0.94	0.93	0.08
	P. pardus	0.92	0.85	0.99	1.00	0.85	0.93	0.92	0.10
	L. pictus	0.91	0.85	0.97	1.00	0.85	0.91	0.91	0.12
	P. leo	0.95	0.98	0.90	1.00	0.98	0.99	0.99	0.06
NSVM	*C. crocuta*	0.94	0.92	0.98	0.92	0.92	0.97	0.93	0.15
	P. pardus	0.93	0.94	0.98	0.94	0.94	0.96	0.93	0.02
	L. pictus	0.91	0.96	0.97	0.96	0.96	0.95	0.93	0.22
	P. leo	0.96	0.91	0.99	0.91	0.91	0.98	0.94	0.01

Supplementary Table 10. *Classification results for individual species in the Taxonomic Family dataset. Reported values include; Accuracy (Acc.), Sensitivity (Sens.), Specificity (Spec.), Precision (Prec.), Recall (Rec.), Area Under Curve (AUC), F-Measure (F) and Loss. All evaluation metrics (with the exception of loss) are recorded as values between 0 and 1, with 1 being the highest obtainable value. Values reported over 0.8 are considered an acceptable threshold for powerful classification models. Loss considers values closer to 0 as the most confident models.*

Algorithm	Animal	Acc.	Sens.	Spec.	Prec.	Rec.	AUC	F	Loss
SVM	Canidae	0.97	0.93	0.98	1.00	0.93	0.98	0.98	0.04
	Felidae	0.97	0.97	0.97	1.00	0.97	0.99	0.99	0.03
	Hyaenidae	0.93	0.87	0.97	1.00	0.87	0.94	0.93	0.10
	Ursidae	0.97	0.96	0.98	1.00	0.96	0.98	0.98	0.04
NSVM	Canidae	0.96	0.95	0.99	0.95	0.95	0.98	0.96	0.01
	Felidae	1.00	0.97	1.00	0.97	0.97	1.00	0.99	0.01
	Hyaenidae	0.93	0.99	0.97	0.99	0.99	0.96	0.96	0.19
	Ursidae	0.97	0.94	0.99	0.94	0.94	0.99	0.96	0.05

Supplementary Table 11. *Classification results for individual species in the Canidae dataset. Reported values include; Accuracy (Acc.), Sensitivity (Sens.), Specificity (Spec.), Precision (Prec.), Recall (Rec.), Area Under Curve (AUC), F-Measure (F) and Loss. All evaluation metrics (with the exception of loss) are recorded as values between 0 and 1, with 1 being the highest obtainable value. Values reported over 0.8 are considered an acceptable threshold for powerful classification models. Loss considers values closer to 0 as the most confident models.*

Algorithm	Animal	Acc.	Sens.	Spec.	Prec.	Rec.	AUC	F	Loss
SVM	*V. vulpes*	0.97	0.96	0.99	1.00	0.96	0.98	0.98	0.07
	L. pictus	0.98	0.99	0.97	1.00	0.99	0.99	0.99	0.02
	C. lupus	0.97	0.95	0.99	1.00	0.95	0.97	0.97	0.05
NSVM	*V. vulpes*	0.98	0.98	0.99	0.98	0.98	0.99	0.98	0.01
	L. pictus	1.00	0.96	1.00	0.96	0.96	1.00	0.98	0.01
	C. lupus	0.96	1.00	0.98	1.00	1.00	0.98	0.98	0.01

Supplementary Table 12. *Classification results for individual species in the Felidae dataset. Reported values include; Accuracy (Acc.), Sensitivity (Sens.), Specificity (Spec.), Precision (Prec.), Recall (Rec.), Area Under Curve (AUC), F-Measure (F) and Loss. All evaluation metrics (with the exception of loss) are recorded as values between 0 and 1, with 1 being the highest obtainable value. Values reported over 0.8 are considered an acceptable threshold for powerful classification models. Loss considers values closer to 0 as the most confident models.*

Algorithm	Animal	Acc.	Sens.	Spec.	Prec.	Rec.	AUC	F	Loss
SVM	*P. onca*	0.94	0.89	0.99	1.00	0.90	0.95	0.95	0.07
	P. pardus	0.97	0.96	0.98	1.00	0.96	0.98	0.98	0.03
	P. leo	0.96	0.97	0.96	1.00	0.97	0.99	0.99	0.02
NSVM	*P. onca*	0.93	0.99	0.97	0.99	0.99	0.97	0.96	0.01
	P. pardus	0.99	0.97	0.99	0.97	0.97	0.99	0.98	0.004
	P. leo	0.99	0.96	0.99	0.96	0.96	0.99	0.97	0.004

Supplementary Table 13. *Average time in minutes spent for the training of each of the algorithms, including the bayesian optimization of optimal c and γ values in Support Vector Machines (SVM) and the c values in Neural SVMs (NSVM).*

	SVM	NSVM
European Taxa	16.94	3.75
African Taxa	10.63	3.25
Taxonomic Family	10.97	2.56
Canidae	8.90	2.68
Felidae	8.89	2.48

Supplementary Figures

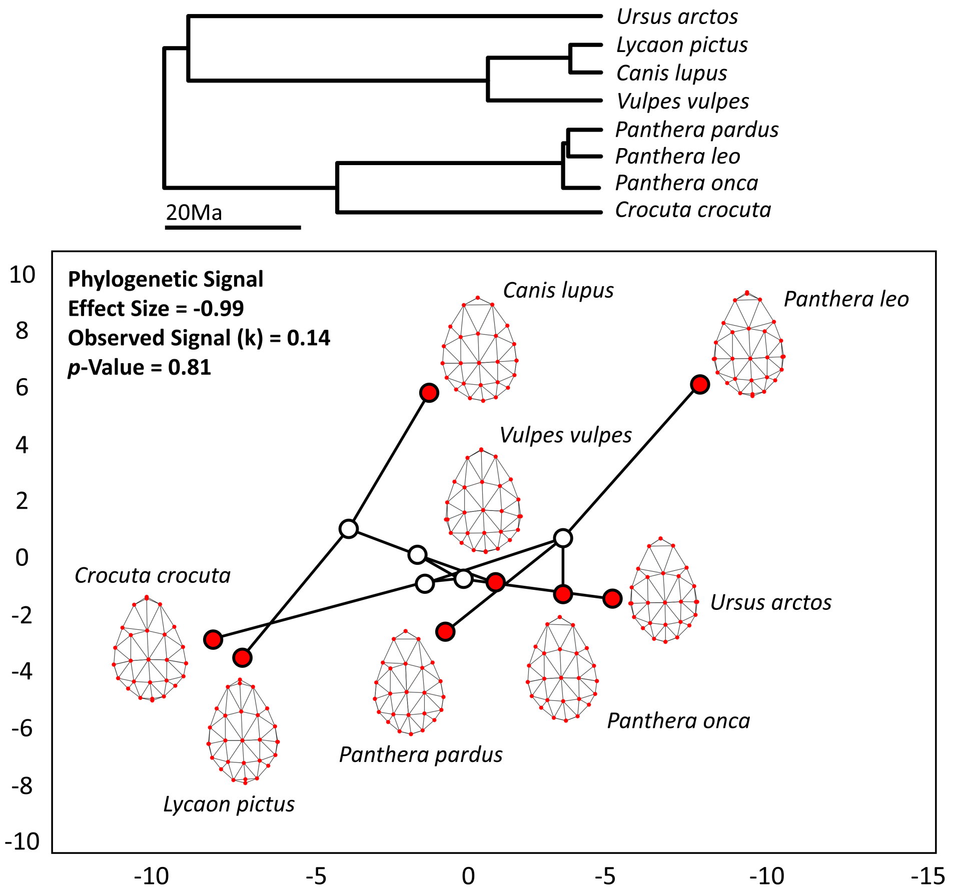

Supplementary Figure 1. Phylogeny of carnivore taxa analysed within the present study and primary morphological tendencies. Data regarding phylogenetic signal represented through morphology has also been included. Figure created using the scikit-learn, geomorph and phytools Python and R libraries.

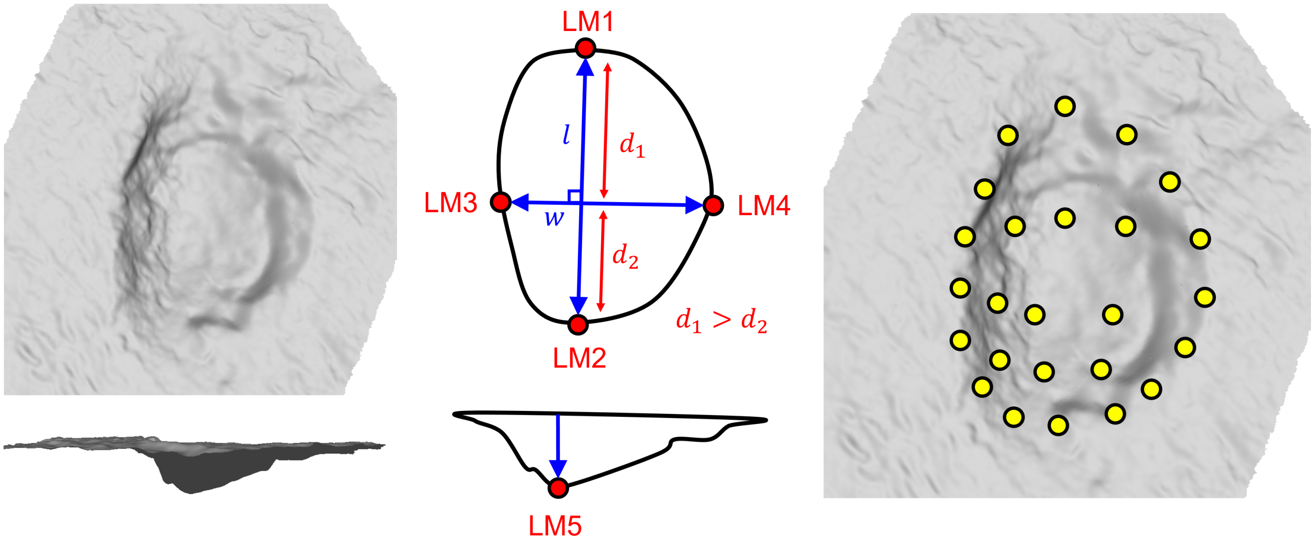

Supplementary Figure 2. Detailed graphical description of landmark models employed. LM = Landmark. *l* = maximum length, *w* = maximum width, *d* = distance. Red landmarks are fixed landmarks, yellow landmarks are semilandmarks whose position are established computationally.

Supplementary Appendix 1 : Carnivores Studied

The present Appendix intends to provide a general overview of the different characteristics presented by each of the animal species included in this paper. The objective of this brief summary hopes to present a number of the conditioning factors that may be of importance when considering carnivore feeding behaviour and thus the nature of the tooth marks observed. Considering how not all of the variables presented here could have been confronted in the present research, we hope that future investigation may be able to shed some light on a number of these features, revealing how some of these physical and ethological attributes may be directly or indirectly linked to the tooth pit morphologies described in this paper.

Tables S14-S18 contain information from a very general nature to the precise allometric components that are frequently used to calculate bite force coefficients. Bite force coefficients usually also include more metric variables regarding the moment arm of the masseter as well as the temporalis, however for specific details about these features we encourage the reader to consult the original work of the relevant authors[54–57]. Needless to say, in the authors' opinion the greatest weight needs to be given to details regarding Carnassial bite force, considering these teeth to be the most used during general chewing activities.

Supplementary Table 14. *Taxonomic details and general attributes of each of the carnivores studied. Details on Maximum Age are very general, as provided by Prado et al. (1984)*[58]*, and may be susceptible to error. Social Organization information was derived from Prado et al. (1984)*[58]

Common Name	Scientific Name	Family	Average Weight (Kg)	Maximum Age (Yrs)	Social Organization
Wolf	*Canis lupus*	Canidae	33.2 / 35.5*	≃ 20	Social
Spotted Hyena	*Crocuta crocuta*	Hyenidae	51.9 / 63.1*	≃ 25	Social
African Wild Dog	*Lycaon pictus*	Canidae	22.0 / 22.4*	≃ 15	Social
Lion	*Panthera leo*	Felidae	156.0 / 162.2*	≃ 24	Social
Jaguar	*Panthera onca*	Felidae	86.5 / 95.5*	≃ 20	Solitary
Leopard	*Panthera pardus*	Felidae	52.5 / 55.0*	≃ 20	Solitary
Brown Bear	*Ursus arctos*	Ursidae	298.9 / 251.2*	≃ 30	Solitary
Fox	*Vulpes vulpes*	Canidae	4.1 / 7.9*	≃ 13	Mixed

* First weights are those reported by Gittleman (1985)[59], second weights reported are those reported by Christiansen and Wrote (2007)[57]

Additional data used in the present study regarded the phylogeny of the different carnivores used. This data was used to create Fig. S1, and was additionally incorporated into some statistical tests (consult Geometric Morphometrics section of main text). All data used was derived from Zhou et al. (2017)[60], Nyakatura and Bininda-Emonds (2012)[61] and Li et al. (2016)[62]. While other sources were also consulted, considering the slight disagreement that exists regarding the precise phylogeny of the

Supplementary Table 15. *General ecology and behavioural attributes of each of the studied carnivores. All data was derived from Gittleman (1985)*[59] *with the exception of running speed*[58].

Common Name	Activity Pattern	Zonation	Vegetation	Latitude (°)	Day Range (Km)	Top Speed (Km/h)
Wolf	Arryhthmical	Terrestrial	Open Grassland & Forest	40	17.5	60
Spotted Hyena	Nocturnal	Terrestrial	Open Grassland	3	-	64
African Wild Dog	Diurnal	Terrestrial	Open Grassland	-3	9.7	72
Lion	Nocturnal	Terrestrial	Open Grassland & Woodland	-3	5.3	80
Jaguar	Nocturnal	Terrestrial	Forest	-	-	80
Leopard	Nocturnal and Crepuscular	Terrestrial & Arboreal	Woodland	1	3.9	58
Brown Bear	Arrhythmical	Terrestrial	Open Grassland & Forest	-	8.8	35
Fox	Nocturnal	Terrestrial	Forest	34	-	50

Supplementary Table 16. *Carnivore diet and feeding habits. Dietary Category was derived from Gittleman (1985)*[59] *and Christiansen & Wroe (2007)*[57]*. Preferable Size of Prey data also originates from Gittleman (1985)*[59]*. Feeding Behaviour information was derived from Prado et al. (1984)*[58]

Common Name	Dietary Category	Preferable Size of Prey	Feeding Behaviour
Wolf	Carnivore	Medium	Sometimes durophagous and scavenger
Spotted Hyena	Carnivore	Large	Hypercarnivore. Durophagous
African Wild Dog	Carnivore	Medium	Hypercarnivore
Lion	Carnivore	Large	Hypercarnivore. Sometimes scavenger
Jaguar	Carnivore	-	Hypercarnivore
Leopard	Carnivore	Medium	Hypercarnivore. Opportunist
Brown Bear	Omnivore	-	Generalist
Fox	Carnivore	Very Small	Generalist

Supplementary Table 17. *Cranial allometric variable measurements for each of the carnivores studied, used to calculate the bite forces and coefficients in Table S18. Variables include; Total Skull Length (SL), Jaw Length (JL), Snout Length (SnL), Maximum gape, Canine Crown Height (CCH), Canine Anteposterior Diameter (CPD) and Canine Mediolateral Diameter (CMD). SL, JL, SnL and Gape were derived from Christiansen and Adolfssen (2005)[54]. CCH, CPD and CMD were derived from Valkenburgh (1987)[63]. Animals are presented in order of Carnassial Bite Force Coefficient values, with the carnivores presenting the strongest carnassial bite force in relation to carnivore size appearing first.*

Common Name	SL (mm)	JL (mm)	SnL (mm)	Gape (°)	Gape (mm)	CCH (mm)	CPD (mm)	CMD (mm)
Wolf	255.3	115.4	197.6	65.0	163.5	26.7	14.2	7.6
Lion	359.7	135.3	274.1	60.2	95.1	43.7	19.8	15.4
African Wild Dog	198.2	152.1	82.1	59.4	101.7	20.8	10.9	7.0
Leopard	188.2	56.1	137.3	63.8	91.6	31.9	14.4	10.4
Jaguar	219.8	68.7	164.7	66.6	70.1	28.6	15.1	12.4
Spotted Hyena	251.0	92.2	183.0	57.5	151.0	25.5	14.7	10.4
Fox	146.0	112.3	54.6	60.3	79.2	14.3	5.7	4.1
Brown Bear	306.1	225.1	104.2	54.7	146.4	38.6*	24.2*	14.6*

* Data from Christiansen and Adolfsen (2005)[54]

Supplementary Table 18. *Biomechanical information for each of the carnivores studied, including Masseter Force (MF), Temporalis Force (TF), Canine Force (CF), Carnassial Force (CaF), Canine Bite Forces calculated from three different sources (CBF-1, CBF-2 and CBF-3), Carnassial Bite Force from two different sources (CaBF-1 amd CaBF-2) and the calculation of Bite Force Coefficients at both the Canine (BFQ-C) and Carnassial teeth (BFQ-Ca). Animals are presented in order of BFQ-Ca values, with the carnivores presenting the strongest carnassial bite forces in relation to carnivore size appearing first. MF, TF, CBF-1 and CaBF-1 measures were derived from Christiansen and Adolssden (2005)[54]. CBF-2 was derived from Wroe et al. (2005)[56]. CBF-3, CaBF-2, BFQ-C and BFQ-Ca were derived from Christiansen and Wroe (2007)[57]. N = Newtons*

Common Name	MF (N)	TF (N)	CBF-1 (N)	CBF-2 (N)	CBF-3 (N)	CaBF-1 (N)	CaBF-2 (N)	BFQ-C	BFQ-Ca
Wolf	1458.0	1886.0	743.0	593.0	493.5	1262.3	773.9	127.3	131.6
Lion	4490.8	5118.3	2152.3	1768.0	1314.7	3405.4	2023.7	123.8	128.1
African Wild Dog	850.0	1815.2	550.5	428.0	374.6	854.0	556.8	131.1	127.7
Leopard	935.4	2929.4	841.5	467.0	621.1	1376.8	964.4	119.8	123.4
Jaguar	932.9	3296.3	765.9	1014.0	887.0	1253.6	1361.2	118.6	121.6
Spotted Hyena	1381.9	3027.9	782.7	773.0	565.7	1421.6	985.5	99.6	115.3
Fox	354.4	568.0	170.3	164.0	144.0	298.4	239.0	100.2	107.5
Brown Bear	1840.5	4500.7	1068.6	751.0	1409.7	1417.6	1894.9	99.3	90.3

Panthera genus, we decided to use the most up to date and recent information.

Finally, we consider it important to point out that not all values and tooth mark results should be considered a complete analogy with those that may be found in the fossil record. The current carnivores provided have been chosen as the closest possible analogies to some carnivores, especially in the case of the Pleistocene European Taxa dataset. Here we have chosen *P. pardus* and *P. leo* as the closest possible analogies with animals such as the Eurasian Cave Lion (*P. spelaea*) and saber-tooth cat species such as the *Megantereon* and *Homotherium*. Likewise, *C. crocuta* was chosen as the closest possible analogy with the Cave Hyena *C. crocuta spelaea*. Needless to say, each of these animals are likely to have differences in biomechanical properties, therefore careful consideration must be made when choosing future comparisons.

Supplementary Appendix 2 : Rib Cortical Density

Numerous considerations were taken into account when collecting tooth mark samples from *P. pardus* individuals, namely concerning; (1) the anatomical element and (2) the size of the animal from which the anatomical element was obtained.

While diaphyses are preferred for the analysis of tooth mark samples, this is not always possible, forcing analysts to adapt to the rules and regulations set forth by natural parks[64]. In the case of *P. pardus*, animals are typically fed axial elements, including articulated ribs alongside all meat packets.

3 primary sources of information were used to assess the suitability of bovine axial elements for the generation of tooth mark samples. These include, in order of significance to the present study;

- Johnson and Chant's 1998 study on beef cattle carcass composition[65].
- Szalma and colleague's 2019 study on the effects of different bone simulation models for surgical drilling times[66]
- Lam and colleague's 1999 study of patterns in bone density in Bovid (*Connachaetes taurinus*), Cervid (*Rangifer tarandus*) and Equid (*Equus* sp.) anatomical elements[67].

The first of these sources[65] consists of numerous calculations of carcass composition using data from 38 different cattle. Each of these cattle weigh between 312 and 388 kg, with measurements concluding between 56.7 and 62.9% (average) of carcasses to be composed of muscle, followed by 20.7-28.5% fat and 13.3-14.6% bone. Additional data show an average of 6.8-13.6mm thickness in fat around the 12th rib. In light of this data, it can be estimated that the general meat packets and associated periosteum of bovine rib cages to be relatively large, providing the bone surfaces enough "coating" to be mostly protected by extreme exertions of force such as those produced during mastication. While the current study is unable to estimate the amount of energy these muscular packages are likely to absorb, it is unlikely that for simple feeding (as opposed to hunting) leopards produce the 2268.7N molar bite force they have been estimated to have[55]. Additionally considering the reported average canine crown height of 31.9mm[63], when adding together factors of cortical hardness and density, our first hypothesis is that leopards are unlikely to produce excessive force enough to produce punctures or excessively deep/large tooth pits on the thickest sections of bovine rib bones.

The second source[66] confronts the use of different types of materials for the simulation of different properties observed during surgical drilling. This study used tungsten 3.1 mm drills to test the mechanical properties of fresh human rib, porcine rib, bovine rib, as well as a number of synthetic materials typically used for the simulation of bone mechanical properties. The study concluded bovine rib to be the most difficult to prepare, arguing the more elevated cortical thickness (2.3 +/- 0.13mm) and density (4490 kg/m^3 [213.5, 334.6]) to be an important conditioning factor.

The study presented by Lam et al.[67], is the first of the three aforementioned studies to present data relevant to archaeological site formation processes. This study uses Computer Tomography scanning to extract digital cross-sections of a wide array of different anatomical elements from *Connachaetes taurinus*, *Rangifer tarandus* and *Equus* sp. adult individuals. From here, the authors derive cross-sections from which to calculate Bone Mineral Density (BDM). BDM measures were extracted using two different calculations (BMD_1 and BMD_2), of which only BMD_1 was considered in the present study. Each cross section was consequently labelled with an ID indicating the type of bone (RI for Rib), and a number associated to where the cross section was extracted (Fig. 1 of Lam et al.[67]). Despite the average weight of *Connachaetes taurinus* is lower (140-290 kg) than the typical european domestic cattle, bovid cortical densities were recorded as being much higher than both the equid and cervid individuals.

Building on the data provided by Lam et al.[67], BMD_1 values were compared between ribs and each of the long bones to test for significance in equivalence using the Two One-Sided equivalency Test (TOST). For the purpose of finding the densest section of the rib possible, only values from RI3 and RI4 were additionally considered. Because the original tables reported (Table 1[67]) only included mean and standard deviation values, magnitude of differences could only be tested using the traditional Welch's t-statistic[39]. TOST in this context evaluates the magnitude of similarities via Superior (εS or ΔS) and Inferior (εI ΔI) equivalence bounds. For samples to be considered equivalent, both ΔS and ΔI need to be associated with

significant p-values. Contrary to typical hypothesis testing using MANOVA or ANOVA, for TOST H_0 considers samples to be different. Under this premise, $p<0.05$ or $p<0.005$ are considered significantly equivalent. Finally, considering that in the present context it is not the same for a long bone to be significantly denser than the cortical of a rib, greater importance was given to the magnitude of ΔS. From this perspective, if the overall test concurs that densities are significantly different, as long as ΔS tested insignificant, then we can conclude that differences between tooth marks found on bovine ribs and tooth marks found on long bone shafts are not of significant importance.

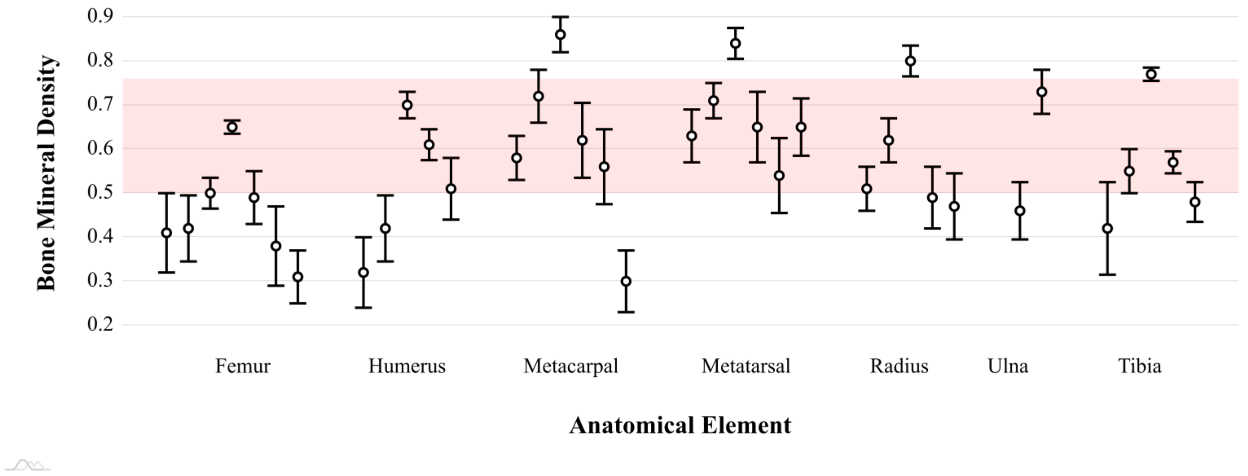

Supplementary Figure 3. Bone Mineral Densities (BDM_1) for different anatomical elements of *Connachaetes taurinus*. Area in red highlights the original density ranges reported for RI3[67]. Figure created using the amCharts4 JavaScript library.

Supplementary Table 19. *Upper and Lower Bound TOST results comparing rib density values reported by Lam et al.[67] with each of the densities reported for long boned. Tost results include p-values and Bayes Factor Bounds. Italicised values indicate density values of skeletal areas to be avoided.*

		Lower		Upper	
Bone	ID	p	BFB	p	BFB
Femur	FE1	3.0e-05	1192	0.62	1.2*
	FE2	9.3e-06	3418	0.72	1.6*
	FE3	6.7e-07	38464	0.64	1.3*
	FE4	1.8e-03	32	1.8e-03	32
	FE5	1.1e-05	2954	0.44	1.0*
	FE6	1.7e-05	1960	0.76	1.8*
	FE7	6.9e-08	324222	1.00	123*
Humerus	HU1	2.1e-06	13400	0.96	10*
	HU2	9.3e-06	3418	0.72	1.6*
	HU3	2.6e-02	3.9	9.5e-05	420
	HU4	2.0e-04	220	0.01	5.8
	HU5	5.6e-05	670	0.23	1.1
Metacarpal	MC1	1.2e-04	352	0.05	2.5
	MC2	4.2e-02	2.8	2.4e-04	184
	MC3	*0.94*	*6.3**	*8.6e-08*	*261792*
	MC4	2.4e-03	25	0.01	7.1
	MC5	5.4e-04	91	0.05	2.4
	MC6	1.6e-03	36	0.01	7.5
Metatarsal	MR1	1.6e-03	37	0.01	11
	MR2	3.7e-02	3.0	8.8e-05	448
	MR3	*0.91*	*4.1**	*1.1e-07*	*210520*
	MR4	4.9e-03	14	4.9e-03	14
	MR5	3.3e-04	138	0.09	1.7
	MR6	3.7e-03	18	3.7e-03	18
Radius	RA1	6.3e-06	4883	0.39	1.0*
	RA2	6.7e-04	75	0.01	8.3
	RA3	*0.64*	*1.3**	*6.7e-07*	*38624*
	RA4	3.2e-05	1111	0.34	1.0
	RA5	3.1e-05	1160	0.42	1.0*
Ulna	UL1	8.1e-06	3874	0.60	1.2*
	UL2	*0.07*	*2.0*	*7.5e-05*	*518*
Tibia	TI1	2.2e-04	202	0.36	1.0
	TI2	5.0e-05	743	0.11	1.5
	TI3	*0.43*	*1.0**	*2.3e-06*	*12126*
	TI4	1.3e-05	2569	0.08	1.8
	TI5	2.1e-06	13400	0.61	1.2*

* BFB value represented as odds against H_a

In summary, and as can be seen through both the visualisation of average density values (Fig. S3), as well as the calculation of ΔS and ΔI equivalence values (Table. S19), the density of bovine rib cortical should be sufficient in providing the closest possible analogy with tooth marks produced on diaphyses by other animals. The only anatomical areas likely to create issues, however, are the central-most parts of intermediate and lower long bone shafts (RA3, TI3, UL2, MC3 & MR3). Nevertheless, considering carnivore tooth damage is more likely to appear on the transition between diaphyses, metaphyses and epiphyses, these observations are not likely to be of concern for the present study.

Supplementary Appendix 3 : *p*-Values, Bayes Factor Bounds & False Positive Risks

In the October of 2017, the American Statistical Association held a 2 day symposium to discuss the use of *p-Values and Statistical Significance*. Product of said symposium can be summarised in the 73rd Volume (Issue Sup1) of *The American Statistician*; a collection of 43 detailed articles on the use and mis-use of *p-Values*, the term *"Statistically significant"*, alongside

recommendations on how to proceed in a world beyond p<0.05[68]. While this collection builds on the original statements and principles proposed by the ASA after the symposium of October, 2015, and published in 2016[69], the more extensive special issue of 2019 strongly discourages the use of the term *"significant"*. Under this premise, careful evaluation of each of the proposals has led us to conclude that the most suitable solution for the present study was to build on the recommendations set forth by Benjamin and Berger[70], and Colquhoun[71].

While a detailed description and analysis of the use and mis-use of p-values goes far beyond the scope of the present study, here we compile a number of the key points that were considered of great importance for the preparation and presentation of our research.

A Brief Overview of p-Value Historiography

The p-value has a long history, arguably dating back to the XVIIIth century, while popularised and standardised during the beginning of the XXth century[72]. Among the many possible authors, notable early usage of p-values include some of the most relevant names in statistics; Laplace (1827)[73], Poisson (1837)[74] and Pearson (1900)[75], *inter alia*. Nevertheless, few authors actually make an attempt at clarifying an acceptable "threshold" or norm from which future analysts can base their work on. While as early as Edgeworth (1885)'s article[76] we can find a possible reference to a certain threshold of importance;

"*by far the greater proportion (namely 0.995)... Accordingly, if out of a set of [...] N statistical numbers which fulfil the law of error, we take one at random, it is exceedingly improbable that it will differ from the Mean to the extent of twice, and* à fortiori *thrice, the modulus... Such is the nature of the law of error*" - Edgeworth (1885), Page 185[76]

To some, at the time, 0.005 was considered excessive and not adaptable to all situations.

Building on this, the most notable and widely-accepted contributions in this field of research, therefore, are not found until the later works of Pearson (1900)[75], Elderton (1902)[77] and Gosset (*a.k.a. Student*, 1908)[78]. From each of these studies, we find valuable insights into early statistical reasoning, namely with the definition of probability distributions that laid down a framework from which p-value calculations would be standardised and developed (χ^2, t & z distributions). Building from these studies, we find the first detailed publications of p-values, with notable contributions by R.A. Fisher. Alongside a series of articles, Fisher published his 1925 book titled *Statistical Methods for Research Workers*[79], containing the referential passage;

"*The value for which p = 0.05... is convenient to take this point as a limit in judging whether a deviation is to be considered significant or not. Deviations exceeding twice the standard deviation are thus formally regarded as significant.*" - Fisher (1925), Page 47[79]

Here it is important to point out specifically the phrase *"twice the standard deviation"*, from which it can be seen that 0.05 was not an arbitrarily chosen value, but more theoretically derived from $1-2\sigma \approx 0.05$. Nevertheless, it is also important to point out that Fisher did not only use 0.05, and in some cases we find his tables including a large number of different p-values, with reference to 0.01 as another threshold for different circumstances.

Nevertheless, Fisher's work was not published without some debate. In a series of studies by Neyman and Pearson[80,81], we find a slightly different approach to defining, not only p-Value calculations in hypothesis testing, but also the reasoning behind defining the 0.05 threshold;

"*...the testing of statistical hypotheses cannot be treated as a problem of estimation*" (pg. 492) "*H_0 is accepted when some alternative H_i is true, the conesquences that follow will depend upon the nature of H_i and its difference from H_0... while it is the 'size' of $p_I(w)$ that matters, it is what may be termed the quality of errors contributing to the risk $P_{II}(w)$, that must be taken into account*" - Neyman and Pearson (Oct, 1933), Page 497[80]

"*We deal first with the class of alternatives for which $\alpha > \alpha_0$. If $\varepsilon = 0.05$... we shall proabbly decide to accept the hypothesis H_0 as far as this class of alternatives is concerned*" - Neyman and Pearson (Jan, 1933), Page 303[81]

Once again, we also find the authors contemplating a more robust threshold of 0.01 depending on the type of probability distributions at hand[81].

Here, Neyman and Pearson approach the concept of hypothesis testing from a different perspective, stating that the acceptance of a hypothesis should not just depend on the H_0 distribution, but also be contextualised with the H_a distribution[80]. From this perspective, these authors search for a means of reducing possible error, with Type I (P_I) and Type II (P_{II}) statistical errors being False Positive and False Negative errors accordingly. In sum, Neyman and Pearson thus discuss a significance level α as 0.05 or 0.01 for P_I risk, while β = 1 - *statistical power* as the P_{II} risk.

While the p-Value threshold between the two approaches does not differ greatly, the means of deriving this threshold is quite different. Under this premise, it could quite possibly be argued that this constant mention of 0.05 across multiple sources,

even if derived from different calculations, is what has generated such confusion in the use of p-Values. As 0.05 appears to resonate throughout history of applied statistics, with few questions regarding the reliability of this threshold, many authors misuse the concept of $p < 0.05$ in hypothesis testing without understanding the true origin of this value.

While the present study will refrain from declaring a preference for one approach or the other, we feel it is important to note both approaches have their advantages and disadvantages, and a general understanding of their origin is necessary in order to correctly evaluate p-value calculations. If we are to consider some of the more recent research into fields of robust statistics, it may be considered that 2σ is not always the best reflection of reality when dealing with non-symmetrical probability distributions. While the t and z distributions are more robust to a number of these cases, presenting longer tails and a means of making general inferences regarding the population, limitations are still present and the definition of a single p-Value threshold is not always as straightforward.

p-Value Calibrations and the use of Bayes Theorem

Beyond the more anecdotal components of this section, and in order to define the methods used to evaluate p-Values in the present study, we think it's important to define what we mean when we refer to p-Values and hypothesis testing in the XXIst century. For this purpose we quote Wasserstein and Lazar's 2016 ASA statement;

"*a p-Value is the probability under a specified statistical model that a statistical summary of the data [...] would be equal to or more extreme than its observed value.*" - Wasserstein and Lazar (2016), Page 131[69]

Among the many papers presented by the ASA's 2019 Special Issue (SI), an important point raised by most authors builds on the exclusively Frequentist perspective most scientists have upon approaching hypothesis testing. In as such, most authors who contributed to the SI raised the point that one of the best means of overcoming p-Value misuse would be to find a compromise between Bayesian and Frequentist approaches. As pointed out by Colquhoun[71], however, this is not an easy task and many analysts are likely to try and avoid these approaches. Part of the Bayesian-Frequentist conflict stems from the distrust many non-statisticians have to the concept of Bayesian Priors[29,71]. For full disclosure, the present author's are also admittedly guilty of this. While in archaeology a typical argument against Bayesian approaches consider that not enough is known about the fossil record in order to construct truly informative priors for Bayesian analysis, in many cases this does not mean that Bayesian approaches should be discarded. As pointed out by most Bayesian practitioners, the use of priors is only one component of the theorem, and while informative priors can be greatly beneficial to the quality of results, weakly-informative priors, or even diffuse priors, are still an option if little is known about the subject matter. Similarly, weakly-informative priors are a popular technique for regularization in many Bayesian learning strategies.

When using Bayes' theorem for hypothesis testing, we can define the following formula (eq. S8);

$$P(A|B) = \frac{P(B|A) \cdot P(A)}{P(B)} \therefore \frac{P(H_a|x)}{P(H_0|x)} = \frac{P(x|H_a)}{P(x|H_0)} \cdot \frac{P(H_a)}{P(H_0)} \tag{8}$$

where x is our data, H_a is the alternative and H_0 the null hypothesis. Observing these equations, it can immediately be seen how Bayesian approaches have the distinct advantage of meeting the requirements set forth by Neyman and Pearson[80,81], whereby the equation considers the test's results not only with regards to H_0 but also in context with H_a. Similarly, Bayesian statistics employ the use of prior and posterior probabilities to quantify levels of uncertainty within a statement[29], providing statisticians with a means of defining their certainty or uncertainty about H_a. Furthermore, this not only implies that the reliability of a p-Value threshold can be empirically assessed, but is also adaptable to the specific data at hand. In Bayesian statistics, this is usually seen by how the final output of a model is not a single value, but a probability distributions of all the possible values[29].

The Bayesian alternative to p-Values in hypothesis testing are known as Bayes Factors (BFs). A Bayes Factor is the likelihood ratio of the marginal likelihoods of H_a and H_0. These values are usually interpreted in ranges, with BF 1 to 3 being weak, 3 to 10 being moderate, 10 to 30 being substantial, 30 to 100 being strong, and over 100 being very strong evidence in favour for H_a[82]. In a similar sense, different authors have made attempts at categorizing p-Values in this manner, with 0.1 to 0.05 implying weak, 0.05 to 0.01 moderate, 0.01 to 0.001 strong, and <0.001 very strong evidence for H_a[83,84]. Nevertheless, in some senses this still assumes a one-size-fits-all p-Value, regardless of specific H_a and H_0 distributions a study may have. Likewise, this can also create an illusion that the p-Value is a calculation of the probability of something being true, while being namely conditioned on the H_0[82].

To directly confront the differences between Frequentist derived p-Values and Bayesian metrics, multiple efforts have been made to propose means of "calibrating" p-value with BFs[70,82,85]. However not all produce the same results[70]. In part this can be explained by some of the issues in which p-values have been defined, whereby the conversion of p-values according to Fisher's definition[79] require a different formula (eq. S9) to those proposed by Neyman and Pearson[80,81] (eq. S10);

$$B(p) = -e\, p \log(p) \tag{9}$$

$$\alpha(p) = \left(1 + [-e\,p\log(p)]^{-1}\right)^{-1} \tag{10}$$

Equation S9 is defined as the lower bound on the odds provided by the data for H_0 and H_a. Wquation S10, on the other hand, is defined as the posterior probability of H_0 based on equation S9, combined with the assumption of an equal prior probability for H_a and H_0 (i.e. 0.5)[85]. Under this premise, we can see how, once again, $\alpha(p)$ fulfills Neyman and Pearson's criteria on defining probability distributions for both H_a and H_0.

In the 2019 ASA SI, we find an interesting development of these equations by Benajmin and Berger, providing 3 recommendations for improving the use of p-Values[70]. The authors; (1) argue that 0.05 should be replaced by 0.005, with values between 0.05 and 0.005 being referred to as "suggestive" evidence rather than "significant"; (2) suggest that analysts report a Bayes Factor Bounds (BFB) value that supports the reported p-Value; and (3) suggest that analystsreport the prior odds of H_1 to H_0.

Firstly, with regards to point (2), a BFB is the upper bound or confidence interval with regards to traditional BFs. Benjamin and Berger thus define BFB as an indication for "*the highest possible BF consistent with the observed p-Value*"[70]. Adapting equation S9 to this statement, BFB can thus be defined as (eq. S11)[70];

$$BF \leq BFB \equiv \frac{1}{-e\,p\,\log(p)} \tag{11}$$

BFBs are generally reported *at most*[70] as odds against the H_0. Their interpretation therefore can consider 0.05, whose corresponding BFB value is 2.44, to mean that there are 2.44:1 odds that H_a be correct. For those who find odds hard to interpret, and would thus prefer a % value, BFBs can easily be converted to percentages via the following formula (eq. S12);

$$P^U(H_a|p) = \frac{BFB(p)}{1 + BFB(p)} \tag{12}$$

The third recommendation presented by Benjamin and Berger requires these values be presented as posterior odds. This is simply carried out by multiplying the BFB value by the prior odds.

One key disadvantage to the use of Bayes factors is that they require the specification of a prior distribution, a topic that frequently produces disagreements or is to complex to define. In some cases where the prior probabilities are unknown, this value can be derived from other variables (see: http://fpr-calc.ucl.ac.uk/[86]), however, as suggested by Colquhoun[71], one fair assumption to make is that these probabilities are equal (50:50, a.k.a. random). This is considered a valid diffuse prior for most hypothesis testing. In light of this, posterior odds for a p-value of e.g. 0.05, would have a posterior probability of $BFB(0.05) \cdot 0.5$.

For ease of comparisons, Table S20 contains a number of p-values calibrated with BFB, $P^U(H_a|p)$ and their corresponding posterior odds.

The final consideration made regarding p-Values in the present study employed the use of the False Positive Risk (FPR) approach by Colquhoun[71].

The FPR is a powerful notion developing a number of the components seen up to this point. FPR attempts to quantify the probability of declaring an effect is real, when it is, in fact, not. This is very similiar to Neyman and Pearson's notions of the α threshold and P_I risk, considering both refer directly to the False Positive Rate of a prediction. In order to calculate this, Colquhoun uses Bayes' theorem to define the likelihood ratio in favour of H_a as eq. S13, where FPR can then be calculated through eq. S14 and in the case where equal prior probabilities for H_a and H_0 are defined we can simplify this equation to eq. S15;

$$L_{10} = \frac{P(x|H_a)}{P(x|H_0)} \tag{13}$$

$$FPR = \frac{1}{1 + L_{10}\frac{P(H_a)}{1-P(H_a)}} \tag{14}$$

$$FPR = \frac{1}{1 + L_{10}\frac{0.5}{0.5}} = \frac{1}{1 + L_{10}} \tag{15}$$

Throughout his article, Colquhoun provides three main formulae for defining L_{10}, each with their own peculiarities. For the purpose of simplicity, however, and so as to provide a means of calibrating Benjamin and Berger's BFB calculations with FPR, the present study has employed the Sellke-Berger approach defined in Appendix A2[71], using eq. S11 as L_{10}. Interestingly, using BFB in this sense forces FPR to be the inverse of $P^U(H_a|p)$, creating a complementary metric that calculates the probability of Type I errors being present alongside the upper-bound probability of H_a being true.

So as not to saturate the main text with large quantities of hard to read numbers, we have provided the following tables and figures as a frame of reference for both the BFB and FPR approaches. The main text, alongside all Supplementary Tables, include BFB ratios according to eq. S11. Posterior probabilities according to Benjamin and Berger's recomendation 0.3 have been included in Table S20. Likewise, this same table contains FPR calculations for each of the corresponding p-Values. Figures S4 & S5 represent a graphical representation of the trend between p-Values and each of the Bayesian-based metrics. One characteristic of Fig. S4 that is of notable importance is the difference between Bayes values when using $\alpha(p)$ and $B(p)$, especially at $p = 0.05$. From here it can be seen that only when $p \approx 0.005$ or closer to 3σ do the two values coincide, likely indicating that 0.005 or 0.003 be a more robust threshold to consider a p-Value as "significant" or not in the eyes of Benajmin and Berger's Recommendation 0.1[70].

When consulting plots of curves for each of these equations, interesting patterns emerge, namely a non-symmetric U-shaped curve (Fig. S4 & S5), indicating BFB or FPR values to not be unique to one single p-Value. This can consequently be interpreted as a means for reversing the focus from H_a to H_0, with values falling on one side of the curve supporting either one of these hypotheses. To find the limit between $p(H_a)$ and $p(H_0)$, a simple experiment was devised, calculating the point of maximum curvature for each curve using steps of 0.0001 across p-Values. This experiment found $p = 0.3681$ as the limit between each hypothesis. Under this premise, in the main text as well as in supplementary tables, p-Values of over 0.3681 were reported as BFBs against H_a and vice versa.

Finally, to observe the effects different priors would have on FPR and $P^U(H_a|p)$ curves, Figure S6 shows how priors effect the morphology of these curves.

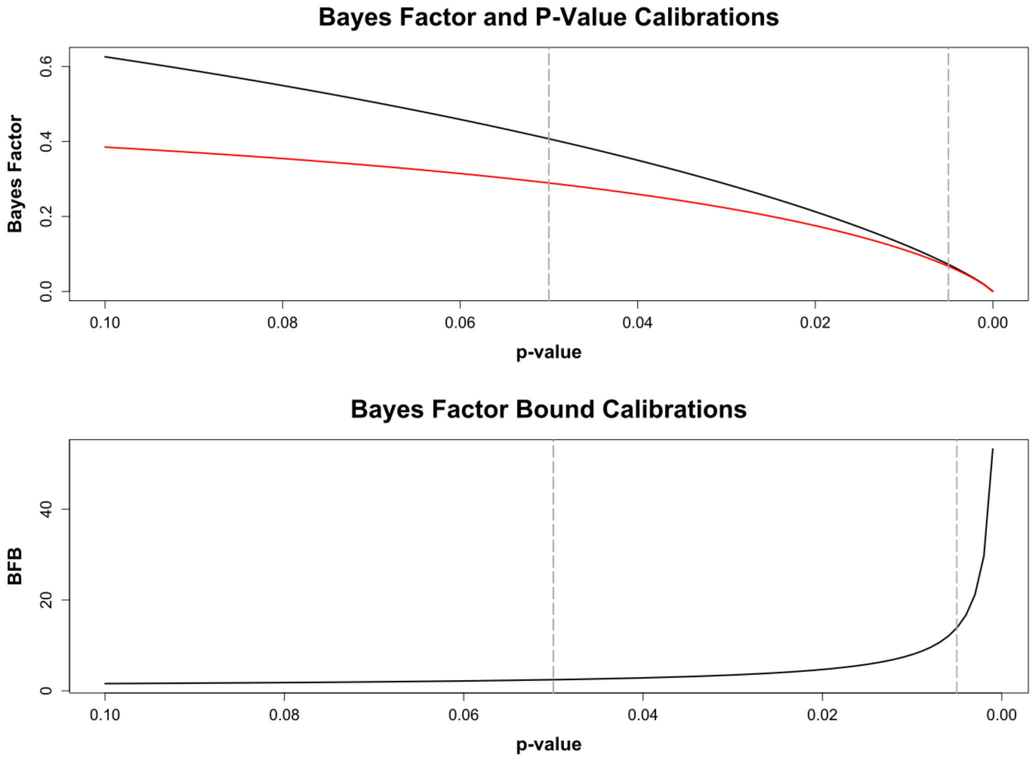

Supplementary Figure 4. p-Value calibration curves for p-Values between 0.1 and 0.0001. Top Panel: Visualisation of calibration curves for different p-Values using the $B(p)$ (black) and $\alpha(p)$ (red) formulas described in equations S9 & 10 respectively. Bottom Panel: Visualisation of the Bayes Factor Bound (BFB) calibration curve for different p-Values. Vertical grey dotted lines mark $p = 0.05$ and $p = 0.005$ respectively. Figure created using base-R.

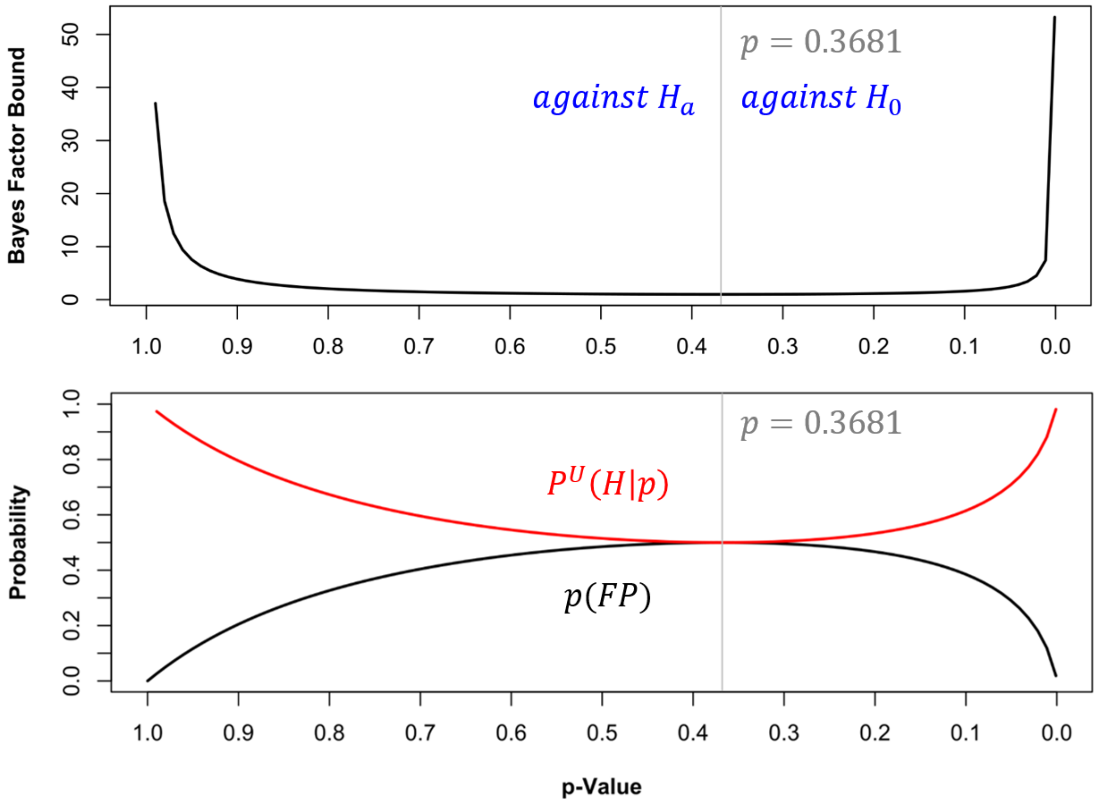

Supplementary Figure 5. p-Value calibration curves for p-Values between 1 and 0.001. Top Panel: Bayes Factor Bound calibration curves (eq. S11). Bottom Panel: Upper bound probability supporting each hypothesis (red; eq. S12) and probability of False Positives (black; eq. S14). Grey line marks the point of maximal curvature along the lines. Figure created using base-R.

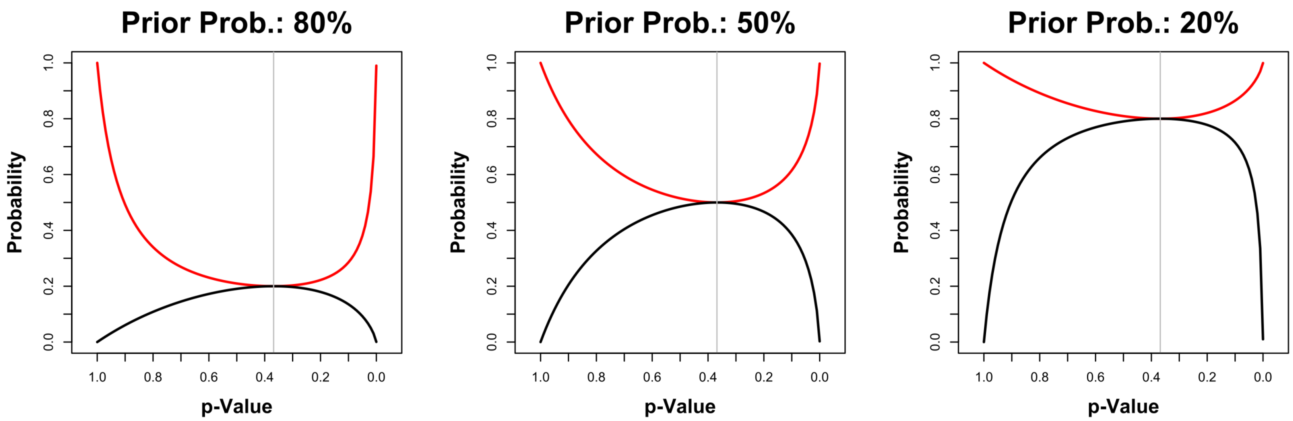

Supplementary Figure 6. Plots describing different variations of Fig. S5's Bottom Panel using different Priors. Grey line marks the point of maximal curvature along the lines. Figure created using base-R.

Supplementary Table 20. *Bayes Factor Bounds (BFBs), Posterior Probability of H_a values ($P^U(H_a|p)$), Posterior Odds of H_a to H_0 values (H_a:H_0), and False Positive Risk (FPR) values, for a number of their corresponding p-Values. Prior odds for H_a:H_0 and FPR calculations were set using a diffuse prior of 1:2*

p	0.368	0.200	0.100	0.050 *	0.010	0.005	0.003 †	0.001	0.0001	0.00001
BFB	1.000	1.140	1.598	2.456	7.988	13.89	21.11	53.26	399.4	3195
$P^U(H_a\|p)$	0.500	0.533	0.615	0.710	0.889	0.933	0.955	0.982	0.998	1.000
H_a:H_0	0.500	0.571	0.799	1.220	3.994	6.943	10.55	26.63	199.7	1598
FPR	0.500	0.467	0.385	0.289	0.111	0.067	0.045	0.018	0.002	0.0003

* p-Values analogous with Fisher's (1925)[79] definition of "deviations exceeding twice the standard deviation"; † adaption of Fisher's (1925)[79] definition using the third standard deviation.

In conclusion, and as evidenced by the numerous equations, tables and figures provided within this appendix, part of the reasons behind our choice of $p < 0.005$ as being significant, and where possible $p < 0.003$, is based on how $p = 0.05$ only implies a 71% probability in favour for our alternative hypothesis when our prior probabilities are 1:2, leaving a 28.9% risk for Type I errors, while $p = 0.005$ lowers this risk to 6.74%, and $p = 0.003$ lowers the risk even further to 4.5 % of Type I errors.

Implementations programmed in R for all formulae presented in the current appendix have been included in the Corresponding Author's GitHub page alongside all code for the present study.

Supplementary Appendix 4 : Central Limit Theorem (CLT)

The Central Limit Theorem (CLT) is a key concept in probability theory, and in as such has proven a valuable concept for data scientists. While a number of CLT variants exist, some of the most classic versions of CLT are associated with the french statistician: Pierre-Simon Laplace[87]. In the simplest terms, the CLT states that no matter the shape of a population distribution, the sampling distribution of sample means approaches a normal distribution (eq. S1) as the sample size increases. Laplace was even able to show, similar to some previous research by Abraham de Moivre (1733), how a binomial distribution, such as that formed by the throwing of dice, can be approximated to a Gaussian distribution as the number of die throws increases.

While many types of probability distribution exist, one of the most popular and central distributions to statistics is that defined by Carl Friedrich Guass[88]. The Gaussian distribution is also known by a number of different names, one of the most common being the "Normal" distribution ($\mathcal{N}$), and is central to many elements of statistics. The Gaussian distribution is popular because of many convenient features; the probability distribution function is symmetric, well defined by the mean (μ) and standard deviation (σ), and seems to appear everywhere (hence the CLT). Nevertheless, this distribution is also unfortunately overused and, in many cases, misused, inspiring the development of a number of statistical sub-disciplines such as that of robust statistics.

While the current authors strive to ensure that the statistical tests performed within the current research are robust (See "Robust Statistics" of the main text), and not entirely dependent on hidden mathematical assumptions, it cannot be denied that the Gaussian distribution is *central* to statistical theory. In light of this, the present appendix intends to show how the samples used within the present study can still be converged on a Gaussian distribution for the purpose of MCMC modelling.

CLT Theory

To expand on some elements touched upon in the Appendix 3, an interesting component in early investigation into p-Value calculations touched on concepts of normality[75,77,78], especially in the context of sample size. While many elements of statistical analyses are dependent on the Gaussian distribution, a wide array of different distributions also exist. One very popular distribution used frequently in hypothesis testing is the t-Distribution, sometimes referred to as Student's t-distribution. The term "Student" originates from Gosset's use of the pseudonym "Student" when publishing his research in 1908[78]. A simplified formula for the t-distribution is (eq. S16);

$$t = \frac{\bar{x} - \mu}{\sigma/\sqrt{n}} \tag{16}$$

Where μ is the population mean, $\bar{x}$ is the sample mean, σ is the sample standard deviation and n is the sample size. As can be seen both in equation S16 and the equation for the standard deviation (eq. S17);

$$\sigma = \sqrt{\frac{\sum (x_i - \mu)^2}{n}} \tag{17}$$

both equations are dependent on sample sizes n and thus sensitive to a certain degree of freedom. The reason behind this is; as the sample size increases, the t-distribution will approximate a Gaussian distribution. Advantages of using this approach are (1) the t-distribution is more robust to outliers based on the probability density function's heavy tails[29], and therefore (2) can provide better inferences on the population from smaller samples.

This is an important notion to highlight, considering how as the sample size increases, the distribution appears more "normal". From this perspective, many analysts consider sample size to be a primary cause for a continuous sample's lack of normality. Under this premise, while hypothesis testing should take inhomogeneity into account (parametric vs non-parametric tests), these observed deviations from normality should not always be considered a true reflection of the populations' distribution.

Present Application

Considering how the objective of our study is to use augmentation techniques to simulate a sample's original population, these notions of normality with regards to sample size can be considered fundamental. While the empirical samples themselves do not always comply with the definition of a Gaussian distribution, this is not to say that the observed kurtosis and skewness values are representative of the *population*'s normality, and thus a carefully adapted use of Gaussian priors can be justified for the augmentation of our datasets using MCMC. To present a final attempt at justifying this decision, a simple experiment was devised to show how sample size is likely to distort our view of whether a population is normally distributed or not.

Figure S7 presents 4 multivariate (2D) theoretical samples of varying sizes. The x axis of each has been further represented univariately using a density plot in the upper panels (Fig. S7). In each case, the population probability distribution function has also been represented (red dotted-line), calculated using equation S1, with $\mu = 0$ and $\sigma = 1$. The population was then sampled from, first extracting 10 points, then 50, then 100, then 1000. As can be seen, simply from a visual perspective, the empirical (black) distribution does not begin to resemble the theoretical (red) population distribution until at least $n = 100$.

Table S21 provides complementary descriptive statistics for each of these graphs (focusing simply on the univariate x-axis for ease of describing the distributions). First normality is tested. A number of tests exist to detect whether a sample is Gaussian or not. The present study (both here and in the main paper) used primarily the Shapiro-Wilk's test for normality[89,90], chosen due to its observed statistical power when compared with other methods[91]. As can be seen, despite the small sample size in the first case, all 4 samples test as homogeneous, with $n = 50$ coming the closest to rejecting the H_0. Nevertheless, under the assumptions of a normal distribution with a predefined density ($\mu = 0$, $\sigma = 1$), we should expect (1) the mean μ and median $\tilde{x}$ to coincide at 0, the standard deviation and Normalised Median Absolute Deviation (eq. S3) to also coincide at 1, while Skewness should reflect a symmetrical distribution (≈ 0) and relatively low kurtosis (≈ 0). As can be seen in the table, these patterns only appear for $n = 100$ and $n = 1000$.

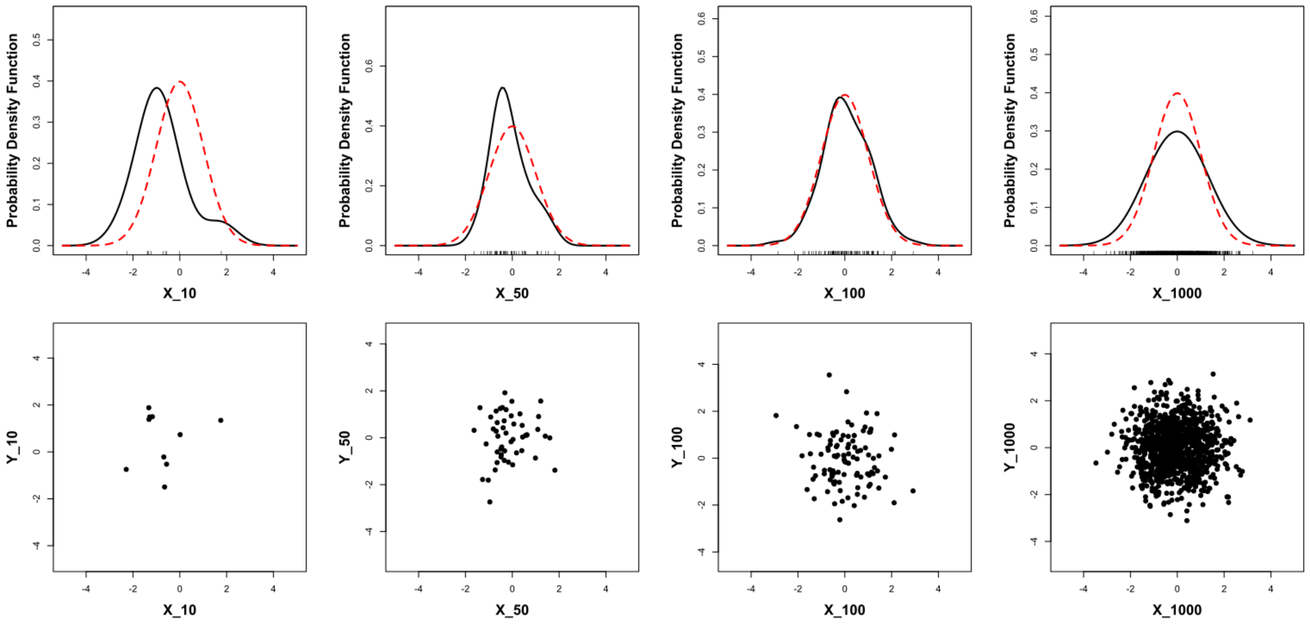

Supplementary Figure 7. Sample Sizes. Figure created using base-R.

Supplementary Table 21. *Sample Size experiments. BFB = Bayes Factor Bounds.* μ *= mean,* $\tilde{x}$ *= median,* σ *= 1st standard deviation, NMAD = Normalized Median Absolute Deviation.*

Sample Size	Shapiro W	Shaprio p	Shapiro BFB	μ	$\tilde{x}$	σ	NMAD	Skewness	Kurtosis
10	0.877	0.122	1.432	-0.75	-0.929	1.07	0.558	0.963	0.424
50	0.961	0.099	1.604	-0.12	-0.322	0.78	0.559	0.582	-0.204
100	0.993	0.873	3.118*	0.067	-0.016	0.98	0.892	0.016	0.299
1000	0.999	0.771	1.835*	-0.015	-0.004	1.00	1.049	0.001	-0.149

* BFB value represented as odds against H_a

Finally Fig S8 shows how one of our samples (Lion PC1) can use the CLT to approximate a Gaussian distribution. Top pannels present the univariate density functions, while the bottom pannels present Quantile-Quantile plots (Q-Q plots). Samples need to fall along the red line in Q-Q plots in order to comply with normality. As can be seen, the original distribution presents a large positive tail resulting in asymmetry (skew = 0.97). Consequently, the sample tests as non-Gaussian ($w = 0.93$, $p = 0.0002$, BFB = 163, FPR = 0.006). The plot on the left represents the distribution of means from 300 permuted samples (without replacement) of size 30 from this distribution. As can be seen, this data extracted from the original distribution approximates a Gaussian distribution, with very low asymmetry (skew = 0.05), and shapiro-wilk results supporting H_0 ($w = 0.997$, $p = 0.89$, BFB = 3.46 against H_a, FPR = 0.22). In both the aformentioned cases, FPR used uninformative 0.5 priors.

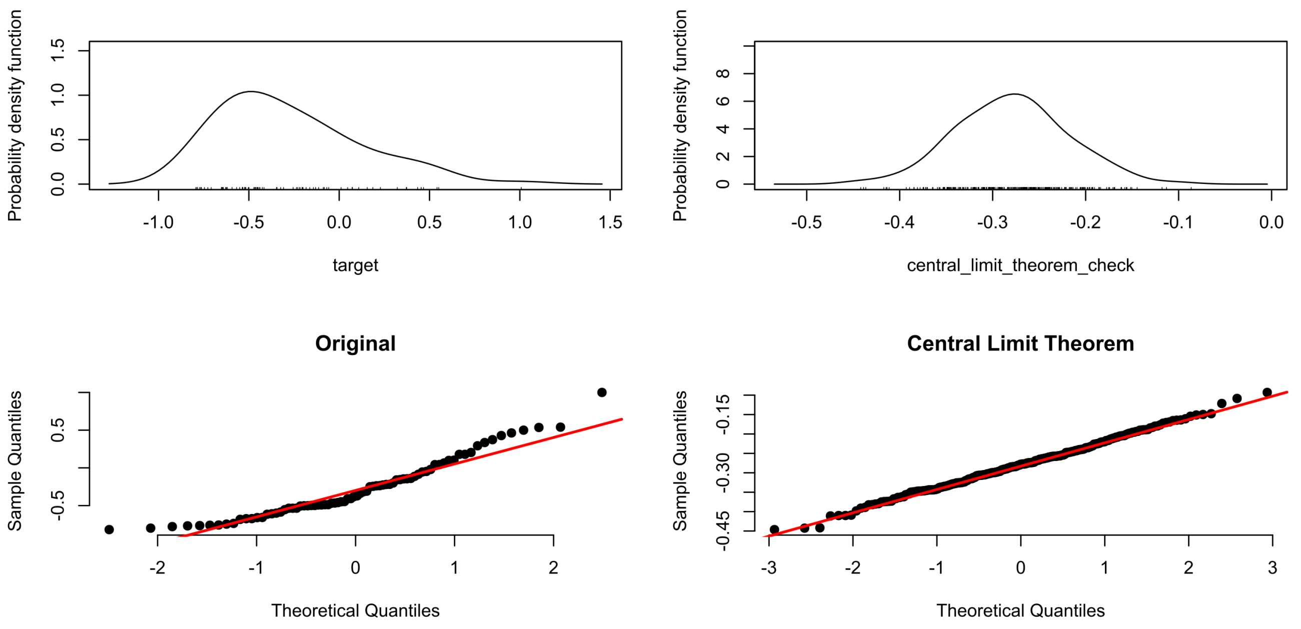

Supplementary Figure 8. Upper Panels: Probability Density Functions for one of the dimensions of the lion sample. Lower Panels: Q-Q plots testing for normality. Left Panels: The original sample (Lion PC1). Right Panels: Distribution of sampled means from the original sample. Figure created using base-R.

Supplementary Appendix 5 : Neural Network Activation Functions

One of the key advantages presented by the use of Neural Networks (NNs) are their highly versatile non-linear attributes. Upon combining multiple layers of neurons, each with differing densities, NNs have proven capable of approximating any distribution function, solving advanced questions in multiple fields[22].

A neuron is defined through simply multiplying an incoming signal ($x_1, \ldots, x_n$) with a set of weights ($w_1, \ldots, w_n$), such that the value of a neuron h is (eq. S18);

$$h_i = \sum_{i=1}^{n} w_i x_i \tag{18}$$

Here we have chosen to use h to represent a single neuron with h representing the term "hidden". A neural network is thus built up of multiple hidden neurons, all fully connected with a set of weights to other hidden neurons in the next layer. This continues until the final output layer.

With the intention of replicating biological functions of the human brain, the first conceptualisation of the neuron (a.k.a. perceptron), also introduced the concept of an activation function. An activation function attempts to determine whether neurons are "activated" or not, thus controlling the flow of information between the inputs and the outputs. From this perspective, a complex combination of multiple neuron layers with varying densities can thus map out complex non-linear fluctuations of information that helps map a set of inputs ($x_1, \ldots, x_n$) to their corresponding outputs (y_i). The activation function thus takes the value of the neuron (h) as input ($f(h_i)$ or essentially $f(x)$), and decides to what extent this information gets passed on to the next neuron.

Rectified Linear Units (ReLU)

Arguably the most famous and commonly used activation function is the Rectified Linear Unit (ReLU). ReLU can be considered a non-linear adaptation of a typical linear function. While linear functions are represented simply as $y = x$, ReLU provides a means of turning off/on a neuron by only allowing certain values through. This was originally conceptualised as a means of filtering negative values, with ReLU only allowing positive ($h > 0$) information through. Mathematically this is simply (eq. S19);

$$f(x) = max(0, x) \tag{19}$$

While adaptations of ReLU exist that slightly adjust this filter gate[92–95], ReLU is one of the most widely used activation functions for numerous reasons. Firstly the simplicity of the function, from a computational level as well as how simple it is while remaining non-linear. Secondly, the derivative ($f'(x)$) of ReLU is also easy to calculate, considering it is half of the linear function (eq. S20);

$$f'(x) = \begin{cases} 1 & x > 0 \\ 0 & x < 0 \end{cases} \tag{20}$$

Derivatives are used in calculus to calculate the slope of a function. Derivatives are therefore fundamental when performing weight optimization so as to know how to adjust a weight so as to reduce the error. This is commonly known as Stochastic Gradient Descent (SGD). Likewise, the second derivative of ReLU, is simply (eq. S21);

$$f''(x) = 0 \tag{21}$$

Derivative calculations become increasingly more important the deeper the network is. From this perspective, it is fundamental that derivatives be easy to compute.

From all three equations, it can be seen how ReLU has the distinct advantage of being; (1) non-linear, (2) easy to compute, and (3) easy to optimise when using SGD and its variants.

ReLU however has some disadvantages. Firstly, the cut-off limit can often be considered too harsh, with the hinge of this function being abrupt and thus leading to the unnecessary dis-activation of some neurons. In some of these cases, alternatives such as the Leaky ReLU function are powerful, but in others this can sometimes allow too much information to pass through. The latter is often cause for exploding gradients, which is the precise opposite objective of ReLU. Under this premise, while being "bounded" bellow (i.e. not allowing information bellow a certain threshold through) is advantageous, having too hard a cut off may be an issue for gradient flow.

Finally, from the precise perspective of the present study, considering how NNs are used as kernel functions, where the outputs are directly fed into a Support Vector Machine (SVM), we personally felt that such a strict dis-activation of neurons would produce overly sparse vectors, e.g.;

$$\vec{v} = [0, 0, 0, 0, x_1, 0, x_2, 0, \ldots, 0, x_n]$$

which could possibly over-restrict the learning process for SVMs. Under this premise, we decided to gate neurons with a softer lower-bound so as to allow *some* information to pass through, but not too much information so as to not damage the learning process.

Self-Gated Rectified Activation Functions: Swish

Swish is an adaption of ReLU originally proposed in 2017 by Ramachandran and Lee (2017)[47]. The authors describe this function as;

"*Like ReLU, Swish is unbounded above and bounded below. Unlike ReLU, Swish is smooth and non-monotonic*" - Ramachandran and Lee (2017), Page 2[69]

The Swish activation function is thus defined mathematically as (eq. S22-S24);

$$f(x) = x \cdot \frac{1}{1+e^{-x}} \tag{22}$$

$$f'(x) = \frac{1}{1+e^{-x}} + x \cdot \frac{e^{-x}}{(1+e^{-x})^2} \tag{23}$$

$$f''(x) = \frac{e^{-x}}{(1+e^{-x})^2} + \left(\left(\frac{e^{-x}}{(1+e^{-x})^2} \right) - x \left(\frac{e^{-x}}{(1+e^{-x})^2} - e^{-x} \left(\frac{2\,(e^{-x}\,(1+e^{-x}))}{\left((1+e^{-x})^2\right)^2} \right) \right) \right) \tag{24}$$

Before delving in to the advantages of Swish, one notable component of the fomulae that catches the eye is the similarities between Swish and the Sigmoid function (eq. S25). The Sigmoid function is famously attributed to algorithms such as those found in Logistic Regression, can also be found in elements of Gradient Boost classification algorithms, as well as being the typical activation function used in the final output of a Binary-Classification NN. The multi-output version of Sigmoid, in fact, is the Softmax function, previously defined in Equation S7 of the present document. Sigmoid (eq. S25) is a powerful activation function, that works by taking an input and returning a value between 0 and 1. The derivatives of Sigmoid (eq. S26 & S27) are equally useful as they are relatively easy to compute. Sigmoid, however, contrary to functions such as ReLU and Swish, is bound both above and below; the maximum value that can be computed is 1, and the lowest value is 0.

$$f(x) = \frac{1}{1+e^{-x}} \tag{25}$$

$$f'(x) = \frac{e^{-x}}{(1+e^{-x})^2} \tag{26}$$

$$f''(x) = -\left(\frac{e^{-x}}{(1+e^{-x})^2} - e^{-x} \frac{(2\,(e^{-x}\,(1+e^{-x})))}{\left((1+e^{-x})^2\right)^2} \right) \tag{27}$$

Returning to Swish, it can be seen that Equation S22 incorporates Equation S25 by multiplying the input x by the Sigmoid function (eq. S25). Product of this is a smooth version of ReLU, with a soft lower bound, and no upper bound. Swish therefore approximates to ReLU, but does not have as harsh a hinge that separates negative values from positive values. This essentially allows very small negative values to exist, thus improving gradient flow. This former point helps decrease the possibility of sparse vectors as input to SVM while allowing the most important features through.

Figure S9 visually presents the curves for ReLU (eq. S19-S21), Sigmoid (eq. S25-S27), and Swish (S22-S24). Here it can be seen how Swish is effectively the combination of ReLU and Sigmoid, with very similiar derivatives to Sigmoid, a similiar $f(x)$ morphology to ReLU, but a smoother cut off. From this perspective, and as pointed out by Ramachandran and Lee (2017)[47], Swish is a non-monotonic smooth activation function that works very well on very deep neural networks, while still preserving the advantages of non-linearity with no upper bound for improved weight optimization.

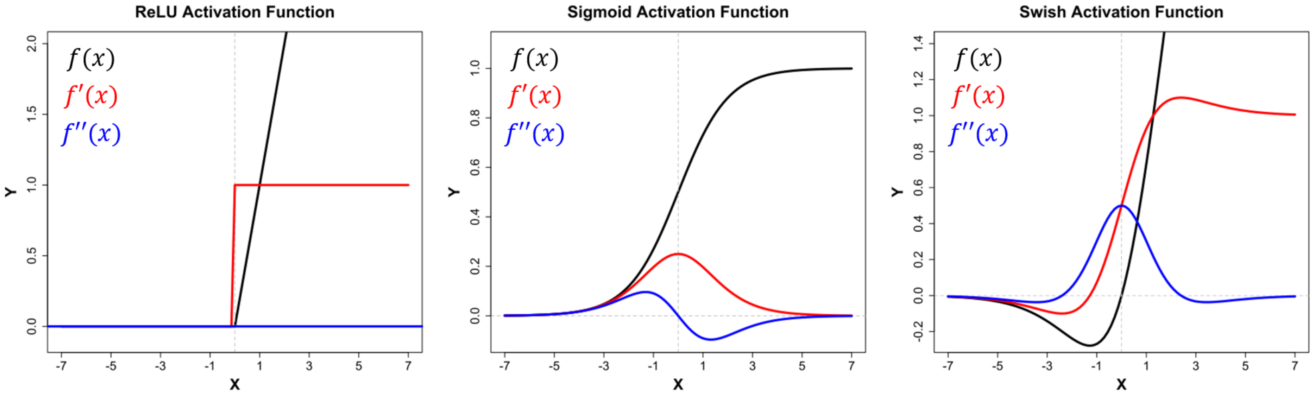

Supplementary Figure 9. Graphical Representation of 3 Activation Functions ($f(x)$) alongisde their first ($f'(x)$) and second ($f''(x)$) derivatives. Activation functions include the Rectified Linear Unit (eq. S19-S21), Sigmoid (eq. S25-S27) and Swish (eq. S22-S24)[47]. Figure created using base-R.

R-Code used to produce graphs and calculate derivatives has been included in the corresponding author's GitHub page alongside all other code related to the present research.

Supplementary Appendix 6 : Computational Learning Evaluation Metrics

The evaluation of classification algorithms in computational learning is of the upmost importance, however can often be considered misleading. In most contexts, the words "80% classification" would be seen as a powerful algorithm, however this is often not the case as will be presented here. In contexts such as medicine, for the construction of diagnostic tools, more weight is given to values such as sensitivity and specificity. In other cases, such as economics and supervised anomaly detection in bank fraud, precision and recall are more reliable. In general, Precision/Recall values are considered metrics used for imbalanced classification, while sensitivity and specificity are less sensitive to imbalance, thus being more reliable in general classification problems. The term imbalance here refers to where one of the labels we wish to classify is underrepresented in the dataset.

In bank fraud, for example, there may be 100 frauds out of 10,000 examples. In this case, the imbalance is described as 1:100. In medicine, this would be the case of classifying a rare disease. A balanced problem, on the other hand, would be in the case where we have the same number of healthy individuals as unhealthy (1:1). Finally, it is not the same for slight imbalance to exist (1:10) as slightly more extreme cases (1:100) as very extreme imbalance (1:1000). In each of these cases different evaluation metrics will be more or less reliable.

From this perspective, each of the metrics are defined for a particular reason, however all carry out very similar functions. Evaluation metrics can thus be defined as a means of not only calculating classification accuracy, but also a means of evaluating how often an algorithm is right, wrong, not wrong or right in saying the opposite. While this can sometimes be confusing, consider the following situation; it is not the same for a doctor to diagnose a patient with an illness and they are actually healthy, as it is to diagnose a patient as healthy and they are in fact ill. In the former case, this could lead to a law suit, while in the latter this could even lead to death.

In order to perform these evaluations, a Confusion Matrix is defined;

$$M = \begin{bmatrix} TP & FP \\ FN & TN \end{bmatrix}$$

where P refers to positive, N to Negative, T to True and F to False. In the case of our aforementioned patient, a False Positive would be where we diagnose the patient as ill when they are in fact not. A True Positive would be if we diagnose the patient as ill and it turns out that this is true.

For simplicity as well as the purpose of demonstrating how evaluations work, each of the three matrices that will be tried and tested in this Appendix are defined in the context of binary classification (ill or healthy, fraud or not-fraud). Evaluation metrics for binary classification in this context can thus be defined as;

$$Accuracy = \frac{TP + TN}{TP + FP + FN + TN} \tag{28}$$

$$Sensitivity = \frac{TP}{TP+FN} = Recall \tag{29}$$

$$Specificity = \frac{TN}{FP+TN} \tag{30}$$

$$Precision = \frac{TP}{TP+FP} \tag{31}$$

With F-Measure (F):

$$F = \frac{2 \cdot Precision \cdot Recall}{Precision + Recall} \tag{32}$$

and Kappa (κ);

$$P_e = P_{pos} + P_{neg} \tag{33}$$

$$P_{pos} = \frac{TP+FP}{TP+FP+FN+TN} \cdot \frac{TP+FN}{TP+FP+FN+TN} \tag{34}$$

$$P_{neg} = \frac{FN+TN}{TP+FP+FN+TN} \cdot \frac{FP+TN}{TP+FP+FN+TN} \tag{35}$$

$$P_o = \frac{TP+TN}{TP+FP+FN+TN} \tag{36}$$

$$\kappa = \frac{P_o - P_e}{1 - P_e} \tag{37}$$

For ease of calculation, AUC and loss were excluded from this appendix. AUC however can be seen as a powerful balance between Sensitivity, Specificity and Precision.

For the purpose of this demonstration we have defined three confusion matrices; A is an example of a very poor classifier with a misleading high accuracy; B is a slightly more evident poor classifier, with relatively high accuracy; and C is a relatively good classifier with more balanced accuracy. Matrices A and C both have 80% classification accuracy, a threshold that would normally be considered "good". Matrix B has 70% accuracy, a rate that is not particularly good but could be worse.

$$A = \begin{bmatrix} 80 & 20 \\ 0 & 0 \end{bmatrix}, \quad B = \begin{bmatrix} 60 & 20 \\ 10 & 10 \end{bmatrix}, \quad C = \begin{bmatrix} 50 & 10 \\ 10 & 30 \end{bmatrix}$$

The results for all three matrices can be consulted in the following table;

Supplementary Table 22. *Evaluation matrices for Confusion Matrices A, B and C.*

Metric	Matrix A	Matrix B	Matrix C
Accuracy	0.80	0.70	0.80
Sensitivity	1.00	0.86	0.83
Specificity	0.00	0.33	0.75
Precision	0.80	0.75	0.83
Recall	1.00	0.86	0.83
F-Measure	0.88	0.80	0.83
Kappa	0.00	0.21	0.58

As can be seen in the table, the most obvious observations is that 80% accuracy is still possible even if an algorithm is very weak. In this case, the 80% accuracy is completely unreliable. Likewise, for Matrix B, while the accuracy falls 10%, in comparisons with other metrics for Matrix A it is still a much more powerful classifier. When performing the maths, for example, this appears increasingly more obvious as Kappa is calculated. If we can consider how the number of True Negatives is 0 in Matrix A, the first fraction in eq. S35 will equate to 0. A scalar 0 multiplied or divided by anything will condition the formula to reach 0, thus lowering the κ statistic drastically. Likewise, considering how Matrix B presents evaluation metrics higher than Matrix B, the low TN value still conditions κ to be much lower. Finally, even in Matrix C, where most values sensitivity, precision and recall values are above the acceptable 0.8 threshold, κ still proves to be a strict statistic determining Matrix C to be a moderate result.

The next interesting component to consider is how, even in matrices that have specifically been designed to be bad (A and B), both Precision, Recall and F-Measure values are very high. This is quite misleading, and could raise the question: are these really reliable? To simply answer this question, consider the following matrix;

$$D = \begin{bmatrix} 85268 & 28 \\ 31 & 116 \end{bmatrix}$$

simply extracted from the following blog;

https://blog.usejournal.com/credit-card-fraud-detection-by-neural-network-in-keras-4bd81cc9e7fe

Matrix D represents a classification algorithm for credit card fraud with an extreme imbalance of 147:85384. The resulting precision and recall in this case study comes to 0.99967 and 0.99963 respectively. This indicates that the algorithm that produced matrix D was a very powerful classifier for both the smaller and larger class. Specificity, however, would not truly reflect this power with a value of only 0.81.

From this perspective, it can be seen that different evaluation metrics are designed for different purposes, and the choice of metrics in a study *should* reflect this. On a taxonomic level, the present study only has a single sample that is "imbalanced" with regards to the rest (*V. vulpes*), needless to say, this imbalance is relatively weak ($\approx$ 5:8). Beyond this, the strongest imbalance present is in the Taxonomic Family dataset, when comparing felidae tooth marks with ursidae tooth marks. This case of imbalance is $\approx$ 7:24. So as to ensure our tables are comparable with those published by other authors, all evaluation metrics have been included. Nevertheless, the authors strongly encourage the reader to consider Sensitivity, Specificity, Kappa, AUC and loss metrics over others, so as to ensure the correct intepretation of our results.

Supplementary Appendix 7 : Computational Learning Performance on Non-Augmented Datasets

The present Appendix provides adaptations of Tables 1 & S8-S12 using non-augmented datasets for the training of supervised computational learning algorithms.

A number of important components can be noted from these tables;

- Even without data augmentation, many algorithms are able to reach state of the art performance. On average, accuracy only appears to drop 4% for SVM and 6% for NSVM. Likewise, with the exception of the Pleisteocene European Taxa dataset (κ = 0.75) and the Taxonomic Family dataset (κ = 0.61), κ values remain above the standard 0.8 threshold.

- The greatest difference between augmented and non-augmented performance can be seen in the calculation of loss values. Central tendency calculations reveal a loss of 9% confidence when performing predictions without the aid of data augmentation (Table 1 & S23). When observing performance on specific datasets, it can be seen how the Taxonomic Family dataset is the most affected, with reports of loss values over 0.5. This is an evident product of dataset imbalance, whereby the smallest classes present the highest loss values. This ties strongly with observations presented by Courtenay and González-Aguilera[25], observing a considerable drop in loss and improvement in decision boundary definitions when using augmented datasets to train Linear Discriminant models.

- Strongly tied to the previous point, sample imbalance in the Taxonomic Family dataset also produces a change to precision and recall metrics. As detailed in Supplementary Appendix 6, these two metrics are more suited for the evaluation of algorithms trained on imbalanced datasets. While the use of data augmentation in the present study was used to reduce these effects, the moment they are excluded, each of these performance metrics drop notably on the poorly represented samples. Here it can be seen how Hyaenidae (n = 86) and Ursidae (n = 69) present the lowest recall values (Hyaenidae = 0.37, Ursidae = 0.42). In some cases precision is also seen to drop (Hyaenidae = 0.66, Ursidae = 0.68), resulting in low F-Measures (Hyaenidae = 0.54, Ursidae = 0.59). Canidae (n = 222) and Felidae (n = 243), on the other hand, are well

represented, and result in higher precision and recall values (Precision $\approx$ 0.88, Recall $\approx$ 0.79). The moment augmentation is used to balance these datasets, however, all 4 samples present similarly high evaluation metrics (Precision $\approx$ 0.98, Recall $\approx$ 0.95).

Supplementary Table 23. *Overall classification results obtained for non-augmented samples using Support Vector Machines (SVM) and Neural Support Vector Machines (NSVM). Reported values include; Accuracy (Acc.), Sensitivity (Sens.), Specificity (Spec.), Precision (Prec.), Recall (Rec.), Area Under Curve (AUC), F-Measure (F), Kappa (κ) and Loss. All evaluation metrics (with the exception of loss) are recorded as values between 0 and 1, with 1 being the highest obtainable value. Values reported over 0.8 are considered an acceptable threshold for powerful classification models. Loss considers values closer to 0 as the most confident models.*

Sample	Algorithm	Acc.	Sens.	Spec.	Prec.	Rec.	AUC	F	κ	Loss
Pleistocene European Taxa	SVM	0.88	0.79	0.96	1.00	0.79	0.90	0.88	0.75	0.24
	NSVM	0.88	0.79	0.96	0.80	0.79	0.90	0.80	0.75	0.24
African Taxa	SVM	0.90	0.86	0.95	1.00	0.86	0.93	0.92	0.81	0.15
	NSVM	0.90	0.86	0.95	0.86	0.86	0.93	0.86	0.81	0.13
Taxonomic Family	SVM	0.92	0.80	0.95	1.00	0.80	0.80	0.72	0.82	0.35
	NSVM	0.81	0.69	0.91	0.72	0.69	0.84	0.70	0.61	0.43
Canidae	SVM	0.93	0.90	0.95	1.00	0.90	0.95	0.95	0.86	0.08
	NSVM	0.98	0.97	0.99	0.97	0.97	0.99	0.97	0.96	0.01
Felidae	SVM	0.97	0.96	0.98	1.00	0.96	0.98	0.98	0.94	0.05
	NSVM	0.97	0.96	0.98	0.96	0.96	0.98	0.96	0.94	0.14

Supplementary Table 24. *Classification results for individual species in the non-augmented Pleistocene European Taxa dataset. Reported values include; Accuracy (Acc.), Sensitivity (Sens.), Specificity (Spec.), Precision (Prec.), Recall (Rec.), Area Under Curve (AUC), F-Measure (F) and Loss. All evaluation metrics (with the exception of loss) are recorded as values between 0 and 1, with 1 being the highest obtainable value. Values reported over 0.8 are considered an acceptable threshold for powerful classification models. Loss considers values closer to 0 as the most confident models.*

Algorithm	Animal	Acc.	Sens.	Spec.	Prec.	Rec.	AUC	F	Loss
SVM	*U. arctos*	0.87	0.76	0.97	1.00	0.77	0.88	0.87	0.24
	V. vulpes	0.89	0.79	0.98	1.00	0.79	0.90	0.88	0.29
	C. crocuta	0.81	0.67	0.95	1.00	0.68	0.84	0.81	0.24
	P. pardus	0.87	0.77	0.96	1.00	0.77	0.89	0.87	0.30
	P. leo	0.91	0.92	0.90	1.00	0.92	0.96	0.96	0.13
	C. lupus	0.91	0.84	0.98	1.00	0.84	0.92	0.91	0.25
NSVM	*U. arctos*	0.85	0.76	0.96	0.74	0.76	0.88	0.75	0.35
	V. vulpes	0.91	0.81	0.97	0.84	0.81	0.91	0.83	0.12
	C. crocuta	0.86	0.75	0.94	0.78	0.75	0.87	0.77	0.21
	P. pardus	0.88	0.77	0.95	0.81	0.77	0.89	0.79	0.25
	P. leo	0.85	0.86	0.97	0.73	0.86	0.93	0.79	0.27
	C. lupus	0.91	0.81	0.96	0.87	0.81	0.91	0.84	0.27

Supplementary Table 25. *Classification results for individual species in the non-augmented African Taxa dataset. Reported values include; Accuracy (Acc.), Sensitivity (Sens.), Specificity (Spec.), Precision (Prec.), Recall (Rec.), Area Under Curve (AUC), F-Measure (F) and Loss. All evaluation metrics (with the exception of loss) are recorded as values between 0 and 1, with 1 being the highest obtainable value. Values reported over 0.8 are considered an acceptable threshold for powerful classification models. Loss considers values closer to 0 as the most confident models.*

Algorithm	Animal	Acc.	Sens.	Spec.	Prec.	Rec.	AUC	F	Loss
SVM	*C. crocuta*	0.87	0.76	0.98	1.00	0.77	0.88	0.87	0.20
	P. pardus	0.92	0.90	0.94	1.00	0.91	0.95	0.95	0.12
	L. pictus	0.89	0.81	0.96	1.00	0.81	0.91	0.90	0.17
	P. leo	0.93	0.95	0.92	1.00	0.95	0.97	0.97	0.11
NSVM	*C. crocuta*	0.87	0.80	0.93	0.80	0.80	0.90	0.80	0.24
	P. pardus	0.94	0.89	0.96	0.91	0.89	0.95	0.90	0.06
	L. pictus	0.89	0.83	0.94	0.84	0.83	0.91	0.83	0.14
	P. leo	0.92	0.91	0.97	0.87	0.91	0.95	0.89	0.08

Supplementary Table 26. *Classification results for individual species in the non-augmented Taxonomic Family dataset. Reported values include; Accuracy (Acc.), Sensitivity (Sens.), Specificity (Spec.), Precision (Prec.), Recall (Rec.), Area Under Curve (AUC), F-Measure (F) and Loss. All evaluation metrics (with the exception of loss) are recorded as values between 0 and 1, with 1 being the highest obtainable value. Values reported over 0.8 are considered an acceptable threshold for powerful classification models. Loss considers values closer to 0 as the most confident models.*

Algorithm	Animal	Acc.	Sens.	Spec.	Prec.	Rec.	AUC	F	Loss
SVM	Canidae	0.85	0.71	0.99	1.00	0.63	0.81	0.77	0.20
	Felidae	0.92	0.99	0.85	1.00	0.96	0.98	0.98	0.11
	Hyaenidae	0.94	0.88	1.00	1.00	0.37	0.68	0.54	0.57
	Ursidae	0.96	0.94	0.97	1.00	0.42	0.71	0.59	0.52
NSVM	Canidae	0.80	0.73	0.85	0.75	0.73	0.87	0.74	0.34
	Felidae	0.83	0.84	0.89	0.77	0.84	0.92	0.80	0.14
	Hyaenidae	0.80	0.60	0.94	0.66	0.60	0.80	0.63	0.55
	Ursidae	0.81	0.58	0.95	0.68	0.58	0.79	0.63	0.69

Supplementary Table 27. *Classification results for individual species in the non-augmented Canidae dataset. Reported values include; Accuracy (Acc.), Sensitivity (Sens.), Specificity (Spec.), Precision (Prec.), Recall (Rec.), Area Under Curve (AUC), F-Measure (F) and Loss. All evaluation metrics (with the exception of loss) are recorded as values between 0 and 1, with 1 being the highest obtainable value. Values reported over 0.8 are considered an acceptable threshold for powerful classification models. Loss considers values closer to 0 as the most confident models.*

Algorithm	Animal	Acc.	Sens.	Spec.	Prec.	Rec.	AUC	F	Loss
SVM	*V. vulpes*	0.92	0.87	0.98	1.00	0.87	0.93	0.93	0.11
	L. pictus	0.93	0.95	0.92	1.00	0.95	0.98	0.98	0.05
	C. lupus	0.92	0.89	0.96	1.00	0.89	0.94	0.94	0.08
NSVM	*V. vulpes*	0.98	0.98	0.99	0.96	0.98	0.99	0.97	0.01
	L. pictus	1.00	0.96	1.00	0.96	0.96	1.00	0.98	0.004
	C. lupus	0.98	0.97	0.99	0.97	0.97	0.99	0.97	0.01

Supplementary Table 28. *Classification results for individual species in the non-augmented Felidae dataset. Reported values include; Accuracy (Acc.), Sensitivity (Sens.), Specificity (Spec.), Precision (Prec.), Recall (Rec.), Area Under Curve (AUC), F-Measure (F) and Loss. All evaluation metrics (with the exception of loss) are recorded as values between 0 and 1, with 1 being the highest obtainable value. Values reported over 0.8 are considered an acceptable threshold for powerful classification models. Loss considers values closer to 0 as the most confident models.*

Algorithm	Animal	Acc.	Sens.	Spec.	Prec.	Rec.	AUC	F	Loss
SVM	*P. onca*	0.97	0.96	0.98	1.00	0.96	0.98	0.98	0.04
	P. pardus	0.97	0.95	0.99	1.00	0.95	0.98	0.98	0.05
	P. leo	0.96	0.96	0.96	1.00	0.96	0.98	0.98	0.06
NSVM	*P. onca*	0.98	0.99	0.99	0.96	0.99	0.99	0.97	0.10
	P. pardus	0.97	0.94	0.97	0.98	0.94	0.97	0.96	0.22
	P. leo	0.96	0.96	0.98	0.95	0.96	0.98	0.95	0.10

Supplementary Appendix 8 : Software

R (v.3.5.3, https://www.r-project.org/) and Python (v.3.7.4, https://www.python.org/) were the main programming languages were used for the present study. All applications were designed and implemented using a portable ASUS X550VX personal laptop (8 GB RAM, Intel® Core™ i5 6300HQ CPU (2.3 GHz), NVIDIA GTX 950 GPU), additionally employing Anaconda (v.4.7.12, https://www.anaconda.com/) for virtual environment and package management. JavaScript (v.1.8, https://www.javascript.com/), in combination with HTML (v.5) and CSS (v.3), was also used for some visualisation purposes.

Geometric Morphometrics

Geometric Morphometric applications were programmed in the R programming language, using the geomorph (v.3.3.1) and shapes (v.1.2.4) packages. Some statistical tests additionally used the RVAideMemoire (v.0.9) library. Phylogeny applications employed the phylotools (v.0.2.2), phytools (v.0.7-70), ctv (v.0.8-5) and ape (v.5.4) packages. Delaunay triangulations were performed in Python, using the scikit-learn (v.0.22.1, https://scikit-learn.org) library.

Statistical Applications

All statistical applications were programmed in the R programming language. Most statistical testing was performed in base R, while MANOVA was performed with the additional use of the RVAideMemoire (v.0.9) library.

Unsupervised Computational Learning

IFs and MCMCs were programmed using the R programming language. IFs used the solitude (v.1.1.1) library. MCMCs were programmed in base R, with the additional use of the sn (v.1.6-2) and genidst (v.2.0) libraries for skewed distribution modelling. Coda (v.0.19-4) was used for MCMC autocorrelation and ESS calculations. GANs were implemented in Python using TensorFlow (v.2.0.0, https://www.tensorflow.org/) on CPU. NumPy (v.1.17.3, https://numpy.org/) was also used for any additional numerical computations. Statistical evaluation of synthetic data results were performed in R, using the equivalence (v.0.7.2), TOSTER (v.0.3.4) and e1071 (v.1.7-1) libraries.

Supervised Computational Learning

SVMs were trained in the R (v.3.5.3) programming language, using primarily the caret (v.6.0-84), e1071 (v.1.7-1) and rBayesianOptimization (v.1.1.0) packages. The pROC (v.1.15.0) package was also used for model evaluation. NSVMs were implemented in Python (v.3.7.4), using TensorFlow (v.2.3.1) and scikit-learn (v.0.22.1). Fine tuning and bayesian optimization of NSVM were performed using HyperOpt and Hyperas (v.0.2.3). All applications were performed on CPU. Additional numerical computations were performed using NumPy (v.1.17.3).

Miscellaneous: Data handling and visualisation

Most data handling and visualisation applications were performed in R (v.3.5.3), using the tidyverse (v.1.3.0, https://www.tidyverse.org/) family of libraries. Among the tidyverse packages used; tibble (v.3.0.4), dplyr (v.1.0.2), and tidyr (v.1.1.2) were employed for data handling and management, while ggplot2 (v.3.3.2) was used for visualisation. In combination with ggplot2, the girdExtra (v.2.3) and ggpubr (v.0.2) packages were also used. Any additional visualisation techniques, specifically for probability density calculations, were performed using the sm (v.2.2-5.6) library.

Figures S4-S9 were created using base-R.

Data handling in python was performed using the pandas (v.0.25.2) library, while visualisation was performed primarily using matplotlib (v.3.1.1, https://matplotlib.org/), with some added features provided by seaborn (v.0.9.0, https://seaborn.pydata.org/). All additional numerical computations were performed using NumPy (v.1.17.3) or TensorFlow (v.2.0.0 or v.2.3.1).

Finally, JavaScript (v.1.8) was used for the creation of Figures 4, 5 and S3. Figures were created using the amCharts (v.4, https://www.amcharts.com/) library.

References

1. Domínguez-Rodrigo, M. *et al.* A new methodological approach to the taphonomic study of paleontological and archaeological faunal assemblages: a preliminary case study from Olduvai Gorge (Tanzania). *J. Archaeol. Sci.* **59**, 35–53, DOI: 10.1016/j.jas.2015.04.007 (2015).
2. Aramendi, J. *et al.* Who ate OH80 (Olduvai Gorge, Tanzania)? A geometric morphometric analysis of surface bone modifications of a *Paranthropus boisei* skeleton. *Quat. Int.* **517**, 118–130, DOI: 10.1016/j.quaint.2019.05.029 (2019).
3. Aramendi, J. *et al.* Discerning carnivore agency through the three-dimensional study of tooth pits: Revisiting crocodile feeding behaviour at FLK-Zinj and FLK NN3 (Olduvai Gorge, Tanzania). *Palaeogeogr. Palaeoclimatol. Palaeoecol.* **488**, 93–102, DOI: 10.1016/j.palaeo.2017.05.021 (2017).
4. Arriaza, M. C. *et al.* Geometric morphometric analysis of tooth pits and identification of felid and hyenid agency in bone modifications. *Quat. Int.* **517**, 79–87, DOI: 10.1016/j.quaint.2018.11.023 (2019).
5. Arriaza, M. C. *et al.* On applications of micro-photogrammetry and geometric morphometrics to studies of tooth mark morphology: The modern Olduvai Carnivore Site (Tanzania). *Palaeogeogr. Palaeoclimatol. Palaeoecol.* **488**, 103–112, DOI: 10.1016/j.palaeo.2017.01.036 (2017).
6. Yravedra, J. *et al.* The use of micro-photogrammetry and geometric morphometrics for identifying carnivore agency in bone assemblages. *J. Archaeol. Sci. Reports* **14**, 106–115, DOI: 10.1016/j.jasrep.2017.05.043 (2017).
7. Courtenay, L. A. *et al.* Combining machine learning algorithms and geometric morphometrics: a study of carnivore tooth marks. *Palaeogeogr. Palaeoclimatol. Palaeoecol.* **522**, 28–29, DOI: 10.1016/j.palaeo.2019.03.007 (2019).
8. Abellán, N., Jiménez-García, B., Aznarte, J., Baquedano, E. & Domínguez-Rodrigo, M. Deep learning classification of tooth scores made by different carnivores: achieving high accuracy when comparing African carnivore taxa and testing the hominin shift in the balance of power. *Archaeol. Anthropol. Sci.* **13**, 31, DOI: 10.1007/s12520-021-01273-9 (2021).
9. Yravedra, J., Lagos, L. & Bárcena, F. A taphonomic study of wild wolf *Canis lupus* modifications of horse bones in Northwestern Spain. *J. Taphon.* **9(1)**, 37–65 (2011).
10. Courtenay, L. A. *et al.* The effects of prey size on carnivore tooth mark morphologies on bone; the case study of *Canis lupus signatus*. *Hist. Biol.* DOI: 10.1080/08912963.2020.1827239 (2020).
11. Yravedra, J., Maté-González, M. Á., Courtenay, L. A., González-Aguilera, D. & Fernández-Fernández, M. The use of canid tooth marks on bone for the identification of livestock predation. *Sci. Reports* **9**, DOI: 10.1038/s41598-019-52807-0 (2019).
12. Yravedra, J., Andrés, M. & Domínguez-Rodrigo, M. A taphonomic study of the African wild dog (*Lycaon pictus*). *Archaeol. Anthropol. Sci.* **6**, 113–124, DOI: 10.1007/s12520-013-0164-1 (2014).
13. Courtenay, L. A. *et al.* Obtaining new resolutions in carnivore tooth pit morphological analyses: a methodological update for digital taphonomy. *PLoS ONE* **15(10)**, e0240328, DOI: 10.1371/journal.pone.0240328 (2020).
14. Moclán, A. *et al.* Identifying the bone-breaker at the Navalmaíllo Rock Shelter (Pinilla del Valle, Madrid) using machine learning algorithms. *Archaeol. Anthropol. Sci.* **12(2)**, 1–17, DOI: 10.1007/s12520-020-01017-1 (2020).
15. Yravedra, J., Andrés, M., Fosse, P. & Besson, J. P. Taphonomic analysis of small ungulates modified by fox (*Vulpes vulpes*) in Southwestern Europe. *J. Taphomomy* **12(1)**, 37–67 (2014).
16. Rodríguez-Alba, J. J., Linares-Matás, G. & Yravedra, J. First assessments of the taphonomic behaviour of jaguar (*Panthera onca*). *Quat. Int.* **517**, 88–96, DOI: 10.1016/j.quaint.2019.05.004 (2019).
17. Jiménez-García, B., Abellán, N., Baquedano, E., Cifuentes-Alcobendas, G. & Domínguez-Rodrigo, M. Corrigendum to "deep learning improves taphonomic resolution: high accruacy in differentiating tooth marks made by lions and jaguars". *J. Royal Soc. Interface* **17**, 20200782, DOI: 10.1098/rsif.2020.0782 (2020).
18. Domínguez-Rodrigo, M., Gidna, A. O., Yravedra, J. & Musiba, C. A comparative neo-taphonomic study of felids, hyaenids and canids: an analogical framework based on long bone modification patterns. *J. Taphon.* **10(3)**, 147–164 (2012).
19. Gidna, A., Yravedra, J. & Domínguez-Rodrigo, M. A cautionary note on the use of captive carnivores to model wild predator behavior: a comparison of bone modification patterns on long bones by captive and wild lions. *J. Archaeol. Sci.* **40**, 1903–1910 (2013).

20. Domínguez-Rodrigo, M. *et al.* Artificial intelligence provides greater accuracy in the classification of modern and ancient bone surface modifications. *Sci. Reports* **10**, 18862, DOI: 10.1038/s41598-020-75994-7 (2020).

21. Liu, F. T., Ting, K. M. & Zhou, Z. H. Isolation forest. In *2008 Eighth IEEE International Conference on Data Mining*, 413–422, DOI: 10.1109/ICDM.2008.17 (2008).

22. Goodfellow, I., Bengio, Y. & Courville, A. *Deep Learning* (MIT Press, Cambridge, 2016).

23. Goodfellow, I. *et al.* Generative adversarial nets. *Proc. Int. Conf. Neur. Inf. Process. Syst.* 2672-2680, arXiv: 1406.2661v1 (2014).

24. Lucic, M., Kurasch, K., Michalski, M., Bousquet, O. & Gelly, S. Are GANs created equal? A large scale study. *Proc. Int. Conf. Neur. Inf. Process. Syst.* 698-707, arXiv: 1406.2661v1 (2018).

25. Courtenay, L. A. & González-Aguilera, D. Geometric morphometric data augmentation using generative computational learning algorithms. *Appl. Sci.* DOI: 10.3390/app10249133 (2020).

26. Metropolis, N., Rosenbluth, A., Teller, A. & Teller, E. Equations of state calculations by fast computing machines. *J. Chem. Phys.* **21**, 1087–1092 (1953).

27. Hastings, W. Monte Carlo sampling methods using Markov chains and their application. *Biometrika* **57**, 97–109 (1970).

28. Gamerman, D. & Lopes, H. F. *Markov Chain Monte Carlo* (Chapman & Hall, Boca Raton, 2006).

29. Martin, O. *Bayesian Analysis with Python* (Packt, Birmingham, 2018).

30. Mao, X. *et al.* Least Squares Generative Adversarial Networks. *IEEE Int. Conf. Comp. Vision* (2017). arXiv: 1611.04076v3 (2017).

31. Arjovsky, M., Chintala, S. & Bottou, L. Wasserstein GAN. *Proc. Mach. Learn. Res.* **70**, 214-223, arXiv: 1701.07875v3 (2017).

32. Gulrajani, I., Ahmed, F., Arjovsky, M., Dumoulin, V. & Courville, A. Improved training for Wasserstein GANs. *Proc. Mach. Learn. Res.* arXiv: 1704.00028v3 (2017).

33. Fedus, W. *et al.* Many paths to equilibrium: GANs do not need to decrease a divergence at every step. *Int. Conf. Learn. Rep.* arXiv: 1710.08446 (2018).

34. Abu Bakar, S. A., Nadarajah, S., Adzhar, Z. A. & Mohamed, I. Gendist: an R package for generated probability distribution models. *PLoS ONE* **11(6)**, e0156537 (2016).

35. Azzalini, A. *The Skew-Normal and Related Families* (Cambridge University Press, Cambridge, 2014).

36. Höhle, J. & Höhle, M. Accuracy assessment of digital elevation models by means of robust statistical methods. *ISPRS J. Photogramm. Remote. Sens.* **64**, 398–406 (2009).

37. Kruschke, J. *Doing Bayesian Data Analysis*, vol. 2 (Academic Press, Burlington, MA, 2015).

38. Elvira, V., Martino, L. & Robet, C. Rethinking the effective sample size. *Preprint*. arXiv: 1809.04129v1 (2018).

39. Hauk, D. W. & Anderson, S. A new statistical procedure for testing equivalence in two-group comparative biovariability trials. *J. Pharmacokinet. Biopharm.* **12**, 83–91, DOI: 10.1007/BF01063612 (1984).

40. Yeun, K. K. The two-sample trimmed t for unequal population variances. *Biometrika* **61**, 165–170, DOI: 10.2307/2334299 (1974).

41. Cortes, C. & Vapnik, V. Support-vector networks. *Mach. Learn.* **20**, 273–297, DOI: 10.1007/BF00994018 (1995).

42. Bishop, C. *Pattern Recognition and Machine Learning* (Springer, Singapore, 2006).

43. Wiering, M. A. *et al.* The neural support vector machine. In *The 25th Benelux Artificial Intelligence Conference*, 257–254 (2013).

44. Jacot, A., Gabriel, F. & Hongler, C. Neural tangent kernel: Convergence and generalization in neural networks. *Proc. Int. Conf. Neur. Inf. Process. Syst.* **32**, 1-19, arXiv: 1806.07572 (2020).

45. Tancik, M. *et al.* Fourier features let networks learn high frequency functions in low dimensional domains. *Proc. Int. Conf. Neur. Inf. Process. Syst.* arXiv: 2006.10739v1 (2020).

46. Rahimi, A. & Recht, B. Random features for large-scale kernel machines. In *Proceedings of the International Conference of Neural Information Processing Systems*, vol. 20, 1–8, DOI: 10.5555/2981562.2981710 (2007).

47. Ramachandran, P., Zoph, B. & Le, Q. V. Swish: a self-gated activation function. *Google Brain*. arXiv: 1710.05941v1 (2017).

48. Kingma, D. P. & Ba, J. L. Adam: a method for stochastic optimization. *Int. Conf. Learn. Rep.* arXiv: 1412.6980 (2015).

49. Smith, L. N. Cyclical learning rates for training neural networks. In *IEEE Winter Conference on Applications of Computer Vision*, 464–472, DOI: 10.1109/WACV.2017.58 (2017).

50. Snoek, J., Larochelle, H. & Adams, R. P. Practical bayesian optimization of machine learning algorithms. *Proc. Int. Conf. Neur. Inf. Process. Syst.* **25**, 2951-2959, arXiv: 1206.2944 (2012).

51. Shahriari, B., Swersky, K., Wang, Z., Adams, R. P. & Freitas, N. Taking the human out of the loop: A review of bayesian optimization. *Proc. IEEE* **104(1)**, 148–175, DOI: 10.1109/JPROC.2015.2494218 (2016).

52. Bergstra, J., Bardenet, R., Bengio, Y. & Kégl, B. Algorithms for hyper-parameter optimization. *Proc. Int. Conf. Neural Inf. Process. Syst.* **24**, 2546–2554, DOI: 10.5555/2986459.2986743 (2011).

53. Bergstra, J. & Bengio, Y. Random search for hyper-parameter optimization. *J. Mach. Learn. Res.* **13(10)**, 281–305 (2012).

54. Christiansen, P. & Adolfssen, S. Bite forces, canine strength and skull allometry in carnivores (Mammalia, Carnivora). *J. Zool. Soc. Lond.* **266**, 133–151 (2005).

55. Thomason, J. J. Cranial strength in relation to estimated biting forces in some mammals. *Can. J. Zool.* **69(9)**, 2326–2333, DOI: 10.1139/z91-327 (1990).

56. Wroe, S., McHenry, C. & Thomason, J. Bite club: comparative bite force in big biting mammals and the prediction of predatory behaviour in fossil taxa. *Proc. Royal Soc. B* **272**, 619–625, DOI: 10.1098/rspb.2004.2986 (2005).

57. Christiansen, P. & Wroe, S. Bite forces and evolutionary adpatations to feeding ecology in carnivores. *Ecology* **88(2)**, 347–358 (2007).

58. Prado, J. M., Ortega, B., Palaus, X. & Tarrida, V. *Natura. Enciclopedia de los Animales. Carnívoros.* (Círculo de Lectores, Barcelona, 1984).

59. Gittleman, J. L. Carnivore body size: Ecological and taxonomic correlates. *Oceologia* **67**, 540–554 (1985).

60. Zhou, Y., Wang, S. R. & Ma, J. Z. Comprehensive species set revealing the phylogeny and biogeography of Feliformia (Mammalia, Carnivore) based on mitochondrial DNA. *PLoS ONE* **12(3)**, e0174902, DOI: 10.1371/journal.pone.0174902 (2017).

61. Nyakatura, K. & Bininda-Emonds, O. P. Updating the evolutionary history of Carnivora (Mammalia): a new species-level supertree complete with divergence time estimates. *BMC Biol.* **10**, 1–31 (2012).

62. Li, G., Davis, B. W., Eizirik, E. & Murphy, W. J. Phlogenomic evidence for ancient hybridization in the genomes of living cats (Felidae). *Genome Res.* **26**, 1–11, DOI: 10.1101/gr.186668.114 (2016).

63. Valkenburgh, B. V. & Ruff, C. B. Canine tooth strength and killing behaviour in large carnivores. *J. Zool. Soc. Lond.* **212**, 379–397 (1987).

64. Fidgett, A. L. & Plowman, A. Nutrition and diet evaluation. In Bishop, J., Hosey, G. & Plowman, A. (eds.) *Handbook of Zoo & Aquarium Research*, 154–175 (BIAZA, London, 2013).

65. Johnson, E. R. & Chant, D. C. Use of carcass density for determining carcass composition in beef cattle. *New Zealand J. Agric. Res.* **41(3)**, 325–333, DOI: 10.1080/00288233.1998.9513317 (1998).

66. Szalma, J. *et al.* The influence of the chosen in vitro bone simulation model on intraosseous temperatures and drilling times. *Sci. Reports* **9**, 11817, DOI: 10.1038/s41598-019-48416-6 (2019).

67. Lam, Y. M., Chen, X. & Pearson, O. M. Intertaxonomic variability in patterns of bone density and the differential representation of Bovid, Cervid and Equid elements in the archaeological record. *Am. Antiq.* **64**, 343–362 (1999).

68. Wasserstein, R. L., Schirm, A. L. & Lazar, N. A. Moving to a world beyond "$p < 0.05$". *The Am. Stat.* **73(Sup1)**, 1–19 (2019).

69. Wasserstein, R. L. & Lazar, N. A. The ASA statement on p-Values: Context, process, and purpose. *The Am. Stat.* **70(2)**, 129–133, DOI: 10.1080/00031305.2016.1154108 (2016).

70. Benjamin, D. J. & Berger, J. O. Three recommendations for improving the use of p-values. *The Am. Stat.* **73(Sup1)**, 186–191, DOI: 10.1080/0031305.2018.1543135 (2019).

71. Colquhoun, D. The False Positive Risk: a proposal concerning what to do about p-values. *The Am. Stat.* **73(Sup1)**, 192–201, DOI: 10.1080/00031305.2018.1529622 (2019).

72. Kennedy-Schaffer, L. Before p<0.05 to beyond p<0.05: Using history to contextualize p-values and significance testing. *The Am. Stat.* **73(Sup1)**, 82–90, DOI: 10.1080/00031305.2019.1537891 (2019).

73. Laplace, P. S. *Traité de Mécanique Céleste, Supplément* (Duprat, Paris, 1827).

74. Poisson, S. D. *Recherches sur la probabilité des Jugements en Matière Criminelle et en Matère Civile: Précédées des Règles Générales du Calcul des Probabilités* (Bachelier, Paris, 1837).

75. Pearson, K. X. On the criterion that a given system of deviations from the probable in the case of a correlated system of variables is such that it can be reasonably supposed to have arisen from random sampling. *The London, Edinburgh, Dublin Philos. Mag. J. Sci.* **50(302)**, 151–175 (1900).

76. Edgeworth, M. A. Methods of statistics. *J. Stat. Soc.* **41**, 181–217 (1885).

77. Elderton, W. P. Tables for testing the goodness of fit of theory to observation. *Biometrika* **1**, 155–163 (1902).

78. Student. The probable error of a mean. *Biometrika* **6**, 1–25 (1908).

79. Fisher, R. A. *Statistical Methods for Research Workers* (Oliver and Boyd, Edinburgh, 1925).

80. Neyman, J. & Pearson, E. S. The testing of statistical hypotheses in relation to probabilities a priori. *Math. Proc. Camb. Philos. Soc.* **29(4)**, 492–510 (1933).

81. Neyman, J. & Pearson, E. S. On the problem of the most efficient tests of statistical hypotheses. *Philos. Transactions Royal Soc. A* **231**, 289–337 (1933).

82. Held, L. & Ott, M. On p-Values and Bayes Factors. *Annu. Rev. Stat. its Appl.* **5(6)**, 1–27 (2018).

83. Bland, M. *An Introduction to Medical Statistics* (Oxford University Press, Oxford, 2015).

84. Cox, D. R. & Donnelly, C. A. *Principles of Applied Statistics* (Cambridge University Press, Cambridge, 2011).

85. Sellke, T., Bayarri, M. J. & Berger, J. O. Calibration of p values for testing precise null hypotheses. *The Am. Stat.* **55(1)**, 62–71 (2001).

86. Colquhoun, D. The reproducibility of research and the misinterpretation of p-values. *Royal Soc. Open Sci.* **4**, 171085, DOI: 10.1098/rsos.171085 (2017).

87. Laplace, P. S. *Théorie Analytique des Probabilités* (Courcier, Paris, 1823).

88. Gauss, C. F. *Theory of the Motion of Heavenly Bodies Moving about the Sun in Conic Sections (Theoria Motus Corporum Coelestium in sectionibus concis solem ambientium)* (Dover, New York, 1809).

89. Shapiro, S. S. & Wilk, M. B. An analysis of variance test for normality. *Biometrika* **52(3/4)**, 591–611 (1965).

90. Royston, P. Remark AS R94: A remark on algorithm AS181: The W-test for normality. *J. Royal Stat. Soc.* **44(4)**, 547–551 (1995).

91. Razali, N. M. Power comparisons of Shapiro-Wilk, Kolmogorov-Smirnov, Lilliefors and Anderson-Darling tests. *J. Stat. Model. Anal.* **2(1)**, 21–33 (2011).

92. Maas, A. L., Hannun, A. Y. & Ng, A. Y. Rectified nonlinearities improve neural network acoustic models. In *Proceedings of the 30th International Conference on Machine Learning*, 1–6 (2013).

93. Klambauer, G., Unterthiner, T. & Mayr, A. Self-normalizing neural networks. *Proc. Int. Conf. Neur. Inf. Process. Syst.* 1-102, arXiv: 1706.02515v5 (2017).

94. He, K., Zhang, X., Ren, S. & Sun, J. Delving deep into rectifiers: Surpassing human-level performance on imagenet classification. In *Proceedings of the IEEE International Conference on Computer Vision*, 1026–1034, DOI: 10.1109/ICCV.2015.123 (2015).

95. Glorot, X., Bordes, A. & Bengio, Y. Deep sparse rectifier neural networks. In *Proceedings of the 14th International Conference onf Artificial Intelligence and Statistics*, 315–323 (2011).
